# Instruction of haematopoietic lineage choices, evolution of transcriptional landscapes and cancer stem cell hierarchies derived from an AML1-ETO mouse model

**DOI:** 10.1002/emmm.201302661

**Published:** 2013-10-04

**Authors:** Nina Cabezas-Wallscheid, Victoria Eichwald, Jos de Graaf, Martin Löwer, Hans-Anton Lehr, Andreas Kreft, Leonid Eshkind, Andreas Hildebrandt, Yasmin Abassi, Rosario Heck, Anna Katharina Dehof, Svetlana Ohngemach, Rolf Sprengel, Simone Wörtge, Steffen Schmitt, Johannes Lotz, Claudius Meyer, Thomas Kindler, Dong-Er Zhang, Bernd Kaina, John C Castle, Andreas Trumpp, Ugur Sahin, Ernesto Bockamp

**Affiliations:** 1 Medical Center of the Johannes Gutenberg-University Mainz, Department of Internal Medicine III, Division of Translational and Experimental OncologyMainz, Germany; 2 German Cancer Research Center, Department of Stem Cells and CancerHeidelberg, Germany; 3 Medical Center of the Johannes Gutenberg-University Mainz, Institute for ToxicologyMainz, Germany; 4 German Cancer Research Center, Division of Molecular ImmunologyHeidelberg, Germany; 5 TRON – Translational Oncology at the Johannes Gutenberg-University MainzMainz, Germany; 6 University of Lausanne, Institut Universitaire de Pathologie, CHUVLausanne, Switzerland; 7 Medical Center of the Johannes Gutenberg-University Mainz, Department of PathologyMainz, Germany; 8 Johannes Gutenberg-University Mainz, Institute for InformaticsMainz, Germany; 9 Saarland University, Center for BioinformaticsSaarbrücken, Germany; 10 Max-Planck-Institute for Medical ResearchHeidelberg, Germany; 11 Medical Center of the Johannes Gutenberg-University Mainz, Institute for Molecular MedicineMainz, Germany; 12 German Cancer Research Center, FACS Core FacilityHeidelberg, Germany; 13 Medical Center of the Johannes Gutenberg-University Mainz, Institute of Clinical Chemistry and Laboratory MedicineMainz, Germany; 14 Medical Center of the Johannes Gutenberg-University Mainz Children's HospitalMainz, Germany; 15 Me, dical Center of the Johannes Gutenberg-University Mainz, Division of Haematology, Oncology and Pneumology, III. Medical DepartmentMainz, Germany; 16 Department of Pathology, University of California San Diego, Division of Biological Sciences and Moores UCSD Cancer CenterSan Diego, CA, USA

**Keywords:** cancer stem cells, core binding factor acute myeloid leukaemia, preclinical mouse model, therapy target validation, whole transcriptome sequencing

## Abstract

The t(8;21) chromosomal translocation activates aberrant expression of the AML1-ETO (AE) fusion protein and is commonly associated with core binding factor acute myeloid leukaemia (CBF AML). Combining a conditional mouse model that closely resembles the slow evolution and the mosaic AE expression pattern of human t(8;21) CBF AML with global transcriptome sequencing, we find that disease progression was characterized by two principal pathogenic mechanisms. Initially, AE expression modified the lineage potential of haematopoietic stem cells (HSCs), resulting in the selective expansion of the myeloid compartment at the expense of normal erythro- and lymphopoiesis. This lineage skewing was followed by a second substantial rewiring of transcriptional networks occurring in the trajectory to manifest leukaemia. We also find that both HSC and lineage-restricted granulocyte macrophage progenitors (GMPs) acquired leukaemic stem cell (LSC) potential being capable of initiating and maintaining the disease. Finally, our data demonstrate that long-term expression of AE induces an indolent myeloproliferative disease (MPD)-like myeloid leukaemia phenotype with complete penetrance and that acute inactivation of AE function is a potential novel therapeutic option.

## INTRODUCTION

Acute myeloid leukaemia (AML) is a heterogeneous group of severe haematological diseases characterized by a block in myeloid differentiation and the unrestrained proliferation of immature myeloid cells (Estey & Dohner, 2006). One of the most frequent genetic alterations found in human AML is the t(8;21)(q22;q22) AML1-ETO chromosomal translocation that is commonly associated with core binding factor (CBF) AML (Arber et al, 2008). Despite the great clinical improvements that have been made in the treatment of AML, t(8;21)-associated CBF AML remains a significant clinical problem, with 30% of patients relapsing and long-term survival rates ranging between 61 and 31% (Appelbaum et al, 2006; Döhner et al, 2010; Grimwade et al, 2010; Lin et al, 2008; Marcucci et al, 2005; Narimatsu et al, 2008).

In case of the t(8;21) translocation, a fusion between the DNA-binding *Runt* homology domain of the haematopoietic master regulator AML1 (RUNX1, CBFα2 and PEBPαB) and the ETO gene (RUNX1T1 or MTG8) generates the 752 amino acid long chimeric AML1-ETO (AE) protein (Miyoshi et al, 1993). Although functionally similar to the AML1 transcription factor, the AE fusion protein has a different sub-cellular localization, distinct biochemical and molecular properties and altered transcriptional activity (Lam & Zhang, 2012; Reikvam et al, 2011). Important insights into the molecular consequences of aberrant AE expression have been gained from microarray and chromatin immunoprecipitation experiments. In these studies, transcriptional AE target genes and epigenetic modifications were identified that link AE function to cellular proliferation, self-renewal and differentiation (Alcalay et al, 2003; Balgobind et al, 2011; Kvinlaug et al, 2011; Ptasinska et al, 2012; Ross et al, 2004; Valk et al, 2004). However, because these experiments were based on the analysis of direct transcriptional modifications promoted by short-term AE expression or deletion and on biopsies from AML patients, the stepwise evolution of transcriptome-wide alterations downstream of the initial t(8;21) translocation are essentially unknown. In order to understand the cellular programs operating during the trajectory to leukaemia and to define novel therapeutic agents that can interfere with these pathways, it is critical to analyse preclinical mouse models that recapitulate the stepwise evolution and the initial mosaic expression of AE in blood cells characteristic of the human disease.

In recent years leukaemic stem cells (LSCs) have attracted major attention as critical therapeutic targets as these cells have been proposed to drive leukaemia initiation, progression and maintenance (Baccelli & Trumpp, 2012; Dick, 2008). In addition, LSC are thought to be resistant to current chemotherapeutic regimes and thus might act as a reservoir for relapse (Ishikawa et al, 2007). For this reason, the identification and functional characterization of LSC has potentially profound clinical implications. Phenotypic, molecular and biochemical knowledge of LSC has been obtained for several AML subtypes. These studies demonstrated that AML LSC can be heterogeneous with respect to their cell surface phenotype and state of commitment [reviewed in (Horton & Huntly, 2012)]. However, the nature and molecular characteristics of t(8;21)-associated LSC still remain elusive.

Finally, it is not known if ablation of AE function during manifest AML will provide a benefit to the patient. Indeed, specific inhibition of a single leukaemia-maintaining factor can be a highly effective therapy for chronic myeloid leukaemia (CML), as illustrated by targeted therapeutics like Imatinib (Druker et al, 2006). Since AE expression is a recurrent clinical feature in CBF AML, the concept of targeting AE has been proposed and first strategies that specifically inactivate AE function have been reported (Barton et al, 2009; Wang et al, 2011; Wichmann et al, 2010). In order to evaluate the potential therapeutic benefit of AE ablation and to decide if further research in this direction is warranted, *in vivo* proof-of-principle experiments are required.

Using a novel experimental mouse model that recapitulates the slow disease evolution and mosaic expression of AE found in human AML, we report the first *in vivo* analysis of whole transcriptome alterations taking place immediately after the initial activation of AE and during the trajectory to leukaemic disease. We also show that the ability to initiate and maintain leukaemia is not only restricted to those cells that phenotypically resemble HSC but also resides in the granulocyte macrophage progenitor (GMP) population of committed myeloid cells. Finally, we demonstrate that long-term expression of AE consistently induces a phenotype of indolent myeloproliferative disease (MDP)-like myeloid leukaemia with complete penetrance and report that inactivation of AE function is a potential novel therapeutic option.

## RESULTS

### Generation of a blood cell-specific conditional AE mouse model

We initiated this study with the aim to analyse global signalling pathways and cellular mechanisms that operate during the progressive disease evolution downstream of the t(8;21) translocation. In the past several mouse models for t(8;21)-associated leukaemia have been developed [reviewed in (Lam & Zhang, 2012; Reikvam et al, 2011)]. However, with these models the initial mosaic expression pattern of AE and the delayed appearance of secondary mutations, characteristic for human CBF AML, were not recapitulated. To be able to address the role of AE during the dynamic evolution towards manifest leukaemia and to dissect the molecular and cellular programs operating during this process, we wanted to establish a model that allows to conditionally and reversibly express AE in a fraction of all blood cell types.

**Figure 1:**
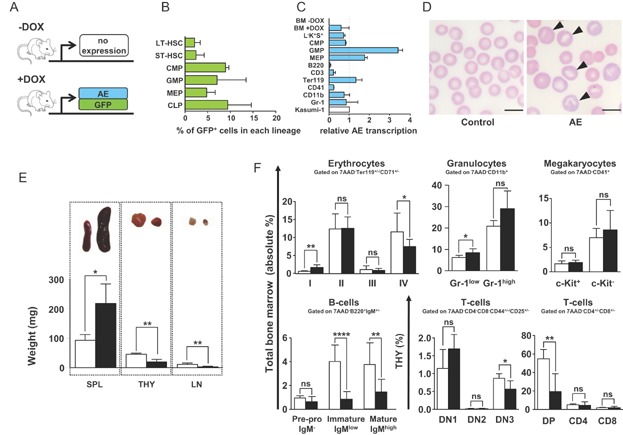
Conditional AE expression alters normal blood cell development.

To completely restrict AE expression to haematopoietic cells, we transplanted whole bone marrow (BM) from compound ROSA26-iM2-tetO-GFP/TgPtet-AML1-ETO (R26/AE) mice into lethally irradiated recipients (Rhoades et al, 2000; Wortge et al, 2010). As illustrated in Fig 1A, in reconstituted mice the expression of AE and the GFP co-reporter gene can be regulated by administering doxycycline (DOX) [for review see (Bockamp et al, 2002; Bockamp et al, 2008)]. We have previously shown that the R26-iM2 effector mouse strain directed mosaic transgene expression to lineage^−^/c-Kit^+^/Sca1^+^(L^−^K^+^S^+^) haematopoietic progenitor cells and to different adult blood lineages (Wortge et al, 2010). In line with this mosaic expression pattern, we found conditional GFP activation in a percentage of long-term repopulating haematopoietic stem cells (LT-HSC, L^−^K^+^S^+^CD150^+^CD48^−^ Flt3^−^CD34^−^), short-term repopulating haematopoietic stem cells (ST-HSC, L^−^K^+^S^+^CD150^+^CD48^−^Flt3^−^CD34^+^), common myeloid progenitors (CMPs, L^−^K^+^S^−^IL7Rα^−^CD34^+^FcγRII/III^low^), granulocyte macrophage progenitors (GMPs, L^−^K^+^S^−^IL7Rα^−^CD34^+^FcγRII/III^+^), megakaryocyte erythrocyte progenitors (MEPs, L^−^K^+^S^−^IL7Rα^−^CD34^−^FcγRII/III^low^) and common lymphoid progenitors (CLPs, LK^int^S^int^IL7Rα^+^) of R26/AE reconstituted recipients (Fig 1B, Supporting Information Fig S1A). Furthermore, analysis of median fluorescence revealed comparable GFP fluorescence in LT-, ST-HSC, CMP, GMP and MEP indicating that the R26-iM2 effector mouse model directed similar transgene activation levels to these populations (Supporting Information Fig S1B). To determine if AE induction was strictly dependent on the presence of DOX and to confirm AE transcription in different haematopoietic cell types, we subjected whole BM, immature and mature haematopoietic cells to quantitative rtPCR analysis. As shown in Fig 1C, in the absence of DOX AE mRNA was not detectable in BM cells of R26/AE animals (BM −DOX). By contrast, following DOX induction AE-specific transcripts were present in BM (BM +DOX), L^−^K^+^S^+^ cells, lineage-committed haematopoietic progenitors and adult blood cells (Fig 1C).

1. DOX-dependent AE and GFP expression.
2. Conditional GFP expression in blood stem and progenitor cells. Green bars represent the percentage of GFP-expressing cells ± SD in each lineage. Shown are mean values for four R26/AE BM-reconstituted recipients that had been DOX exposed during 10 days. LT-/ST-HSC, long-term/short-term repopulating haematopoietic stem cells; CMP, common myeloid progenitor; GMP, granulocyte macrophage progenitor; MEP, megakaryocyte erythroid progenitor; CLP, common lymphoid progenitor.
3. Blue bars show mean AE mRNA levels ± SD from three compound R26/AE mice in the absence (−DOX) or after 3 days of DOX exposure (+DOX). AE mRNA was determined in BM, L^−^K^+^S^+^ cells, CMP, GMP, MEP, B220^+^ B-lymphoid cells, CD3^+^ T-lymphoid cells, Ter119^+^ erythrocytes, CD41^+^ megakaryocytes, CD11b^+^ monocytes and macrophages and Gr1^+^ myeloid cells. AE levels are represented relative to AE mRNA transcription in the Kasumi-1 cell line (white bar).
4. Peripheral blood films from a representative control and an AE-expressing mouse. Arrowheads indicate abnormal red blood cells.
5. Above the bar diagram a spleen (SPL), thymus (THY) and lymph node (LN) of a non-induced (left) and an AE-expressing mouse (right) are shown. Each bar represents the mean weight ± SD for five individual mice. White bars represent non-induced and black bars AE-expressing mice. Two-tailed unpaired *t*-test analysis demonstrated significant differences in spleen (*p* = 0.0331), thymus (*p* = 0.0021) and lymph node (*p* = 0.0093) weight between control and AE-induced mice.
6. Flow cytometric analysis of erythroid, granulocytic, magakaryocytic, B- and T-cell maturation profiles from controls (white bars) and mice that have been DOX-induced (black bars, +DOX nine months). For each cell type the absolute percentages ± SD of cells in the BM or the thymus are shown. Roman numerals designate different stages of erythrocyte maturation: I, proerythroblasts; II, basophilic erythroblasts; III, late basophilic erythroblasts and chromatophilic erythroblasts; IV, orthochromatophilic erythroblasts and reticulocytes. 7AAD, 7-amino-actinomycin D; DN, double negative and DP, double positive for CD4 and CD8 surface markers. * *p* < 0.05; ** *p* < 0.01; **** *p* < 0.0001; ns, not significant.

Taken together, the finding that conditional GFP activation was restricted to a minor percentage of LT-, ST-HSC and blood cell progenitors further extended previous results and demonstrated that the ROSA26 promoter activated transgene expression only in a limited number of cells within the stem and progenitor cell compartment. Our findings also provide direct evidence that the R26/AE transplantation model is suitable for conditionally activating AE expression in blood cells with no background.

### AE expression alters normal haematopoiesis

A growing body of evidence suggests that the t(8;21) translocation is only leukaemia-initiating but not sufficient for the induction of AML (Kelly & Gilliland, 2002; Lam & Zhang, 2012; Reikvam et al, 2011). The constant association between the t(8;21) translocation and AML furthermore argues that aberrant AE rearrangement in HSC and thus also in their more differentiated progeny will change normal haematopoiesis in a way that specifically favours the later development of AML. To assess downstream effects promoted by AE expression and to evaluate the time needed to induce a phenotype, haematopoietic parameters were monitored. In agreement with previous studies (Buchholz et al, 2000; Higuchi et al, 2002; Rhoades et al, 2000; Yuan et al, 2001), AE expression for 6 months did not produce any obvious effects in blood cell parameters and organ morphology. Conversely, between 8 and 10 months, we noticed first phenotypic alterations. Analysis of differential blood cell parameters between AE-expressing and non-induced control mice revealed a significant drop in peripheral red blood cells that was accompanied by reduced levels of haemoglobin and an increase in eosinophils and large peroxidase-negative cells (Supporting Information Fig S2). Furthermore, inspection of peripheral blood morphology documented atypical red blood cells characterized by a polychromatic, aniso- and poikilocytotic phenotype (Fig 1D, arrowheads). In addition, we noticed that AE-expressing animals presented marked splenomegaly and a significant reduction of the thymus and the lymph nodes (Fig 1E). Histological analysis of thymus and lymph nodes revealed no additional abnormalities. By contrast, AE spleens showed clear signs of extramedullary haematopoiesis and in particular an increase in erythropoiesis within the red pulp (Supporting Information Fig S3 arrowheads). Flow cytometry directly confirmed the accumulation of erythrocytes in AE spleens and documented a significant increase in CD11b^+^/Gr1^+^ granulocytes but not in B lymphocytes and L^−^K^+^S^+^ cells (Supporting Information Fig S4).

Consistent with the immature morphology of peripheral erythrocytes and revealing a defect in red blood cell maturation, BM pro-erythroblasts were increased and the relative percentage of more mature orthochromatophilic erythroblasts and reticulocytes was significantly reduced in AE expressing animals (I and IV, Fig 1F, Supporting Information Fig S5A). Moreover, immature and the relative percentage of mature granulocytes were augmented while megakaryocytes did not significantly change (Fig 1F, Supporting Information Fig S5B and C). With regard to lymphopoiesis, we found a decrease in immature and mature B cells and a reduction in DN3 and CD4^+^/CD8^+^ double positive (DP) T lymphoid cells (Fig 1F, Supporting Information Fig S6). Collectively, these data provide direct *in vivo* evidence that mosaic expression of AE in blood cells for more than 6 months altered normal haematopoietic lineage development and specifically increased the output of myeloid cells at the expense of erythro- and lymphopoiesis.

### AE expression specifically increases lineage-committed myeloid progenitors

**Figure 2:**
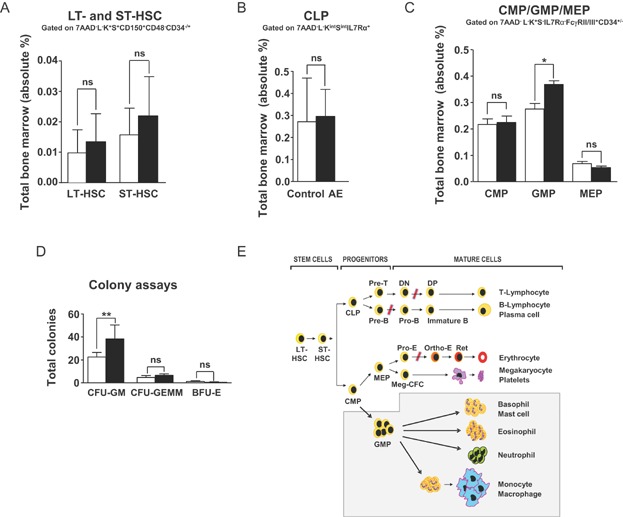
AE expression specifically increases GMP.* *p* < 0.05; ** *p* < 0.01; ns, not significant.

Adoptive transfer experiments using retrovirus-transduced BM cells suggested that enforced AE expression expanded the number of HSC (de Guzman et al, 2002). However, in this study HSC were defined as L^−^K^+^S^+^ cells, which represent a heterogeneous group of immature blood cells, and well defined HSC subgroups like LT-, ST-HSC and blood cell progenitors were not explicitly analysed. To establish the relevance of blood cell-specific AE induction for the HSC compartment, we analysed LT-, ST-HSC and haematopoietic progenitors. Interestingly, DOX exposure for 8–10 months did not significantly change LT- and ST-HSC (Fig 2A, Supporting Information Fig S7). Equally, CLP were not affected (Fig 2B, Supporting Information Fig S8A). However, in AE-expressing mice, GMP were significantly expanded and the relative percentages of MEP as well as CMP were reduced (Fig 2C, Supporting Information Fig S8B). In line with the increase in myelopoiesis found *in vivo* and also in agreement with previous reports (de Guzman et al, 2002; Higuchi et al, 2002; Okuda et al, 1996; Rhoades et al, 2000; Schwieger et al, 2002) we observed a significant increase in *ex vivo* granulocyte macrophage (CFU-GM) colony formation (Fig 2D). These results demonstrated that AE induction did not significantly increase LT-, ST-HSC and blood cell progenitors but specifically expanded the GMP population.

1. Flow cytometric analysis of LT- and ST-HSC showing the absolute percentages ± SD obtained from control (white bars) and DOX-induced mice (black bars).
2. Flow cytometric analysis of CLP showing the absolute percentages ± SD of CLP cells in the BM of control (white bar) and AE-induced mice (black bar).
3. Flow cytometric analysis of GMP, CMP and MEP showing the absolute percentages ± SD of each progenitor cell type in the BM of control (white bars) and AE-induced mice (black bars).
4. Colony forming assay of BM cells from controls (white bars) and mice that had been DOX-induced during eight months (black bars). Bars represent the mean values ± SD for three individual mice. Granulocyte macrophage (CFU-GM), mixed haematopoietic (CFU-GEMM) and erythroid burst-forming (BFU-E) colonies are shown. Two-tailed unpaired *t*-test analysis demonstrated significant differences in CFU-GM (*p* = 0.0015) but not in CFU-GEMM (*p* = 0.1099) and BFU-E (*p* = 0.0519) colony numbers between control and AE-induced BM cells.
5. Overview of developmental alterations following AE expression for more than 6 month. Red slashes indicate impaired lineage maturation. A grey background marks the expanded myeloid compartment.

To reveal potential differences in cells with activated transgene expression, we separately analysed GFP^+^ and GFP^−^ HSC and progenitors. Gating on GFP^+^ and GFP^−^ cells revealed no significant differences between the relative percentages of control, GFP^−^ and GFP^+^ LT-, ST-HSC and CLP (Supporting Information Fig S9A and B). Conversely, although control and GFP^−^ CMP, GMP, MEP and GFP^+^ CMP were not significantly different, GFP^+^ GMP were augmented and GFP^+^ MEP decreased (Supporting Information Fig S10). These findings highlighted differences between GFP^−^ and GFP^+^ GMP and MEP populations and thus support the interpretation that transgene activation promoted the expansion of GMP and reduced MEP in a cell-autonomous fashion. Importantly, the finding that LT- and ST-HSC as well as CLP and CMP did not significantly differ between controls, GFP^−^ and GFP^+^ populations provides direct *in vivo* evidence that conditional transgene induction did not expand the number of HSC, CLP or CMP pools in our mice. Figure 2E summarizes how mosaic AE expression altered normal haematopoiesis in our model.

### Long-term AE expression induces an indolent myeloproliferative disease (MPD)-like myeloid leukaemia phenotype in mice

**Figure 3:**
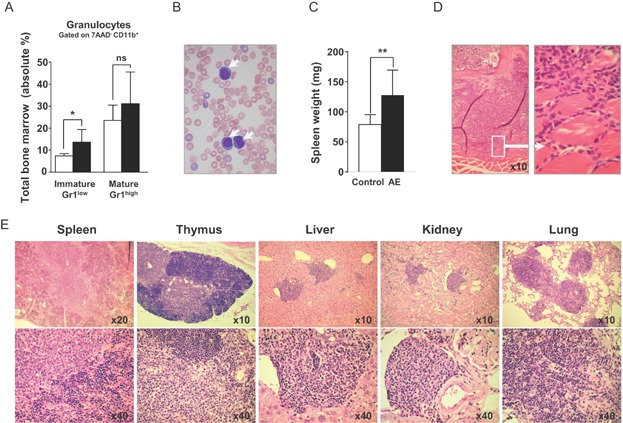
Long-term AE expression induces leukaemia. With the exception of Fig 3D data shown was obtained from long-term reconstituted mice without DOX or animals that have been exposed to DOX for 16–18 months. * *p* < 0.05; ** *p* < 0.01; ns, not significant.

In patients the t(8;21) translocation can persist for years before any signs of AML become apparent (Kusec et al, 1994; Miyamoto et al, 1996; Nucifora et al, 1993; Saunders et al, 1994; Wiemels et al, 2002). To establish whether long-term AE expression induces leukaemia, we analysed R26/AE reconstituted mice at later time points. After 16–18 months, all animals had high white blood cell counts, increased immature BM granulocytes and circulating blasts in the periphery (Fig 3A and B, Supporting Information Fig S11). Moreover, AE-expressing mice had splenomegaly (Fig 3C) and an elevated number of leukaemic cells in the BM already invading neighbouring tissues (Fig 3D). In contrast to three age-matched long-term reconstituted controls that had not been induced with DOX and indicative for invasive disease, we found infiltrates in spleen, thymus, liver, kidney and lung of diseased animals (Fig 3E). These results provide first *in vivo* evidence that persistent AE expression promoted an invasive MPD-like myeloid leukaemia phenotype in mice (Kogan et al, 2002).

1. The absolute percentages ± SD of immature Gr1^low^ and mature Gr1^high^ granulocytic cells in the BM of reconstituted controls (white bars) and DOX-induced mice (black bars) are shown.
2. Peripheral blood film from an AE-expressing mouse with blasts (arrows).
3. Bar diagram showing the spleen weight ± SD from eight control (white bar) and eight long-term induced (black bar) mice. Two-tailed unpaired *t*-test analysis demonstrated significant differences in spleen weight between control and AE-induced mice (*p* = 0.0052).
4. Images show sections through the bone marrow (BM) of the spinal cord taken from a mouse that was DOX-induced during 22 months. The left image shows an overview and the right picture a magnification revealing the invasion of leukaemic cells into the surrounding muscle tissue.
5. Representative H&E-stained organ sections from leukaemic mice demonstrating the invasion of cells into spleen, thymus, liver, kidney and lung. Pictures at the bottom show a detail of the above image at a higher magnification.

**Figure 4:**
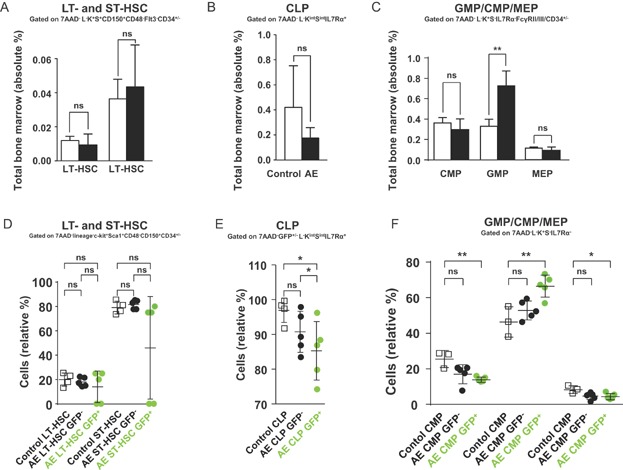
Analysis of HSC and progenitor populations in leukaemic mice. Data shown was obtained from reconstituted mice without DOX or animals that have been exposed to DOX for 16–18 months. 7AAD, 7-amino-actinomycin D; * *p* < 0.05; ** *p* < 0.01; ns, not significant.

We next wanted to know whether LT-, ST-HSC or lineage-restricted progenitor pools were altered in diseased mice. Remarkably, we did not detect any significant differences in LT- and ST-HSC between AE expressing and non-induced BM reconstituted R26/AE animals (Fig 4A, Supporting Information Fig S12). Equally, the number of CLP was not significantly changed (Fig 4B, Supporting Information Fig S13A). However, the total and relative percentages of GMP were expanded and the relative percentages of CMP and MEP were reduced (Fig 4C, Supporting Information Fig S13B). When we analysed GFP^−^ and GFP^+^ HSC and progenitors, we also found no significant differences between GFP^−^ and control ST-, LT-HSC and lineage-restricted progenitors (Fig 4D–F, Supporting Information Fig S14 and S15). Similar to GFP^−^ HSC, GFP^+^ LT- and ST-HSC were not increased and we even detected in two animals a drastic reduction of these cells (Fig 4D). By sharp contrast, the GFP-expressing GMP population was increased and relative percentages of GFP^+^ CLP, CMP and MEP were significantly reduced (Fig 4E and F, Supporting Information Fig S14 and S15). These results clearly show that the overall pool of LT- and ST-HSC did not expand upon long-term transgene activation and proof that disease induction was not accompanied by and did not depend on the specific expansion of HSC. In addition, our findings suggest that the expansion of GMP and the reduction of relative CMP and MEP percentages were sustained by cells with activated transgene expression. The ability to consistently induce a leukaemic phenotype in all AE-expressing animals furthermore demonstrated that secondary molecular events, required for the onset of manifest disease, did take place within the time frame of the experiment.

1. The bar diagrams depict absolute percentages ± SD of LT- and ST-HSC in the BM of controls (white bars) and leukaemic mice (black bars).
2. Absolute percentages ± SD of CLP cells in the BM from controls (white bar) and leukaemic mice (black bar).
3. Absolute percentages ± SD of CMP, GMP and MEP in the BM from controls (white bar) and leukaemic mice (black bar).
4. Flow cytometric analysis of LT- and ST-HSC from controls (white squares), GFP^−^ (black dots) and GFP^+^ (green dots) leukaemic animals. LT- and ST-HSC frequencies are represented as relative percentage within the 7AAD^−^, GFP^+/−^, lineage^−^, c-Kit^+^, Sca1^+^, CD150^+^, CD48^−^, CD34^+/−^ BM fraction.
5. Flow cytometric analysis of control (white squares) or GFP^−^ (black dots) and GFP^+^ (green dots) CLP obtained from leukamic mice. CLP frequencies are represented as relative percentage within the 7AAD^−^, GFP^+/−^, lineage^−^, c-Kit^int^, Sca1^int^, IL7 receptor α^+^ BM fraction.
6. Flow cytometric analysis of controls (white squares) or GFP^−^ (black dots) and GFP^+^ (green dots) CMP, GMP and MEP obtained from leukaemic mice. CMP are represented as relative percentage of CD34^+^, Fcγ receptor II/III^low^, GMP as CD34^+^, Fcγ receptor II/III^+^ and MEP as CD34^−^, Fcγ receptor II/III^low^ cells within the 7AAD^−^, GFP^+/−^, lineage^−^, c-Kit^+^, Sca1^−^, IL7 receptor α^−^ BM fraction.

### Ablation of AE reverts the leukaemic phenotype

**Figure 5:**
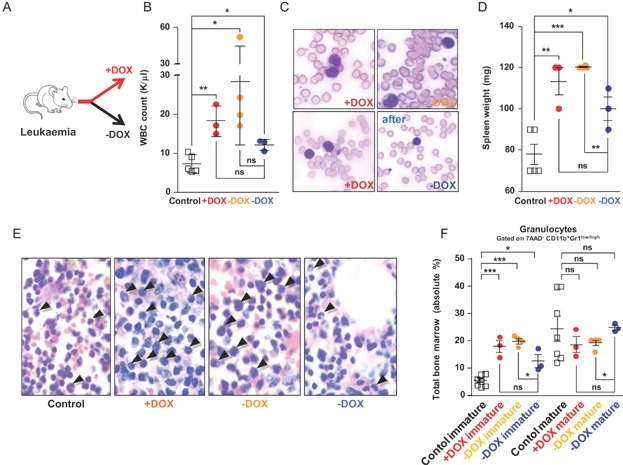
Ablation of AE function in leukaemic mice. Each square and dot represents one mouse. * *p* < 0.05; ** *p* < 0.01; *** *p* < 0.001; ns, not significant.

The common association between CBF AML and the t(8;21) translocation suggests a direct role of AE for the pathogenesis of this disease. However, it is not known if continued AE expression is essential for maintaining leukaemia. We therefore asked what would be the consequence of an acute ablation of AE function during overt disease. To this end long-term induced leukaemic BM cells together with supportive BM were injected into lethally irradiated recipients that were continued on DOX. Of note, by choosing a fairly advanced AE induction time, we sought to mimic a progressed disease stage that might have a high probability to be refractory to therapy. Three month after transplantation all mice showed abnormal peripheral blood parameters and had circulating blasts. At this point, seven out of ten animals were switched to a DOX-free diet (Fig 5A). Four month later all animals were analysed. As expected, mice with continued AE expression showed manifest disease progression evidenced by high WBC counts (+DOX, Fig 5B), circulating blasts (+DOX, Fig 5C upper left image) and splenomegaly (+DOX, Fig 5D). In addition, we found elevated numbers of BM blasts (+DOX, Fig 5E arrowheads), a significant increase in Gr1^low^ immature myeloid cells and a decrease of the relative percentage of Gr1^high^ more mature granulocytes (+DOX, Fig 5F, Supporting Information Fig S16). Interestingly, four mice in the DOX-free group also progressed with leukaemia (−DOX orange in Fig 5B–F, Supporting Information Fig S16). However, three out of seven previously leukaemic mice that had been shifted to a DOX-free diet, showed signs of recovery. In these animals we observed a drop in WBC counts (−DOX blue dots in Fig 5B) and a nearly complete loss of circulating blasts in the periphery (−DOX blue, compare the peripheral blood films of the same mouse before and after the DOX switch in Fig 5C). Moreover, all three mice presented a trend towards reduced spleen size (−DOX blue, Fig 5D) and we detected less BM blasts (−DOX blue, Fig 5E). Directly confirming the decrease of immature blasts in the BM and indicating a shift towards more normal myelopoiesis, we found in two of the three mice less immature Gr1^low^ granulocytes and a higher relative percentage of more mature Gr1^high^ granulocytes (−DOX blue, Fig 5F, Supporting Information Fig S16). Collectively, these results indicated that in three previously leukaemic mice AE function was required to maintain all features of overt disease and that in two mice the acute ablation of AE did lead to an almost complete regression of the malignant phenotype. These results are also consistent with the hypothesis that secondary transforming events needed for leukaemia maintenance will differ in individual mice and thus render some animals insensitive to AE ablation.

1. Experimental design.
2. Mean WBC counts ± SD from controls (white squares), continuously DOX-exposed (+DOX) mice, animals that had been shifted to a DOX-free diet with disease progression and animals with signs of recovery (−DOX). Two-tailed unpaired *t*-test analysis demonstrated significant differences in WBC counts between controls and continuously DOX-exposed mice (*p* = 0.0023), controls and animals that had been shifted to a DOX-free diet with disease progression (*p* = 0.0339) and controls and animals with signs of recovery (*p* = 0.0181). No statistically significant differences in WBC counts were detected between continuously DOX-exposed mice and recovered animals (*p* = 0.0639) and also between animals that had been shifted to a DOX-free diet with disease progression and recovered animals (*p* = 0.2059).
3. The upper panel shows representative peripheral blood films from a mouse that was permanently exposed to DOX (+DOX) or shifted to a DOX-free diet and progressing with leukaemia (−DOX). The two images at the bottom show peripheral blood films from the same mouse before (+DOX, left image) and 16 weeks after the animal had been shifted to a DOX-free diet (−DOX, right image).
4. Individual spleen weight of control mice (white squares) and animals that were either continuously DOX-exposed (+DOX, red dots), shifted to a DOX-free diet and progressing with leukaemia (−DOX, orange dots) or shifted to a DOX-free diet and showing signs of recovery (−DOX, blue dots). Two-tailed unpaired *t*-test analysis demonstrated significant differences in spleen weight between controls and continuously DOX-exposed mice (*p* = 0.0049), controls and animals that had been shifted to a DOX-free diet with disease progression (*p* = 0.0001), controls and animals with signs of recovery (*p* = 0.03) and animals that had been shifted to a DOX-free diet with disease progression and recovered animals (*p* = 0.0086). No statistically significant difference in spleen weight between continuously DOX-exposed mice and recovered animals (*p* = 0.2051) was detected.
5. Bone marrow (BM) sections through the spinal cord of a control mouse, a mouse continuously exposed to DOX (+DOX), an animal shifted to a DOX-free diet with progressing leukaemia (−DOX, orange) and a mouse shifted to a DOX-free diet showing signs of recovery (−DOX, blue). Black arrowheads indicate immature myelocytes/blast-like cells. Note the increase of big blast-like cells in the two sections in the middle and the decrease of these cells in the section on the right (−DOX, blue).
6. Flow cytometric analysis of immature (CD11b^+^Gr1^low^) and more mature (CD11b^+^Gr1^high^) cells within the BM population of granulocytes for control mice (white squares), animals continuously receiving DOX (+DOX, red dots), animals that had been shifted to a DOX-free diet presenting signs of progressing leukaemia (−DOX, orange dots) and mice that had been shifted to a DOX-free diet presenting signs of recovery (−DOX, blue dots).

### Both cells with an HSC and lineage-committed GMP immunophenotype acquire LSC properties

**Figure 6:**
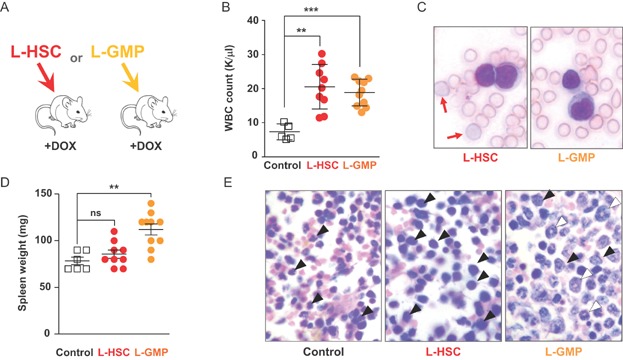
L-HSC and L-GMP acquire leukaemic stem cell potential. ** *p* < 00.01; *** *p* < 0.001; ns, not significant. Each square and dot represents one mouse.

Several recent studies reported the isolation and characterization of AML LSC [reviewed in (Horton & Huntly, 2012)]. However, for t(8;21)-associated leukaemia the cell surface phenotype and the state of commitment of LCS remain elusive. To establish whether only cells expressing HSC markers or also cells with a more lineage-restricted immune phenotype can act as LSC in our model, we tested the potential of leukaemic HSC (L-HSC defined as L^−^K^+^S^+^CD150^+^ containing both malignant and normal HSC) and leukaemic GMP (L-GMP defined as L^−^K^+^S^−^IL7Rα^−^CD34^+^FcγRII/III^+^ containing both malignant and normal GMP) to propagate the disease. Following a previously established protocol (Cozzio et al, 2003), we injected either L-HSC or L-GMP together with normal BM cells into irradiated recipients (Fig 6A). Seven weeks later, we found GFP expression in both myeloid and lymphoid cells of mice that were reconstituted with L-HSC cells. By contrast, in L-GMP-transplanted animals GFP expression was restricted to myeloid cells (Supporting Information Fig S17). At 5–7 months after transplantation we noticed elevated WBC counts (Fig 6B) and circulating blasts (Fig 6C) in L-HSC- and L-GMP-reconstituted mice. However, only L-HSC-reconstituted mice presented atypical red blood cells directly documenting the limited lineage potential of L-GMP (Fig 6C red arrows). At this time several L-GMP-injected animals but only one L-HSC-reconstituted mouse had already proceeded to manifest splenomegaly (Fig 6D). Further confirming the leukaemic phenotype, we observed a high frequency of large BM blasts primarily consisting of immature forms in L-HSC-reconstituted mice (Fig 6E, black arrowheads) and both immature (black arrowheads) and also more mature ring forms (white arrowheads) in L-GMP-reconstituted mice. In accordance with the high frequency of myeloid BM cells, both L-HSC and L-GMP recipients had a high number of GFP^+^CD11b^+^Gr1^+^ cells (Supporting Information Fig S18A). Also in line with the different composition of immature und more mature myeloblasts seen in the BM sections of Fig 6E, we found increased relative percentages of immature Gr1^low^ granulocytes in both L-HSC- and L-GMP-reconstituted mice and a significant drop of Gr1^high^ mature granulocytes in L-HSC animals (Supporting Information Fig S18B). Because in mice adoptively transferred GMP can produce myeloid progeny for only a limited period of about four weeks (Akashi et al, 2000), the long-term production of GFP^+^ BM myeloblasts and the lasting presence of circulating blast in L-GMP-reconstituted mice thus indicated that the transplanted L-GMP had lost their limited capacity to self-renew. In summary our results provide direct evidence that cells with an HSC immune phenotype and also more differentiated GMP-like cells acquired LSC properties, that L-HSC and G-GMP LSC had different cellular outputs and that the two populations coexisted in mice.

1. Experimental design.
2. Shown are mean WBC counts ± SD from controls (white squares), L-HSC- (red dots) and L-GMP-transplanted (orange dots) mice. Two-tailed unpaired *t*-test analysis demonstrated significant differences in WBC counts between controls and mice that have been reconstituted with L-HSC (*p* = 0.0001) or L-GMP (*p* < 0.0001).
3. Peripheral blood films from L-HSC- (left image) and L-GMP-transplanted (right image) animals containing circulating blasts. Of note, only the L-HSC-reconstituted peripheral blood contained abnormal erythrocytes (red arrows).
4. Individual spleen weight of control (white squares), L-HSC- (red dots) and L-GMP-transplanted (orange dots) mice. Two-tailed unpaired *t*-test analysis demonstrated a significant difference in spleen weight between controls and mice that have been reconstituted with L-GMP (*p* = 0.0012) but not between controls and L-HSC-reconstituted animals (*p* = 0.2782).
5. Sections through the BM of the spinal cord of control animals (left), L-HSC- (central) and L-GMP-transplanted (right) mice. Black arrowheads indicate immature myelocytes/blast-like cells white arrowheads point to more mature ring forms.

### Whole transcriptome RNA-Seq analysis of normal, AE-expressing and leukaemic GMP cells

**Figure 7:**
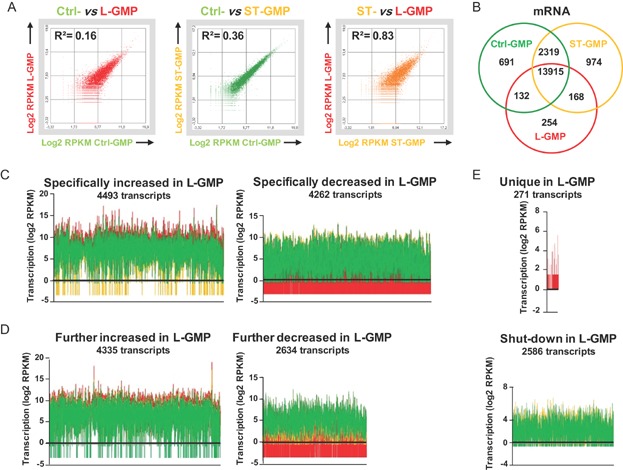
RNA-Seq whole transcriptome analysis.

Data presented so far indicate that in the R26/AE model L-GMP cells will undergo leukaemic transformation and acquire LSC properties. Because knowledge about the transcriptional changes and cellular programs that GMP will engage during the trajectory from a normal to a leukaemic state are missing, we sought to determine these changes at a global scale. To this end GMP (defined as L^−^K^+^S-IL7Rα^−^CD34^+^FcγRII/III^+^) from non-AE-expressing controls (Ctrl-GMP), from mice that had been short-term DOX-induced during 10 days (ST-GMP) and from overtly leukaemic animals (L-GMP) were sorted and subjected to whole transcriptome sequencing (RNA-Seq). After alignment to the mouse reference genome and normalization, we identified a total number of 18097, 18435 and 15181 transcripts in Ctrl-, ST- and L-GMP (see Supporting Information Table S1 for a complete list of all transcripts). This information was used to evaluate differences between each GMP pool. As seen in Fig 7A, Pearson's correlation analysis revealed the highest transcriptional divergence (*R*^2^ = 0.1602) between Ctrl- and L-GMP, whereas Ctrl- and ST-GMP transcriptomes had a smaller correlation-based distance (*R*^2^ = 0.3623) and ST- and L-GMP were most similar (*R*^2^ = 0.8328). These results document an incremental transcriptional shift between Ctrl-, ST- and L-GMP and demonstrate that global transcriptome changes immediately following conditional AE expression were stronger than those occurring during the transition from ST- to L-GMP. To further define molecular differences in gene expression, we compared common and differentially expressed mRNAs. Comparison between Ctrl-, ST- and L-GMP mRNA pools documented that the majority of gene coding transcripts were commonly expressed in all three groups (13915) and identified 691 uniquely expressed transcripts for Ctrl-, 974 for ST- and 254 for L-GMP (Fig 7B and Supporting Information Table S2).

1. Scatter plots of Ctrl- *versus* L-GMP (left plot), Ctrl- *versus* ST-GMP (central plot) and ST- *versus* L-GMP (right plot) transcriptomes. *R*^2^, Pearson's correlation value; Ctrl, control; ST-, short-term; L-GMP, leukaemic-GMP; RPKM, reads per kilobase of exon model per million mapped reads.
2. Venn diagram with commonly and differentially transcribed mRNAs. Numbers of individual mRNA transcripts are indicated.
3. Transcription of genes that were specifically increased (left plot) or decreased (right plot) only in the L-GMP population. The transcriptional activity is shown as log2 RPKM values. L-GMP transcripts are indicated in red, ST-GPM transcripts are depicted in orange and control GMP transcripts are represented in green.
4. Transcription of genes that were already up- or downregulated in ST-GMP and further increased (left plot) or decreased (right plot) in the L-GMP population.
5. Transcriptional profiles of genes that are uniquely expressed (above) or specifically shut down (below) in L-GMP.

To better understand global transcriptional changes critical for leukaemia induction downstream of the initial AE activation, we grouped the L-GMP transcriptome into six different subclasses (Fig 7C–E and Supporting Information Table S3). This classification was based on the assumption that expression of genes needed or beneficial for the induction and maintenance of the leukaemic phenotype should be specifically increased, further upregulated or newly transcribed in L-GMP cells. Conversely, the expression of genes detrimental or irrelevant for leukaemia should be specifically downregulated, further decreased or completely extinguished in L-GMP. Remarkably, we found 4493 transcripts that were specifically increased and 4262 transcripts that were specifically decreased only in the L-GMP population (Fig 7C red). This finding indicates that the majority of individual transcriptional changes occurring in L-GMP were not promoted through the initial activation of AE but did occur later. We also found 4335 transcripts, initially upregulated and 2634 transcripts downregulated following short-term AE expression, that were also further in- and decreased in leukaemia (Fig 7D red). Moreover, in L-GMP 271 transcripts were uniquely expressed and 2566 transcripts were completely shut down (Fig 7E).

We conclude from these data that the initial expression of AE induced the majority of genome-wide transcriptome alterations. These initial transcriptome changes were followed by a second substantial transcriptional fine tuning that took place during leukaemic transformation. These findings provide the first dynamic picture documenting the evolution of the transcriptional landscape downstream of the initial AE activation towards leukaemia. Our results also offer a conclusive explanation for the fact that rearranged HSC clones carrying the t(8;21) translocation can persist in a non-leukaemic mode for years before the transcriptional rewiring required for leukaemic transformation has occurred and malignant disease progression can start off.

### L-GMP engage general cancer-associated pathways and share major gene expression changes with human t(8;21) AML patients

The above determination of global transcriptome changes provides the interesting opportunity to identify specific pathways that are needed for leukaemic transformation and disease progression. To gain more insight into these programs, transcripts only deregulated, extinguished or newly activated in L-GMP were subjected to Ingenuity Pathway Analysis (IPA). Validation of these gene sets unveiled striking changes in cancer-related networks. As shown in Table1, IPA revealed a significant upregulation of cellular pathways associated with transformation, survival, proliferation, RNA expression and cytoplasmic organization that was accompanied by a downregulation of cell death pathways (see Supporting Information Table S4 for the complete gene list). These findings are thus in line with the observed phenotype and provide direct insight into the substantial molecular rewiring that did take place in the trajectory to manifest disease.

**Table:** Ingenuity pathway analysis (IPA) reveals upregulation of cancer-related cellular networks and downregulation of cell death pathways

Function	Transcription in L-GMP	Predicted Activation	p-Value	Z-score	Molecules
Transformation	Specifically increased or newly expressed	Increased	1.42E-07	5.219	138
	Specifically decreased or shut-down	Increased	2.59E-03	5.030	115
Survival	Specifically increased or newly expressed	Increased	5.45E-05	10.778	305
	Specifically decreased or shut-down	Increased	7.17E-09	10.760	327
Proliferation	Specifically increased or newly expressed	Increased	4.01E-11	9.965	860
	Specifically decreased or shut-down	Increased	3.54E-09	6.445	834
Expression of RNA	Specifically increased or newly expressed	Increased	1.26E-09	2.976	546
	Specifically decreased or shut-down	Increased	1.74E-03	4.431	485
Cytoplasmatic Organization	Specifically increased or newly expressed	Increased	8.87E-07	5.747	331
	Specifically decreased or shut-down	Increased	6.21E-06	6.207	321
Cell Death	Specifically increased or newly expressed	Decreased	1.72E-08	−2.395	777
	Specifically decreased or shut-down	no prediction	4.50E-12	−1.031	794

Analysis of L-GMP-specific gene expression showed a significant increase of pathways associated with transformation, survival, proliferation, RNA expression and cytoplasmatic organization and a downregulation of pathways associated to cell death. Gene function, transcriptional modulation in L-GMP as compared to Ctrl-GMP, the IPA predicted functional activation status, *p*-values, Z-scores and the number of mapped individual molecules are indicated.

**Figure 8:**
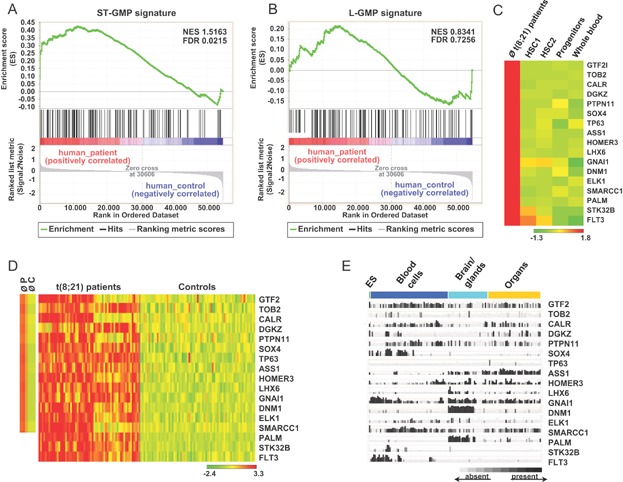
Gene set enrichment analysis (GSEA) and identification of L-GMP-specific transcripts.

We next wondered whether the most prominent gene expression changes that were identified in our mice also apply to human AML patients. To this end, we interrogated publicly available gene expression datasets for human t(8;21) CBF AML patients and healthy controls (Balgobind et al, 2011; de Jonge et al, 2010; Mills et al, 2009; Verhaak et al, 2009). Using gene set enrichment analysis (GSEA), we found that genes highly upregulated or exclusively expressed in murine short-term induced ST-GMP strongly correlated with the expression of their orthologous human genes sets (*p* = 0.0215) in t(8;21) AML patients (Fig 8A; Supporting Information Table S5). Equally, genes that were highly over-expressed in murine L-GMP were also positively enriched in human t(8;21) AML patients (Fig 8B; Supporting Information Table S5). These results establish that genes that were significantly over-expressed or newly switched on in our mouse model were also deregulated in human patients.

1. GSEA showing significant enrichment (*p*-value 0.0215) of the top 150 murine ST-GMP-associated gene set differentially expressed between human t(8;21) CBF AML patients and non-leukaemic controls.
2. GSEA showing enrichment of the top 150 murine L-GMP-associated gene set differentially expressed between human t(8;21) CBF AML patients and non-leukaemic controls. In A and B the enrichment plot is shown at the top. Black vertical lines indicate gene hits and the ranking metric scores based on a *t*-test analysis are depicted as a grey line plot. NES, normalized enrichment score.
3. Gene expression heat map of 17 L-GMP-specific genes with each column representing the difference in expression between median transcription values in 61 t(8;21) CBF AML patients (red lane), sorted primary human CD34^+^/CD38^−^ cord blood HSC (HSC1), CD34^+^/CD38^low^/CD36^−^ cord blood HSC (HSC2), primary human CD34^+^/CD38^+^ haematopoietic progenitors (Progenitors) and unsorted Lin^+/−^ human primary cord blood cells (Whole blood), obtained from previously published micro-array data (Eppert et al, 2011). Relative transcription values are colour coded from red (high) through yellow (intermediate) to green (low).
4. Gene expression heat map of 17 L-GMP-specific genes with each column representing the difference in expression between transcription values. The first two columns show median transcription values for 61 t(8;21) patients and 69 healthy control donors. On the right 61 individual gene transcription profiles from t(8;21) AML biopsies (t(8;21) patients) and 69 individual gene transcription profiles from healthy donors (Controls) are shown. Relative transcription values are colour coded from red (high) through yellow (intermediate) to green (low).
5. Expression of 17 L-GMP-specific genes in normal human embryonic and adult tissues. Relative transcription values are colour coded from black (expressed) through grey (intermediate) to white (absent).

### Identification of genes specifically expressed in murine L-GMP and t(8;21) AML patient samples but absent or poorly transcribed in normal human blood cells

Targeted therapies aimed at eradicating transformed cells and in particular LSC will greatly benefit from the identification of novel genes that are exclusively expressed in LSC but absent from normal cell types. We reasoned that all genes which were uniquely expressed (Fig 7E) or specifically increased (Fig 7C) in murine L-GMP cells and also being on average at least twofold upregulated in human t(8;21) AML samples as compared to healthy donors (Balgobind et al, 2011; de Jonge et al, 2010; Mills et al, 2009; Verhaak et al, 2009) might represent possible targets for therapy. To exclude unwanted toxic effects, these transcripts should also be missing or only be weakly expressed in HSC, progenitors and normal blood cells. As seen in Fig 8C, among all 4767 L-GMP transcripts that were exclusively expressed or specifically upregulated in L-GMP cells, we identified 17 individual transcripts that were absent or poorly expressed in publicly available gene expression datasets for normal human HSC, progenitors and haematopoietic cells (Eppert et al, 2011). Equally, all 17 genes were on average found to be transcribed in the t(8;21) AML patient cohort but were only poorly transcribed in healthy donors (first two lanes of the heat map in Fig 8D). Analysis of individual transcription profiles revealed furthermore that gene expression levels in different patients varied and that some transcripts were also present in control individuals (Fig 8D). Finally, we wished to determine the expression levels of the 17 candidate genes in normal embryonic and adult human tissues. The result of this survey is shown in Fig 8E and indicates that none of the 17 candidate genes was restricted to leukaemic cells. Taken together, trans-species comparison of transcripts uniquely expressed or specifically upregulated in murine L-GMP, identified 17 candidate genes that were predominantly upregulated in human t(8,21) AML samples but poorly expressed in normal haematopoietic cells.

## DISCUSSION

To better understand the multifactorial nature of cancer and to develop new and possibly more effective therapeutic strategies, mouse models that accurately mirror the human syndrome will become increasingly important. Here we present an experimental model that faithfully recapitulates the slow disease progression and the mosaic AE expression pattern characteristic for human t(8;21) CBF AML. Analysis of this model revealed several key cellular and molecular mechanisms operating immediately downstream of AE and during leukaemic progression. In addition, we applied next generation sequencing to unveil the dynamics of global transcriptome changes occurring during the trajectory to overt disease. Our findings provide direct evidence that the succession of events that take place downstream of the t(8;21) chromosomal translocation is essentially characterized by two basic mechanisms: lineage instruction of HSC followed by the substantial fine adjustment of transcriptional programs needed for the progression to leukaemia.

Our experiments demonstrate that expression of AE changes the developmental output of HSC by promoting the selective expansion of myeloid cells at the expense of normal erythroid and lymphoid lineage maturation. These results further extend previous reports about AE-specific lineage skewing (de Guzman et al, 2002; Fenske et al, 2004; Higuchi et al, 2002; Schwieger et al, 2002) and provide the plausible explanation why the t(8;21) translocation is commonly associated with myeloid leukaemias (expansion of the myeloid compartment) but absent from erythroid and lymphoid malignancies (impairment of erythroid, B- and T-cell maturation). Furthermore, we provide direct *in vivo* evidence – both during pre-leukaemia and manifest disease – that aberrant AE activation specifically expanded the GMP population but that despite an activated DOX-switch in LT- and ST-HSC the pool of HSC did not increase. By gating on the GFP^+^ and GFP^−^ cells, our results further suggest that the expansion of GMP and the reduction of other progenitor populations are substantially sustained by the transgene expressing population. Our results thus lead to the concept that the main function of aberrant AE expression is to modify the lineage potential of HSC resulting in the selective expansion of the myeloid compartment.

Data from several previous models have suggested that AE expression is not sufficient for inducing myeloid leukaemia (Buchholz et al, 2000; Higuchi et al, 2002; Rhoades et al, 2000; Schessl et al, 2005; Schwieger et al, 2002; Yuan et al, 2001). The reason why AE activation failed to induce leukaemia in these studies is not obvious but might be explained by too short AE expression times or the use of transgenic and viral promoters that failed to recapitulate the cell-type specificity and/or the AE expression levels needed for leukaemia induction in mice. However, if we consider that AE activation is commonly associated with CBF AML and that many patients with disease recurrence carry the t(8;21) translocation during remission, then it is important to resolve the question whether long-term AE expression will invariably lead to leukaemia. Here, we provide direct experimental proof that mosaic expression of AE for long periods induces an MPD-like myeloid leukaemia phenotype with complete penetrance in mice. However, even though our diseased animals showed several hallmarks of leukaemic disease, they did not die even after prolonged periods of DOX-induction. The reason for this lack of mortality is in line with previous reports (Buchholz et al, 2000; Higuchi et al, 2002; Rhoades et al, 2000; Schessl et al, 2005; Schwieger et al, 2002; Yuan et al, 2001) and might be explained by the fact that in our model AE expression was restricted to a subfaction of blood cells or that the natural history of events from the initial activation of AE expression to lethality requires more time than we had provided in our experiments. Although differences between mice and men surely have to be considered, the fact that long-term expression of AE induced a leukaemic phenotype has almost certainly direct therapeutic implications. Because human t(8;21)-rearranged clones can be expected to eventually acquire a similar long-term leukaemogenic potential as their murine counterparts, we would argue that the complete eradication of these clones from the patient will be crucial to reach complete disease remission.

One clinically important but completely unsettled issue is whether AE function is necessary for leukaemia maintenance. Here, we find that the acute ablation of AE function during progressive disease induced regression of the malignant phenotype. This proof-of-principle experiment is key to future drug development efforts because it demonstrates that targeting AE function can provide a direct benefit. We also found that some mice did not respond to AE inactivation. This conundrum highlights the general limits of single factor therapies in the context of an *in vivo* setting and is in line with clinical observations in which the majority of cancer patients will eventually become resistant to targeted therapies (Lackner et al, 2012). Although we did not address the molecular mechanisms underlying the lack of disease regression upon DOX withdrawal, several possibilities including major chromosomal aberrations, specific leukaemia-associated genetic and/or epigenetic alterations or the incomplete extinction of AE expression in resistant clones have to be envisaged. Taken together, we predict that targeting AE can be a promising therapeutic option. However, because secondary mutations initiating the onset of overt leukaemia are heterogeneous in t(8;21) AML patients (Hatlen et al, 2012), it will be crucial to develop reliable diagnostic markers to identify those patients that will benefit from AE inactivation.

At the functional level, we demonstrate that both HSC and lineage-restricted GMP-like cells acquired LSC properties. This observation agrees with reports from other AML subtypes in men and mice (Horton & Huntly, 2012) and suggests that AML LSC are generally heterogeneous with respect to their cell surface phenotype and state of commitment. A very important finding emerging from our experiments is that L-GMP had lost their reduced self-renewal potential indicated by the long-term production of immature myeloblasts and also more mature ring forms in the BM. Conversely, L-HSC predominantly produced more immature blasts. These findings thus demonstrated that LSC with different maturation status and cellular outputs exist in mice. Since human AML LSC are very likely to also retain multiple cellular and molecular traits of their cells of origin, it is to be expected that HSC- and GMP-derived patient LSC might react differently to treatment and thus show different clinical responses to standard chemotherapy. Although intriguing and relevant for therapy, a more definitive demonstration of this possibility awaits additional studies.

In the present study we report the first analysis of transcriptome-wide changes that occur immediately after AE activation and during leukaemic transformation. Although important insights into the molecular consequences of AE activation have been previously gained, published experiments focused mainly on the analysis of direct transcriptional changes promoted by short-term AE activation or ablation or the genome-wide analysis of human AML biopsies (Alcalay et al, 2003; Balgobind et al, 2011; Kvinlaug et al, 2011; Ptasinska et al, 2012; Ross et al, 2004; Valk et al, 2004). Our RNA-Seq whole transcriptome experiments broadly extend these studies and provide for the first time a global view of the stepwise alterations occurring during disease progression in mice. For example, correlation analysis comparing the transcriptome changes at early and late points showed that the majority of global transcriptional changes occurred after short-term AE activation. However, our data also revealed a second substantial wave of transcriptional rewiring that took place during the transition to manifest disease. Highlighting the leukaemogenic nature of secondary events in L-GMP, IPA identified pathways associated with oncogenic transformation and the prevention of apoptosis. The identification of leukaemia-associated pathways, specific to long-term AE-expressing L-GMP cells raises the interesting possibility that targeting key regulators in these pathways may be useful to specifically abolish leukaemia-sustaining programs. However, additional transcriptional profiles especially those derived from AML patients during remission and recurrence are needed to identify novel functionally relevant targets.

When working with animal models, it is important to develop and exploit those systems that are most relevant for the human disease. To test if not only the observed phenotype but also major gene expression changes were conserved between our model and human AML, we interrogated publicly available gene expression profiles from t(8;21) AML patients and healthy individuals. Examining sets of genes that were highly upregulated in murine cells, we found that many orthologous human genes were also significantly upregulated in t(8;21) AML patients. These findings indicate that not only the phenotypic manifestation of the disease but also major alterations in gene expression were similar between human patients and our model. Using transcripts selectively upregulated in murine L-GMP, we identified 17 orthologous human genes that were predominantly expressed in t(8;21) AML patients, poorly transcribed in healthy donors and absent from or only marginally transcribed in normal haematopoietic cells. The identification of candidates fulfilling these criteria suggests that at least some of these genes might represent future therapeutic targets and that interfering with their function should not produce unwanted haematotoxic effects.

In summary, using a model that recapitulates the slow disease evolution and mosaic expression of AE in t(8;21) CBF AML, we report several molecular and cellular principles governing the dynamic disease progression downstream of the initial activation of AE. The exhaustive analysis presented here thus represents a good starting point for developing and testing novel therapeutic strategies needed for a better and more effective treatment of t(8;21)-associated leukaemia.

## MATERIALS AND METHODS

### Mice

ROSA26-iM2-GFP (R26) and the TgPtet-AML1-ETO (AE) mouse lines were on a C57BL/6 background and have been previously described (Rhoades et al, 2000; Wortge et al, 2010). To induce the expression of AE, animals were administered 2 mg DOX/ml H_2_O or 600 mg DOX/kg as food pellets. All experiments were approved by the Ethical Committee of the Landesuntersuchungsamt Rheinland-Pfalz under the reference number 23 177-07/G 09-1-047.

### Flow cytometry

Cells acquisition was performed on a FACSCalibur, LSR II or Canto II (BD) and analysed with the FlowJo software (TreeStar). Cell sorting was performed on a FACSAria I cell sorter (BD). A detailed description of all flow cytometric materials and methods can be found online in the Supporting Information section.

### RNA-Seq

RNA-Seq data are available in the ArrayExpress database (http://www.ebi.ac.uk/arrayexpress) under E-MTAB-1820 and under the ENA Accession Number ERP003759. A detailed description of the RNA-Seq materials and methods can be found online in the Supporting Information section.

### Statistical analysis

For calculating *R*^2^ values Pearson's correlation analysis was performed. For statistical data analysis the two-tailed unpaired *t*-test was applied using the GraphPad Prism software (Version 5.0, GraphPad).

### All other Materials and Methods

A description of Materials and Methods for quantitative real-time PCR, BM transplantation, isolation and functional characterization of LSC, acute ablation of AE expression in leukaemic mice, colony-forming unit assay, routine haematologic assays and histopathology and the conversion and comparison of murine ST AE- and leukaemia-associated transcripts with human t(8;21) CBF AML and non-leukaemic profiles is available in the online the supplemental material section.

### The paper explained

PROBLEM:

Determinants governing initiation, progression and establishment of t(8;21)-associated leukaemia have not been studied in a mouse model that recapitulates the slow disease evolution and mosaic AE expression found in patients. Moreover, the dynamics of progressive transcriptome alterations and the nature of LSC are unknown. Finally, proof-of-principle experiments testing the therapeutic benefit of AE inactivation are missing.

RESULTS:

Using a mouse model that closely recapitulates the slow pathogenesis of human CBF AML we report the evolution of transcriptome-wide alterations following AE activation. AE expression initially promoted the specific expansion of myeloid cells followed by secondary transcriptional changes associated with the upregulation of leukaemia-promoting and anti-apoptotic programs. We also find that both HSC- and GMP-like cells act as LSC. Finally, we demonstrate that long-term expression of AE induced leukaemia and that inactivation of AE function is a potential novel therapeutic option.

IMPACT:

Our results reveal several novel mechanisms operating downstream of the initial AE activation and proof that both cells with HSC and GMP markers can initiate and maintain leukaemia in mice. The finding that inactivation of AE function is a potential therapeutic option will warrant the future development of AE-specific therapeutic strategies.

## Author contributions

EB, LE, TK, CM, BK, JCC, AT and US conceived the idea; EB, AH, NC-W and JC wrote the paper; NC-W performed most of the experiments of the paper; NC-W, VE, LE, YA, RH and SO performed mouse experiments; RS, SW and D-EZ contributed genetically modified mice, JdG and NC-W performed the NGS experiments; ML, AH, AKD and JCC performed the bioinformatics analysis; AL, AK and JL performed the pathology; NC-W and SS performed the flow cytometric analysis.

## For more information

1. http://www.macmillan.org.uk/Cancerinformation/Cancertypes/Leukaemiaacutemyeloid/AML.aspx
2. http://www.cancer.gov/cancertopics/pdq/treatment/adultAML/Patient/page1
3. http://tron-mainz.de/

## Notes

As a service to our authors and readers, this journal provides supporting information supplied by the authors. Such materials are peer reviewed and may be re-organized for online delivery, but are not copy-edited or typeset. Technical support issues arising from supporting information (other than missing files) should be addressed to the authors.
