# Supplementary material for: Instruction of haematopoietic lineage choices, evolution of transcriptional landscapes and cancer stem cell hierarchies derived from an AML1-ETO mouse model
Source: EMBO Mol Med. 2013 Oct 4;5(12):1804–20. doi: 10.1002/emmm.201302661 (PMC3914523; doi:10.1002/emmm.201302661)
Supplement: Supplementary file 1 [file emmm0005-1804-sd1.pdf]

EMBO Molecular Medicine

Instruction of haematopoietic lineage choices, evolution of transcriptional landscapes and cancer stem cell hierarchies derived from an AML1-ETO mouse model

Nina Cabezas-Wallscheid, Victoria Eichwald, Jos de Graaf, Martin Löwer, Hans-Anton Lehr, Andreas Kreft, Leonid Eshkind, Andreas Hildebrandt, Yasmin Abassi, Rosario Heck, Anna Katharina Dehof, Svetlana Ohngemach, Rolf Sprengel, Simone Wörtge, Steffen Schmitt, Johannes Lotz, Claudius Meyer, Thomas Kindler, Dong-Er Zhang, Bernd Kaina, John C. Castle, Andreas Trumpp, Ugur Sahin and Ernesto Bockamp

Corresponding author: Ernesto Bockamp, Johannes Gutenberg Universität Mainz

Review timeline:

Submission date:	20 February 2013
Editorial Decision:	21 April 2013
Revision received:	02 July 2013
Editorial Decision:	16 July 2013
Revision received:	28 August 2013
Accepted:	28 August 2013

Transaction Report:

(Note: With the exception of the correction of typographical or spelling errors that could be a source of ambiguity, letters and reports are not edited. The original formatting of letters and referee reports may not be reflected in this compilation.)

Editor: Céline Carret

1st Editorial Decision 21 April 2013

Thank you for the submission of your manuscript to EMBO Molecular Medicine. We have now heard back from the three referees whom we asked to evaluate your manuscript.

As you will see from the reports below, the referees find the topic of your study interesting and relevant. However, they all raise significant concerns on your work, which should be convincingly addressed in a major revision of the present manuscript.

As you will see from the enclosed reports, all three referees question the model and make suggestions to improve the quality of the data. They also regret that a detailed and explicit method is lacking, making it difficult to assess the data properly and verify whether the claims are supported by the findings. They would like to see AE expression rather than GFP positive measure, FACS scatterplots, numbers rather than percentages, phenotypic description of the leukemia, and death curves.

We do feel that the manuscript would be greatly improved by clearer explanation of the methodology and better demonstration of the cause and effect relationships and therefore would strongly suggest to address all issues raised as best as you possibly can.

Given this, we would be willing to consider a revised manuscript with the understanding that the referee concerns must be fully addressed and that acceptance of the manuscript would entail a second round of review.

I should remind you that it is EMBO Molecular Medicine policy to allow a single round of revision only and that, therefore, acceptance or rejection of the manuscript will depend on the completeness of your responses included in the next, final version of the manuscript.

I realize however, that addressing the referees' comments in full would involve a lot of additional experimental work and I am uncertain whether you will be able (or willing) to return a revised manuscript within a reasonable time-frame. I would also understand your decision if you chose to rather seek rapid publication elsewhere at this stage.

I look forward to seeing a revised form of your manuscript as soon as possible.

Should you find that the requested revisions are not feasible and choose, therefore, to submit your paper elsewhere, we would welcome a message to this effect.

***** Reviewer's comments *****

Referee #1 (Comments on Novelty/Model System):

Comment: The proposed model seems promising but in my opinion there are still a few issues that need to be addressed (see detailed review)

Referee #1 (Remarks):

In this study the Authors examine the effect of the conditional expression of a TET inducible version of the human oncogene AML1-ETO in the development of AML and how its acute withdrawal affects the course of disease.
The general strategy is thought through and tries to answer important questions that are central to the cancer biology field.
The Authors took advance of a previously established transgenic mouse (Rhoades et al) and crossed it with a GFP coupled TET inducible system (Woertge et al) which allows both to express the oncogene and to monitor the level of induction mediated by doxycicline administration.

Few comments on the model system.
In principle and overall, the usage of TET inducible systems in vitro allows the fine tuning of gene expression, and facilitate the switching on / off of a gene of interest to assess the effects of acute up / down gene regulation. However, the use of these systems in vivo poses great problems in terms of bioavailability and clearance of the inducer over time, and, differently from the in vitro setting, there is a limit in the number of parameters one can change to comply with the experimental requirements.
A previous publication (Wortge et al.) from the same group established and described the ROSA26-iM2-GFP reporter background strain, showing that doxycicline mediated level of AE induction was limited and it varied in the different hematopoietic compartments.
This premise is important because some of the conclusions the Authors came up with may be somehow affected by this intrinsic heterogeneity.

General concerns

1) No lethality data nor detailed immunophenotyping of the leukaemias are shown in order to establish how consistent are the different tumours among each other and if they mimic the human t(8;21) hallmarks.
2) AML1-ETO expression in full blown leukaemia is not shown;
3) The Authors use EGFP positivity as a quantitative readout of engraftment but, as said above, this

is not a reliable readout for it. In their case, it would be much better to use the ly5.1 / ly5.2 syngeneic mouse model for adoptive BM transplant.

Specific points:

- Fig. 1B: The Authors examine the % of EGFP+ cells in the different subcellular compartments in transplanted mice kept under DOX treatment. Which is the real % of engraftment in those mice? Is the percentage of EGFP+ cells similar to the expected one or not?
- The methods for this experiment and others are not clearly written.

From the BM transplantation section: "For analysing pre-leukaemic phenotypes RAG2-/- mice and for leukaemic phenotypes RAG2-/- and C57BL/6 mice were used as recipients. Experiments involving the transplantation of leukaemic cells were performed in C57BL/6 recipient mice." From this I deduce that cells have been transplanted into Rag2KO irradiated recipients. Why in Rag2KO mice and not in normal C57BL/6 irradiated mice? The Authors do not comment on the reason why they need to use Rag2KO immunocompromised mice and in the description of results do not mention in which recipient mice the results have been obtained.

- This raises more questions:

Are EGFP expressing cells in syngeneic C57Bl/6 mice somehow counter selected and engraftment reduced? May engraftment problems in part explain why the % of EGFP+ cells in the different compartments is so low?

Moreover, from those two sentences it is not clear if the leukemic bone marrow cells or specific subpopulations are transplanted into either Rag2KO mice or C57BL/6 only or into both. In any case, the recipients are never mentioned in the figure legends or described in the results making comprehension harder.

- Fig. 1C: The Authors quantify the amount of AE mRNA expressed in the whole BM and BM subpopulations of compound R26/AE mice under DOX treatment and compared it to the human AML derived cell line Kasumi, which uniformly express AML1-ETO by RT-PCR.

The Authors conclude that the level of expression in these cells is similar to Kasumi and therefore this model system, with a moderate expression of AE, closely resembles the human situation. I do not believe that this conclusion is correct. In their previous paper, Woertge and colleagues showed that the frequency of EGFP+ expressing cells in the different compartments is quite low. Therefore, comparing the amount of AE mRNA extracted from total BM or hematopoietic subpopulations of R26/AE compound mice induced with DOX, as described in the Quantitative real-time PCR section in the online supplement, doesn't really quantify the level of expression/cell. Instead these data suggest that the level of expression of AE is determined by few cells highly expressing the oncogene.

A more appropriate way to assess the level of expression would be to sort out the EGFP+ cells inside the different populations and measure AE expression in these cells.

On this basis the sentence "is suitable for conditionally activating moderate levels of AE in blood cells" is not, in my opinion, supported by the data.

- Fig. 1D-G: These data are convincing and coherent, suggesting a role as "lineage instructor" for AML1-ETO, which seems to skew the relative percentage of the different subpopulations in favour of the myeloid lineage, in accordance with previous studies. However, the effect of AE expression may be underestimated due to the low engraftment/induction feasible in recipient mice. Also, the Authors do not mention if the spleen is infiltrated by myeloid cells at this preleukemic stage, as the BM analysis would suggest.

- Fig. 2A: The Authors evaluate the quantity of HSC and MPP in mice treated or not with DOX. In the corresponding section of results they comment: "Expression of AE for more than six months did not significantly change HSC or MPP populations but revealed a trend towards elevated numbers of these cells (Figure 2A)." However, if the differences are not significant the numbers must be considered equal, therefore no trend can be evident. The fact that the differences are not significant may be due once again to the scarce percentage of AE expressing cells in the HSC compartment. Therefore it is difficult to definitely conclude that AE expression does not alter HSC or MPP frequency.

Notably, HSC/MPP expansion does not seem to be required for the acquisition of further oncogenic mutations.

© EMBO

- Fig. 3: The Authors show the infiltration of several organs with blasts and the appearance of the same cells in the peripheral blood in transplanted mice after a very long latency (15-20 months). However:

i. No lethality data are reported or mentioned. A Kaplan-Mayer would be helpful to judge the penetrance and time to death of those mice. I also believe it is critical to know if the mice die of leukaemia or not.
ii. Besides, a detailed immunophenotyping of the leukaemias is very important to establish how consistent are the different tumours among each other and if they are similar to the human t(8;21) neoplasm.
iii. Moreover, AML1-ETO expression in full blown leukaemia has not been assessed or shown.

- Fig. 4: The Authors examine the number of HSC and hematopoietic progenitors in diseased mice. I do not understand the rationale of testing the number of stem cells in mice with full blown leukemia. However, the observation that no differences in the frequency of stem cells is reported is quite peculiar.
It is well known that AML first engrafts in the bone marrow where malignant cells outcompete normal stem cells; this results in normal hematopoiesis being severely affected. On this premise it is quite unusual that the % of HSC or MPP is unchanged in mice in such critical conditions.

- Fig. 5: The Authors try to revert the leukemic phenotype by an acute ablation of AE function during overt leukaemia. They show that some of the mice which were changed to a DOX-free diet 5 months after transplantation (indicated with blue dots in figures 5B), when analysed, presented normal spleen size, reduced blasts and increased numbers of matured Gr1+ BM granulocytes, directly indicating a reversion of the malignant phenotype. In the same experiment four mice in the DOX-free group instead progressed with leukemia (Orange dots). In my opinion there are several issues here:

i. Leukemic BM cells were injected together with supportive BM into lethally irradiated recipients and AE expression induction was continued for an additional five months then, seven out of ten animals were switched to a DOX-free diet. Four months later all animals were analysed. However, the Authors are not showing a control group of secondary recipients kept on DOX-free diet after transplantation.
ii. Also, the Authors do not show AE expression levels in the mice. As regards those leukaemias not responding to DOX withdrawal (orange dots, Fig. 5B-D), the Authors in the discussion refer to the work of Anders et al 2011 for possible explanation for this observation. However, assessing AE expression, specifically, in all the leukemias would lead to better understanding of the correlation between oncogene ablation and the observed phenotype. I would like to suggest to use the Ly5.2 / Ly5.1 congenic system to perform this experiment in not irradiated recipients. In particular, it would be nice to compare the level of expression of AE in: CD45.2 EGFP+ leukemic cells (DOX+), CD45.2 leukemic cells (DOX+) and CD45.2 leukemic cells (DOX-; responding and not responding leukaemias).
iii. It would be interesting to perform further serial transplantation experiments, especially for the AE non-dependent leukaemias (orange dots in Fig. 5B), to demonstrate that the driving oncogene is not AE (the experiment should be performed in a DOX-free diet scheme).
iv. Finally, in this context, the reported latency of disease assessed by the appearance of circulating blasts is about 5 months, which is a very long time for cells that are already leukemic when transplanted into an irradiated recipient. It is also peculiar that mice that are continuously maintained on the DOX diet do not die even 9 months after transplantation. The characterization of the leukaemias into not irradiated C57BL/6 recipients and a relative Kaplan-Mayer curve assessing the lethality of those tumours would clear most doubts.

- Fig. 6 the Authors wished to establish whether only cells expressing HSC markers or also cells with a more lineage-restricted immune phenotype can act as LSC. To this end, they tested the potential of leukemic HSC (L-HSC defined as L-K+S+CD150+) and leukemic GMP (L-GMP defined as L-K+S-IL7R -CD34+Fc RII/III+) to propagate the disease. Their results show that both subpopulations can recapitulate the hallmarks of the disease. However, also in this case lethality is not reported.
The Authors should state in the text whether they performed the experiment in the presence of DOX induction (likely) or not. It would be interesting to examine whether AE is able to induce SC

competence in GMP cells. To address this question the Authors can:
i. Sort CMP, MEP and GMP from normal ROSA26-iM2-GFP/TgPtet-AML1-ETO (R26/AE) mice, transplant (separately) the cells and induce AE expression by doxycycline from the transplantation point onward.
ii. Collect preleukemic CMP, MEP and GMP from DOX induced R26/AE mice and transplant (separately) the cells into recipient mice.
iii. In order to distinguish whether AE expression itself or further acquired mutations are responsible for the LSC properties of the L-HSC and L-GMP populations, the Authors should repeat the experiment using as secondary recipients also animals fed on DOX-free diet.

- In Figs.7 and 8 the Authors show the results of RNA Sequencing obtained from GMP purified cells derived from R26/AE DOX- and R26/AE DOX+ induced for 10 days and from overtly leukemic animals.
These experiments are interesting and draw correlations between the human disease and the AE deregulated gene expression in mouse GMPs.
The Authors mention in the methods that "experiments involving the transplantation of leukemic cells were performed in C57BL/6 recipient mice" and "For analysing pre-leukemic phenotypes Rag2-/- mice were used as recipients". In the supplementary materials at the RNA Seq section they write: "GMPs from non-induced R26/AE control, transplanted ST AE expressing (10 days DOX) and recipient mice that were transplanted with leukemic BM and permanently exposed to DOX were sorted and the efficiencies confirmed by re-analysing of each sample."
From this description my understanding of the experiment is that Ctrl-GMPs have been isolated from compound mice not induced, ST-GMPs have been isolated from RAG2KO transplanted mice shortly induced with DOX (10 days) and L-GMPs were isolated from transplanted C57BL/6 permanently exposed to DOX. If this is the case too many variables are out of control in this experiment.

Minor points

- In table 1 the Authors show the IPA predicted outcome on several functions, based on the direction of the regulated genes in L-GMPs.
I am not aware of any bioinformatics tool able to reliably predict the real outcome of a transcriptional network, and from what I understand (but I may be wrong) the IPA software only counts how many genes are consistent with a certain prediction as opposed to the ones counteracting it. If this is the case, I think it is over optimistic to infer the resulting function from a simple transcriptional profiling.
If the reported regulations is proven true in independent experiments, the significant enrichment of regulated genes in the identified categories will surely indicate targets in tumour supporting pathways which may be exploited therapeutically.

- Fig. 2E: Match description and figure nomenclature

- Fig. 4C: The percentages in the diagrams on the left do not correspond to those in the graph on the right (they appear inverted)

- Fig. 5: Ablation of AE function in leukaemic mice, legend title not in bold; Fig. 5B: it would be better to perform the statistical analysis on the three groups separately, in order to show that the leukemic phenotype is rescued in animals changed to the DOX-free diet.
Fig. 5D: only two samples; the Authors should state why there are only two dots and which datum is missing (blue dot group); Fig.5F: please indicate the % of immature and mature Gr1 cells.

- Fig. 6F: please indicate the % of GFP+ and GFP- cells

Referee #2 (Remarks):

Cabezas-Wallescheidat al. developed a mouse model conditionally and reversibly expressing AML1/ETO (AE) fusion product in hematopoietic cells following doxocyclin treatment. In this

mouse model they studied the role of AE in AML pathogenesis and the consequences of its functional inactivation. They provided evidence that the expression of AE for more than six months altered the lineage potential of HSC by increasing the mature myeloid cells, but not mature erythroid or lymphoid cells. Moreover, they show that both leukemic hematopoietic stem cells and leukemic granulo-monocytic progenitors are able to propagate the disease if transplanted in irradiated recipients. By ablation of AE following acute DOXO removal, they report a regression of the malignant phenotype. From these data and preliminary observations obtained by whole transcriptome RNA-Seq analysis of murine GMPs, the authors concluded that AE drives major transcriptome changes. This then induces a transcriptional fine tuning which is at the basis of leukemic transformation.
Whereas the reported mouse model and findings are of interest in improving our understanding of AE leukemia pathogenesis, technical and biological issues mean that the authors' conclusions are not fully supported by the data shown. I have mentioned below some of my specific comments and concerns.

Major points:
1. The entire manuscript lacks fundamental information regarding the time-scale when different analyses were performed, the percentage of leukemic cells in the bone marrow and peripheral blood at different stages of the disease, latencies of primary and secondary leukemias and survival curves of primary and secondary transplanted recipients. These data are important for the comprehension of the functional and biological relevance of conditional expression of AE in respect to previously reported AE mouse models.
Moreover, for a correct interpretation of the results, a better definition of the HSC populations used throughout the study should be provided. Why was HSC and MPP phenotypic analysis carried out using different combination of antibodies (see for example Figure 2A and 4A)?.
2. In figure 1 and 2 the authors report that eight months of "moderate" AE expression in HSC and MPP did not result in the expansion of HSC and MPP. However, in the BM they detected an increased number of immature erythroid cell, mature granulocytes and megakaryocytes and reduced T and B cells. It is not clear whether the "moderate" content of AE is referred to the AE mRNA expression levels or frequency of GFP+ BM cells (Figure 1B and C). This should be clarified in the text and AE expression data should be provided also for HCS and MPP populations. Moreover, the time when these analyses were performed is relevant information that should be included in the text and figure legend.
3. In Figure 3 some important controls are missing. These include: i) the percentage of blasts in peripheral blood and bone marrow; ii) H&E stained bone marrow sections; iii) immunohistochemical analysis to address the origin of cells infiltrating different organs. Moreover, the results obtained in age-matched-DOX transplanted mice should be shown.
4. In Figure 4 C (right panel) the labeling of the samples appears incorrect according to the data presented in Figure 4 C left panel. Please check it.
5. Figure 5B shows that DOXO removal is followed by a regression of AE leukemia in 3/7 mice. The lack of regression in these mice is an interesting finding that the authors should investigate, or at least discuss further in the text. Do the AE expression levels vary between recovered and non-recovered leukemias? Are any major chromosomal alterations or leukemias-associated mutations present in these blasts?
6. In my opinion, the results of transcriptome changes and bioinformatics analyses provided in Figure 7 and 8 are very preliminary and, as presented, not informative for the present study. In the absence of a correct validation of the profiles obtained, these data should be shown as supplementary material or removed from the manuscript.
7. Since leukemic mechanisms and transcriptional networks are not reported in this study, the manuscript title should be changed accordingly.

Minor points:
1. It would be helpuful to add flow cytometric analysis plots for all the experiments where only the bar graphs are present (e.g. in Figure 1G) even if as supplementary information. In general, the FACS plots and graphs reported in the figures should be presented more clearly and more consistently labeled .
2. Statistics should be included for the data shown in Figure 5F.
3. In the legend of Figure 6 the H panel is incorrect. It should be G panel

Referee #3 (Comments on Novelty/Model System):

In this manuscript by Cabezas-Wallscheid et al.(EMM-2013-02661), the authors try to understand the effect of AML1-ETO (AE) fusion protein in the pathogenesis of core binding factor acute myeloid leukemias. Taking advantage of a new tetracycline inducible mouse model and state of the art approaches, such as whole transcriptome sequencing, the authors found that AE is able to induce, per se, leukemia in experimental animals skewing the hematopoietic differentiation program towards a myeloid lineage. Noteworthy, the leukemic status undergoes regression upon doxycycline withdrawal and oncogene exhaustion. The authors demonstrate, through isolation and transplantation of different subpopulation of leukemic cells, that tumor initiating cells are not homogeneous and that both hematopoietic stem cells and granulocyte macrophage progenitors are able to reproduce the disease in secondary recipients. RNA-sequencing of cells isolated from the bone marrow of normal, pre-leukemic and leukemic mice revealed that two different "waves of transcriptional rewiring" take place during disease progression: the first, and major one, is the direct effect of the leukemic fusion protein on the hematopoietic gene programs. The second, and a late event, is linked to the transition from pre-leukemic phase to manifest leukemia. Integrating these data with pre-existing public human leukemic database, the authors found a set of 17 genes specifically induced by AE in mouse cells and up-regulated in human and mouse leukemias (but not expressed in normal human tissues) which they propose as could serve as potential new targets for CBF-AMLs upon further investigation and validation.
This study provides additional insights for the role AE plays in the development of acute myelogenous leukemia and certainly describes for the first time a mouse model in which the fusion protein exerts a detectable oncogenic function. However, there are gaps and scientific flaws throughout the manuscript that need to be addressed before further consideration for publication.

Major concerns:

-The authors use a mouse model for conditional gene activation, generated and previously characterized by the same authors, based on tetracycline-regulated transcription activator iM2. Wortge et al. have previously demonstrated that even if the iM2 activator is under the control of ROSA26 endogenous promoter, the transgenic mouse expresses a mosaic pattern of gene activation in the peripheral tissues. Even, within the same tissue/system, as the hematopoietic cells, there is differential expression of the reporter gene among the different subpopulations. Although this pattern of expression could be interesting in an oncogenic setting, since the oncogene is not expressed ubiquitously in all the cells, this could be detrimental here where the authors want to compare the effect of AE on different subpopulations.
In addition to the percentage of GFP-positive cells, the authors should plot the mean of the fluorescence of the positive cells in Figure 1b, . Since the authors assume that the expression of the reporter is proportional to AE expression, the mean of fluorescence of positive cells is more informative than the percentage.

How did the authors perform the experiment in figure 1c? Was the evaluation of AE transcript done in cells isolated from total bone marrow or only in the GFP positive fraction? The authors must provide a detailed description of this experiment in the text, as this represents the most important caveat. Indeed, it is unclear whether they have evaluated the expression of AE transcript irrespective of GFP positivity. The different levels of AE expression could account for the effects the authors describe in the different cell subpopulations. In other words, is the lack of differences in HSC and MPP -due to the low expression of AE in these subpopulations?
In Figure 2a, in the same vein, would the results be different if the authors pre-gate HSC and MPP of induced mice in the GFP positive fraction? If the mice are mosaics after induction,, why do the authors plot all the results as a percentage of total bone marrow instead of pre-gating the subpopulations on GFP positive fraction? Doesn't gating total bone marrow underestimate the results?

-Another important point is that the authors do not adhere to the Bethesda proposals for classification of non-lymphoid hematopoietic neoplasms in mice (Kogan et al. Blood 2002) when characterizing their model. While the authors clearly show infiltration of different organs as well as splenomegaly, they do not show either peripheral blood counts or a Kaplan-Mayer survival curve. The small differences in granulocyte phenotype in the peripheral blood (Figure 3A) look like a pre-

leukemic phase rather than an overt leukemia driven by AE. Usually, the bone marrow architecture of acute leukemic mice is completely subverted and alterations are clearly appreciable in flow-cytometry using forward and side scatters. Similarly, the lack of differences in HSC, MPP, CLP (Figure 4A-B) and mild differences in GMP, MEP, CMP (Figure 4C) are more compatible with a pre-leukemic phase rather than an overt leukemic status. The same is suggested by the long survival of secondary recipients upon transplantation of fully transformed leukemic cells (Figure 5b). Again, the same concern arises from Figure 6F, why should leukemic stem cells give rise to fully differentiated granulocytes (CD11b/Gr1 double positive)?
The authors should be more careful in their conclusions. Collectively, the data presented by the authors suggests that AE expression in this experimental setting induces an indolent myelo-proliferative disorder rather than a fully leukemic disorder such as CBF-AMLs. If the authors do not have further evidence to support their conclusions, they should review their manuscript and conclusions accordingly.

-The authors should state in the text how many L-HSCs and L-GMPs they transplanted in their experiment and how many mice they used to isolate leukemic cells. (Figure 6). Leukemic stem cells are usually assayed using a limiting dilution or in serial transplantation assay, however, engraftment of transplanted cells does not automatically imply long-term self-renewal. Transplanting too many cells could be misleading in assessing their tumorigenic potential.

-The authors identify a set of 17 genes specifically up-regulated during progression and increased also in human samples. Did the authors validate these genes by QPCR in leukemic and normal samples? Does the Figure 8C represent an array or a QPCR? The authors should describe the experiment better in the figure legend.

-The authors describe a late deregulation of "cancer related pathways" during the progression to leukemia. Since these changes are not a direct effect of AE, the authors should discuss the secondary mutations that could be responsible for this secondary event.

Minor points:

-Figure 1F, 1G. Use relative and not absolute numbers for y axis and then be consistent through the figures (normalizing each column to its control, plotting non-induced as 100%, will improve visualization of small differences).
-Figure 1G. Specify in figure legend the number of mice used in the experiment.
-Figure 2C. Specify in figure legend how long the authors induced the mice.
-Does Figure3A refer to PB or BM?
-Figure 4A-B. Are gating strategies made using control or induced mice?
-Figure 4C. The authors should report the number of mice.
- Figure 5A-D. The authors should report the number of mice.
- Figure 6D-G. The authors should report the number of mice.
-Does Figure 8D refer to BM or PB?

1st Revision - authors' response 02 July 2013

Response letter to the referees
We very much liked the answers of our referees and think that all three made very fair, informed and helpful suggestions. Building on these comments, we have now substantially modified the manuscript and added new data. We also very much appreciate the effort and time taken by each of the tree referees and hope that the changes we have introduced to the present manuscript will satisfy most requests made.

Answer to Referee 1
Referee 1 (Comments on Novelty/Model System):

Comment: The proposed model seems promising but in my opinion there are still a few issues that need to be addressed (see detailed review)

Referee 1 (Remarks):
In this study the Authors examine the effect of the conditional expression of a TET inducible version of the human oncogene AML1-ETO in the development of AML and how its acute withdrawal affects the course of disease. The general strategy is thought through and tries to answer important questions that are central to the cancer biology field.
The Authors took advance of a previously established transgenic mouse (Rhoades et al) and crossed it with a GFP coupled TET inducible system (Woertge et al) which allows both to express the oncogene and to monitor the level of induction mediated by doxycicline administration.

Few comments on the model system.
In principle and overall, the usage of TET inducible systems in vitro allows the fine tuning of gene expression, and facilitate the switching on / off of a gene of interest to assess the effects of acute up / down gene regulation. However, the use of these systems in vivo poses great problems in terms of bioavailability and clearance of the inducer over time, and, differently from the in vitro setting, there is a limit in the number of parameters one can change to comply with the experimental requirements. A previous publication (Wortge et al.) from the same group established and described the ROSA26-iM2-GFP reporter background strain, showing that doxycycline mediated level of AE induction was limited and it varied in the different hematopoietic compartments. This premise is important because some of the conclusions the Authors came up with may be somehow affected by this intrinsic heterogeneity.

We were very pleased that Referee1 thought that our approach is in principle thought through and try to answer important questions that are central to the cancer biology field.
We completely agree with the referee that the use of the Tet inducible system is very appropriate for tissue culture experiments because of the possibilities to change conditional transgene expression levels at a single cell resolution. In addition to tissue culture experiments and since the first publication of Tet-inducible mice from the Bujard laboratory, the Tet-system has also gained more and more importance for studying the effects of conditional activation/extinction of gene function in mice and is now generally accepted in the field. I also agree with Referee 1 that a general concern put forward by many investigators had been the bioavailability, pharmacodynamics and –kinetics of DOX in living animals. However, in pervious experiments we and many other groups have shown that DOX has a good biavailability profile in mice which also includes the brain, often thought to be a difficult organ to reach (Mack et al. 2001, Science 292, 2501-4, Wörtge et al. 2010, BMC Dev Biol. 10, 95, Chen et al. 2013, Mol Ther Nucleic Acids., 2:e85). Careful analysis of many mouse models by us and others furthermore demonstrated that the DOX on/off system is very convenient for conditionally regulating gene expression in adult blood cells (Bockamp et al. 2006, Blood 108,1533-41, Kim et al. 2009, Blood 113, 1086-96) and also for regulating gene expression in HSC and blood cell progenitors (Wilson et al. 2008, Cell 135, 1118-29). Tet-dependent conditional gene activation was furthermore successfully used in blood cell lineage marking and tracing experiments, demonstrating that DOX on/off induction kinetics can be tightly controlled and showing excellent DOX bioavailanility in the developing embryo (Bockamp et al. 2009, Mech Dev.126 (10):863-72). For these reason, we do not think that bioavailability, pharmacokinetics or -dynamics of DOX poses a real problem in our setup. Moreover, we would argue that the mosaic expression pattern in the R26/AE transplantation model is caused by the specific transcriptional activity of the ROSA26 knock-in locus that was used to drive conditional transgene expression. This is directly supported by previous work from our laboratory (Wörtge et al. 2010, BMC Dev Biol. 10, 95 and Hameyer et al. 2007, Physiol Genomics 19 31, 32-41) and also in line with the recent publication by Takiguchi and colleagues (Takiguchi et al. 2013, PLoS One 8, e54009) demonstrating mosaic expression in blood cells from the endogenous ROSA26 gene locus. Finally, we would argue that pan-haematopoietic mosaic expression of transgenes is very well suited for studying the effect of leukaemia-associated gene products in blood cells since it more adequately recapitulates the situation found in patients where the leukaemic clone is initially surrounded by an environment of mostly non-leukaemic cells.

General concerns of Referee 1

1) No lethality data nor detailed immunophenotyping of the leukaemias are shown in order to establish how consistent are the different tumours among each other and if they mimic the human t(8;21) hallmarks.

Referee 1 remarks that no lethality data nor detailed immunophenotyping of the leukaemias are shown in order to establish how consistent are the different tumours among each other and if they mimic the human t(8;21) hallmarks. Referee 1 is absolutely right in that we did now show survival curves for our mice. As a matter of fact our mice do not die even after very long induction periods of 20-22 months. This result is also completely in line with previous findings about t(8;21) AML mouse models reporting in all cases the complete lack of lethality following AE-activation. The fact that in humans the evolution from the initial t(8;21) translocation to overt AML might take many years (for example the nice paper by Wiemels et al. 2002, Blood 99, 3801-3805) also supports the view that in mice -without using additional mutagenic substances- it might just not be possible to recapitulate the complete natural history of AML till death. Since in the current paper we have already chosen very long experimental time frames (being also much more extended than in previous studies) without seeing lethality, we think that it is not mandatory to show survival curves. To also state that we do not exactly recapitulate the situation found in human t(8;21) CBF AML, we have now changed the title from “Leukemogenic mechanisms, cancer stem cell hierarchies and transcriptional networks promoting core binding factor acute myeloid leukaemia” to “Instruction of haematopoietic lineage choices, evolution of transcriptional landscapes and cancer stem cell hierarchies derived from an AML1-ETO mouse model”. In the updated manuscript we now explicitly highlight the fact that our mice did not die and provide two possible explanations (AE expression in only a subset of cells and/or too long experimental time needed to recapitulate the natural history of CBF AML to reach the end point lethality) in the Discussion on page 19.
With regard to the immunophenotyping Referee 1 is absolutely correct that we do not show immunophenotyping data. Regarding this, I have asked the pathologist (Tony Lehr), who did the sections. Unfortunately, Prof. Lehr has recently moved from the University of Lausanne to start a private enterprise in Germany and he does not conserve any sections for doing these additional experiments. Although, it would be excellent and very complementary to the FACS data shown, we cannot make additional stainings for the current paper. However, we think that enclosing pictures showing organ infiltration will be necessary for describing the phenotype adequately as the invasion of cells into non-haematopoietic organs is one important criteria for classifying nonlymphoid neoplasms as leukaemias (Kogan et al. 2002, Blood 100, 238-245).
In addition, we would argue that our FACS data unambiguously demonstrate the specific amplification of CD11b/Gr1 myeloblasts (in the BM of leukaemic mice (Figure 3A, Supplementary Figure 5B) and also the BM and spleens of mice that have been DOX-induced for more than six months (Figure 1F and Supplementary Figure 4)). Which cells other than the increased myeloblasts or derivatives thereof will be candidates to invade non-haematopoietic organs? Directly supporting this argument is also the fact that we found in all analysed mice only blasts but no other leukaemic blood cells and that these blasts had the typical myeoloblast morphology (for example compare blasts shown in Figure 3B of our paper with blasts in Figure 3 of the paper by Yuan et al. 2001, Proc Natl Acad Sci U S A 98, 10398-403). Since circulating blasts will reach other organs via the blood stream, it is to be expected that these cells will produce the infiltrating masses seen in kidney, liver and lung. For all the above reasons, and since we did not detect similar infiltrates in non-induced control recipients, we think that it would be very good but not absolutely required to show additional stains.

2) AML1-ETO expression in ful- blown leukaemia is not shown.

Referee 1 infers that we did not show AE expression in full-blown leukaemia. It is true that we did not analyse protein expression in the current paper. Previous experiments from the Zhang/Tenen laboratory investigated AE protein production with the here used TgPtet-AML1-ETO mouse model by Western blotting. These experiments demonstrated that an activated Ptet-AE switch will result in AE protein expression (Figure 4 in Rhoades et al. 2000, Blood 96, 2108-2115). It is also true that we did not explicitly investigate in the current paper the question, if leukamic cells produce AE protein and did not provide this information in the text. However, our RNA-Seq analysis (Supplementary Table 1 position 2454, 2455 and 7313, Ref-Seq numbers NM_001111026, NM_001111027 and NM_009822) proofs that L-GMP cells actively transcribed AE mRNA (normalized RPKM value 5,86). RNA-Seq analysis thus demonstrates that AE-specific mRNA is present in leukaemic blasts.

3) The Authors use EGFP positivity as a quantitative readout of engraftment but, as said above, this is not a reliable readout for it. In their case, it would be much better to use the ly5.1 / ly5.2 syngeneic mouse model for adoptive BM transplant.

Referee 1 states that we used GFP positivity as a quantitative readout of engraftment and points out that, this is not a reliable readout for it. He/she further suggests that, it would be much better to use the ly5.1 / ly5.2 syngenic mouse model for adoptive BM transplant.
The referee is absolutely right that the use of the ly5.1 / ly5.2 system would have been a great strategy for discriminating transplanted from endogenous blood cells. Before starting our experiments we have thought about this possibility and performed also first FACS analysis experiments of peripheral blood samples using CD45.1/CD45.2 antibodies. However, we did decide against this system, as it would have meant to set up much more crosses and to wait for even more time for the results. Also for the questions we wanted to ask, we did not see an urgent need to use the ly5.1 / ly5.2 approach.
To clarify matters and to make clear that we did not use GFP as a means for quantifying engraftment, we have now completely changed the first section of Results (page 6 and 7 of the current manuscript). We explain that we use GFP only as a marker for transgene activation and evaluated the percentage of GFP+ cells in different blood cell lineage. In the updated version of the paper we now provide a new figure presenting EGFP+ and EGFP- cell populations in DOX-induced, reconstituted recipients (Supplementary Figure 1). Moreover, taking into account that reconstituted mice were lethally irradiated in all experiments, it is to be expected that HSC, most BM progenitors and short lived blood cells will have disappeared at seven weeks following reconstitution, when we started the DOX-induction experiments. The changes introduced in the updated manuscript will make clear to the reader that any conclusions reached are not based on GFP expression as a measure for reconstitution but that GFP-expression was simply used to identify cells with an activated DOX-switch. In line with this we now report that the observed lineage skewing phenotypes (for example in the expansion of GMP pools) were driven by the GFP-expressing subpopulation (Figure 4E and F, Supplementary Figure 10, Supplementary Figure 15).
In the experimental series about LSC we have also applied GFP expression as an experimental tool. Here we used GFP expression for evaluating the lineage potential of transplanted L-HSC and L-GMP (Supplementary Figure 17). Since the conditions we have used revealed a clear phenotype, we think that it is not absolutely necessary to show engraftment using syngenic CD45.1/CD45.2 markers. Finally, to repeat the in the paper reported *in vivo* experiments with CD45.1/CD45.2 blood cell markers is not a straightforward task. These experiments will at least require 2-3 years of additional work. Most importantly, although the use of CD45.1/CD45.2 would have been a very smart strategy, we cannot see any very burning questions that have to be answered in the context of the current experimental readouts and thus justify this considerable effort.

Specific points Referee 1
Fig. 1B: The Authors examine the % of EGFP+ cells in the different subcellular compartments in transplanted mice kept under DOX treatment. Which is the real % of engraftment in those mice? Is the percentage of EGFP+ cells similar to the expected one or not?

Referee 1 wonders about the engraftment of cells in the recipients and asks what would be the expected percentage of GFP+ cells in reconstituted mice. As already discussed above, the engraftment of the haematopoietic compartment following lethal irradiation of the recipients should be near to 100%. To test the optimal radiation dose before performing the actual reconstitution experiments, we initially did experiments with increasing radiation regimes. The radiation doses used in our experiments did induce lethality in all not BM-reconstituted animals we have tested. However, we agree that some more long-lived cells like central or peripheral memory B- and T-cells or peripheral patrolling APC will surely survive the radiation and stay on in reconstituted mice. To make it clear that we do not use GFP expression in reconstituted mice as a measure for reconstitution, we have completely re-written the first section in Results (see above).
We would like to stress that the focus of the manuscript and the questions asked do not depend on reconstitution efficiencies. On the contrary, the advantage of mosaic transgene expression is that we have not a sudden and complete activation profile in all blood cells (which is a non-physiological situation and does not reflect the initially limited expression of AE in CBF AML patients) but that we activate transgene expression only in a subset of cells in each lineage. As said above, when we looked for GFP+ and GFP- cells in DOX-induced mice, we found that the phenotype was sustained in each case by the GFP-expressing population.

The methods for this experiment and others are not clearly written. From the BM transplantation section: "For analysing pre-leukaemic phenotypes RAG2-/- mice and for leukaemic phenotypes RAG2-/- and C57BL/6 mice were used as recipients. Experiments involving the transplantation of

leukaemic cells were performed in C57BL/6 recipient mice." From this I deduce that cells have been transplanted into Rag2KO irradiated recipients. Why in Rag2KO mice and not in normal C57BL/6 irradiated mice? The Authors do not comment on the reason why they need to use Rag2KO immunocompromised mice and in the description of results do not mention in which recipient mice the results have been obtained.

In the current paper we now have substantially expanded Materials and Methods. As requested, in the modified manuscript we describe the background of recipient mice, DOX-induction times and the number of animals tested together with the statistics for each experiment (Materials and Methods and in the accompanying the Supplementary Figures). With regard to the question why we used at the beginning RAG2-deficient recipients we can say the following: In the beginning of the experiments, we had no obvious reasons for choosing $RAG2^{-/-}$ of B6 background animals since our bi-transgenic mice were on a pure B6 background. However, we felt that since $RAG2^{-/-}$ mice lack T and B cells, we might get a clearer picture about possible effects of AE expression in these lineages. For the remaining experiments we used mostly B6 mice because in our animal facility the price for a B6 mouse is lower than for a RAG-deficient animal. Finally and most importantly, when we used RAG2-deficient and B6 recipients alongside, very similar results were obtained.

This raises more questions: Are EGFP expressing cells in syngeneic C57Bl/6 mice somehow counter selected and engraftment reduced? May engraftment problems in part explain why the % of EGFP+ cells in the different compartments is so low? Moreover, from those two sentences it is not clear if the leukemic bone marrow cells or specific subpopulations are transplanted into either Rag2KO mice or C57BL/6 only or into both. In any case, the recipients are never mentioned in the figure legends or described in the results making comprehension harder.

To our knowledge there is no report in the literature for counter selection in syngenic animals and in RAG-deficient syngenic animals. However, it can happen that when female BM is transferred into male recipients and vice versa that there is a very mild counter selection but no rejection or graft versus host reaction. In none of our experiments we did perform cross gender transplants. For this reason, we would argue that engraftment problems can be excluded. With the exception of the leukemic stem cell experiments, where we used highly FACS purified GMP and HSC cells together with supportive BM, we have used whole BM cells in all other transfer experiments. On page 27 of Supplementary Information in Materials and Methods we provide a detailed description of the experimental details that have been used for the adoptive transfer experiment with L-HSC and L-GMP subpopulations including cell numbers, number of supportive cells, background of supportive cells and radiation strength. This information will make it clear how we performed the experiment.

Fig. 1C: The Authors quantify the amount of AE mRNA expressed in the whole BM and BM subpopulations of compound R26/AE mice under DOX treatment and compared it to the human AML derived cell line Kasumi, which uniformly express AML1-ETO by RT-PCR.
The Authors conclude that the level of expression in these cells is similar to Kasumi and therefore this model system, with a moderate expression of AE, closely resembles the human situation. I do not believe that this conclusion is correct. In their previous paper, Woertge and colleagues showed that the frequency of EGFP+ expressing cells in the different compartments is quite low. Therefore, comparing the amount of AE mRNA extracted from total BM or hematopoietic subpopulations of R26/AE compound mice induced with DOX, as described in the Quantitative real-time PCR section in the online supplement, doesn't really quantify the level of expression/cell. Instead these data suggest that the level of expression of AE is determined by few cells highly expressing the oncogene. A more appropriate way to assess the level of expression would be to sort out the EGFP+ cells inside the different populations and measure AE expression in these cells. On this basis the sentence "is suitable for conditionally activating moderate levels of AE in blood cells" is not, in my opinion, supported by the data.

The point made by the referee is absolutely right and we completely agree that we should not conclude from our data that the level of expression in these cells is similar to Kasumi and therefore this model system, with a moderate expression of AE, closely resembles the human situation. In the current version of the paper we have taken out this statement. Instead we now state in the first section of the Results on page 6-7 in essence that (i) the ROSA26/M2 effector directed conditional mosaic GFP transgene expression to LT-ST-HSC and different progenitors (ii) that AE-specific

mRNA was detected in all analysed stem, progenitor and adult blood cell populations and (iii) that the system is not leaky. We hope that Referee 1 now is happy with our conclusions.

Fig. 1D-G: These data are convincing and coherent, suggesting a role as "lineage instructor" for AML1-ETO, which seems to skew the relative percentage of the different subpopulations in favour of the myeloid lineage, in accordance with previous studies. However, the effect of AE expression may be underestimated due to the low engraftment/induction feasible in recipient mice. Also, the Authors do not mention if the spleen is infiltrated by myeloid cells at this preleukemic stage, as the BM analysis would suggest.

It was good to see that Referee 1 thinks that the lineage instruction experiments are convincing. We think that the paper clearly shows at the cellular and developmental level that aberrant AE expression skews normal blood cell development towards myelopoiesis. With regard to the underestimation of the effect, we would like to again put forward the argument that mosaic expression -as seen in our model- is very appropriate to mimic the evolution of the human disease downstream of the initial t(8;21) translocation. With regard to the possible underestimation of AE effects we can say that we see clear lineage instruction effects.
On the topic of spleen infiltration/splenomegaly, we agree with the referee that the paper would greatly benefit from the inclusion of additional data. As requested, the current version of the paper now includes in the first paragraph on page 8 of the Result section a description of the phenotype seen in spleen and we have added in Supplementary Figure 3 representative sections for thymus, lymph nodes and spleen. In addition, we now provide FACS data from control and AE-expressing spleens demonstrating the absolute and relative increase of myeloid and red blood cells in DOX-induced animals (New Supplementary Figure 4).

Fig. 2A: The Authors evaluate the quantity of HSC and MPP in mice treated or not with DOX. In the corresponding section of results they comment: "Expression of AE for more than six months did not significantly change HSC or MPP populations but revealed a trend towards elevated numbers of these cells (Figure 2A)." However, if the differences are not significant the numbers must be considered equal, therefore no trend can be evident. The fact that the differences are not significant may be due once again to the scarce percentage of AE expressing cells in the HSC compartment. Therefore it is difficult to definitely conclude that AE expression does not alter HSC or MPP frequency. Notably, HSC/MPP expansion does not seem to be required for the acquisition of further oncogenic mutations.

As requested by the referee we have taken out the sentence "Expression of AE for more than six months did not significantly change HSC or MPP populations but revealed a trend towards elevated numbers of these cells" which is now reading "Interestingly, DOX induction for eight to ten months did not significantly change LT- and ST-HSC". In the current manuscript we now also include tables for both the absolute percentages within the whole population of BM cells and the relative percentages within the gated populations for LT- and ST-HSC indicating the number of animals tested in each case, the mean percentage, the standard deviation, the standard error of mean and the resulting p-values. Importantly, we show that there is no statistically significant change within the population of GFP+ LT- and ST-HSC and CLP (Supplementary Figure 9) but yes that GFP+ GMP and MEP change (Supplementary Figure 10) whereas all GFP- stem and progenitor populations remain similar (Supplementary Figure 9 and 10). This clearly demonstrates that in absolute and relative terms AE expression does not significantly alter the HSC pool but specifically increases the GMP population.

Fig. 3: The Authors show the infiltration of several organs with blasts and the appearance of the same cells in the peripheral blood in transplanted mice after a very long latency (15-20 months). However: i. No lethality data are reported or mentioned. A Kaplan-Mayer would be helpful to judge the penetrance and time to death of those mice. I also believe it is critical to know if the mice die of leukaemia or not. ii. Besides, a detailed immunophenotyping of the leukaemias is very important to establish how consistent are the different tumours among each other and if they are similar to the human t(8;21) neoplasm. iii. Moreover, AML1-ETO expression in full blown leukaemia has not been assessed or shown.

To answer the above comments, we would like to refer to our response given on page 2 (General Concerns (subsection 1).

The Authors examine the number of HSC and hematopoietic progenitors in diseased mice. I do not understand the rationale of testing the number of stem cells in mice with full blown leukemia. However, the observation that no differences in the frequency of stem cells is reported is quite peculiar. It is well known that AML first engrafts in the bone marrow where malignant cells outcompete normal stem cells; this results in normal hematopoiesis being severely affected. On this premise it is quite unusual that the % of HSC or MPP is unchanged in mice in such critical conditions.

The reason why we examined the stem cell compartment in diseased mice was because we wanted to know if HSC or progenitors were expanded. If this would have been the case then therapies that target the expanding population of HSC would be appropriate. However, we did not find an increase in HSC but -as in pre-leukaemic mice- a selective expansion of GMP. Most interestingly, we have now gated on GFP+ versus GFP- cells within the stem cell population. Doing this, we found that two mice had barely any GFP+ cells and that in all other animals the GFP+ HSC were not increased (Figure 4D, Supplementary Figure 14). Although we did not further investigate this finding, it might be possible that two completely different situations take place in individual mice: Namely that in the two mice with no or very few GFP+ HSC the disease might be entirely driven by a more differentiated L-GMP-like LCS population whereas in the other mice L-HSC and/or L-GMP might act as LSC. However, this interesting possibility awaits further investigation. Finally, we now show in the updated manuscript that the apparent amplification of GMP and the reduction of other progenitors were driven by the GFP+ population (newly included FACS data in Figure 4E and F and Supplementary Figures 14 and 15). We think that these findings clearly demonstrate that long-term AE induction leads to an MPD-like myeloid leukaemia phenotype in mice and that the induction of this phenotype is not dependent on and accompanied by the specific expansion of HSC.

Fig. 5: The Authors try to revert the leukemic phenotype by an acute ablation of AE function during overt leukaemia. They show that some of the mice which were changed to a DOX-free diet 5 months after transplantation (indicated with blue dots in figures 5B), when analysed, presented normal spleen size, reduced blasts and increased numbers of matured Gr1+ BM granulocytes, directly indicating a reversion of the malignant phenotype. In the same experiment four mice in the DOX-free group instead progressed with leukemia (Orange dots). In my opinion there are several issues here: i. Leukemic BM cells were injected together with supportive BM into lethally irradiated recipients and AE expression induction was continued for an additional five months then, seven out of ten animals were switched to a DOX-free diet. Four months later all animals were analysed. However, the Authors are not showing a control group of secondary recipients kept on DOX-free diet after transplantation.

Referee 1 is right that we did not have a group of mice were leukaemic BM was immediately put into secondary recipients not exposed to DOX. Proceeding this way would have had the advantage that the leukaemia would not have been progressed that far possibly inducing stronger effects of phenotype reversion following the DOX-switch. We are aware that including this experiment could have answered the question what would be the consequences of AE ablation in a moderately or less progressed MPD-like myeloid leukaemia. However, we found in three mice with long-term leukaemia progression obvious signs of remission and in two mice a very considerable reversion of the phenotype. These results document for the first time in an autochthonous mouse model a benefit of AE-inactivation. We believe that his is an important finding for the field. Since in mice, as it is in patients, the nature and temporal occurrence of secondary molecular events needed for the progression towards manifest disease will vary between individuals and also between single leukaemia initiating clones, the effect of AE reversal is most likely dependent on the nature and timing of these events. For this reason, we believe that analysing the effect of AE ablation in a fairly progressed leukaemic state is even a stronger proof for documenting that AE inactivation can have a positive therapeutic effect. To make the point clear that any benefit resulting from acute AE-activation will substantially depend on the nature of additional secondary mutations, we state in the second paragraph on page 20 in the Discussion that "because secondary mutations necessary for the onset of overt leukaemia are heterogeneous in t(8;21) AML patients (Hatlen et al, 2012), it will be crucial to develop reliable diagnostic markers to identify those patients that will benefit from AE inactivation". In conclusion, we think that it would have been very interesting and a good additional experiment to have included the without DOX group directly after transfer of leukaemic cells as

suggested by Referee 1 but we also think that our experiments provides sufficient proof that acute ablation of AE can provide a therapeutic benefit.

ii. Also, the Authors do not show AE expression levels in the mice. As regards those leukaemias not responding to DOX withdrawal (orange dots, Fig. 5B-D), the Authors in the discussion refer to the work of Anders et al 2011 for possible explanation for this observation. However, assessing AE expression, specifically, in all the leukemias would lead to better understanding of the correlation between oncogene ablation and the observed phenotype. I would like to suggest to use the Ly5.2 / Ly5.1 congenic system to perform this experiment in not irradiated recipients. In particular, it would be nice to compare the level of expression of AE in: CD45.2 EGFP+ leukemic cells (DOX+), CD45.2 leukemic cells (DOX+) and CD45.2 leukemic cells (DOX-; responding and not responding leukemias).

It is true that we are not showing AE expression levels in the current manuscript and that there will be still experiments that can substantially improve the manuscript. Although the experiments suggested by Referee 1 would definitely solve the question if the lack of extinction of transgene expression is the reason for leukaemia progression in some mice, we think that performing these experiments -in addition to the already very extensive data presented- is beyond the scope of the current manuscript. This does not mean that investigating the reasons for leukaemia progression is not important. On the contrary, it will be necessary to conduct these experiments in preclinical mouse models like the one presented here and if possible later on also with patient samples. However, such experiments represent a complete novel research project on its own right.

iii. It would be interesting to perform further serial transplantation experiments, especially for the AE non-dependent leukaemias (orange dots in Fig. 5B), to demonstrate that the driving oncogene is not AE (the experiment should be performed in a DOX-free diet scheme).

Here, we would like to again take up the previous line of arguments. Of course would it be very interesting to do additional transplantations and to see which driver onkogenes/tumour suppressors take command during further steps of disease progression and possibly make AE expression irrelevant. In the current paper we provide for the first time a glimpse into the dynamic evolution of events taking place downstream of the initial AE activation in mice and we did not and cannot intend within the current body of work to give an exhaustive analysis of the mechanisms that are responsible further downstream for making leukemic clones independent of AE.

iv. Finally, in this context, the reported latency of disease assessed by the appearance of circulating blasts is about 5 months, which is a very long time for cells that are already leukemic when transplanted into an irradiated recipient. It is also peculiar that mice that are continuously maintained on the DOX diet do not die even 9 months after transplantation. The characterization of the leukaemias into not irradiated C57BL/6 recipients and a relative Kaplan-Mayer curve assessing the lethality of those tumours would clear most doubts.

To answer the final comment of Referee 1 in the section about acute inactivation of AE, in our model disease progression is a very slow process with very long latency and as a matter of fact, mice did not die even after very long times of DOX-induction. We are aware that one possibility to analyse leukaemic potential *in vivo* is to transfer cells into non-irradiated syngenic animals. However, we did not do this experiment. We think that the presence of general hallmarks characteristic for leukaemia including the consistent occurrence of myeloblastsblasts in the BM and the periphery, extramedullary myelopoisis, anaemia, organ invasion (that are all criteria for defining leukaemia in mice, Kogan et al. 2002, Blood 100, 238-254) and the demonstration that L-GMP acquire unlimited self-renewal and can long-term produce myeloid progeny (a criterion defining cancer stem cells) are sufficient for defining the in our model observed phenotype in the current manuscript as indolent MPD-like myeloid leukaemia.

Fig. 6 the Authors wished to establish whether only cells expressing HSC markers or also cells with a more lineage-restricted immune phenotype can act as LSC. To this end, they tested the potential of leukemic HSC (L-HSC defined as L-K+S+CD150+) and leukemic GMP (L-GMP defined as L-K+S-IL7RaCD34+FcgRII/III+) to propagate the disease. Their results show that both subpopulations can recapitulate the hallmarks of the disease. However, also in this case lethality is not reported. The Authors should state in the text whether they performed the experiment in the presence of DOX

induction (likely) or not. It would be interesting to examine whether AE is able to induce LSC competence in GMP cells. To address this question the Authors can: i. Sort CMP, MEP and GMP from normal ROSA26-iM2-GFP/TgPtet-AML1-ETO (R26/AE) mice, transplant (separately) the cells and induce AE expression by doxycycline from the transplantation point onward. ii. Collect preleukemic CMP, MEP and GMP from DOX induced R26/AE mice and transplant (separately) the cells into recipient mice. iii. In order to distinguish whether AE expression itself or further acquired mutations are responsible for the LSC properties of the L-HSC and L-GMP populations, the Authors should repeat the experiment using as secondary recipients also animals fed on DOX-free diet.

Similar to the autochthonous setting following long-term DOX induction, we are not observing any lethality in L-GMP and L-HSC recipient mice even in very long induction setups of up to 22 month of DOX. As requested, we describe now in Material and Methods clearly that the transfer was done using DOX-exposed recipients (page 7 of Supplementary Information) and to make it more clearly understandable for the reader that DOX-exposed mice were used as recipients, we have now modified Figure 6A accordingly.

In Figs.7 and 8 the Authors show the results of RNA Sequencing obtained from GMP purified cells derived from R26/AE DOX- and R26/AE DOX+ induced for 10 days and from overtly leukemic animals. These experiments are interesting and draw correlations between the human disease and the AE deregulated gene expression in mouse GMPs. The Authors mention in the methods that "experiments involving the transplantation of leukemic cells were performed in C57BL/6 recipient mice" and "For analysing pre-leukemic phenotypes Rag2-/- mice were used as recipients". In the supplementary materials at the RNA Seq section they write: "GMPs from non-induced R26/AE control, transplanted ST AE expressing (10 days DOX) and recipient mice that were transplanted with leukemic BM and permanently exposed to DOX were sorted and the efficiencies confirmed by re-analysing of each sample." From this description my understanding of the experiment is that Ctrl-GMPs have been isolated from compound mice not induced, ST-GMPs have been isolated from RAG2KO transplanted mice shortly induced with DOX (10 days) and L-GMPs were isolated from transplanted C57BL/6 permanently exposed to DOX. If this is the case too many variables are out of control in this experiment.

For this experiment we have only used C57BL/6 animals. In the current manuscript we have clarified this issue and provide a detailed description of how Ctrl-, ST- and L-GMP populations were obtained (Supplementary Information/Materials and Methods page 25).

Minor points

In table 1 the Authors show the IPA predicted outcome on several functions, based on the direction of the regulated genes in L-GMPs. I am not aware of any bioinformatics tool able to reliably predict the real outcome of a transcriptional network, and from what I understand (but I may be wrong) the IPA software only counts how many genes are consistent with a certain prediction as opposed to the ones counteracting it. If this is the case, I think it is over optimistic to infer the resulting function from a simple transcriptional profiling.
If the reported regulations are proven true in independent experiments, the significant enrichment of regulated genes in the identified categories will surely indicate targets in tumour supporting pathways which may be exploited therapeutically.

Review 1 cites that pathway analyses are not predictive tools but rather observations. The reviewer is correct in multiple ways: a) our measurements are observations that reflect a change between two states, and not kinetic studies; b) the observations can be causal or symptomatic; and c) while causal system biology networks do in fact exist, they are very context specific and currently limited in their scope and application. Reflecting this, we have modified the text and highlight that we are assaying a current state, and not causal relationship. For this reason we have removed “promoting” and replace it with “associated with”. In the Results section the modified text on page 16 now reads as follows: As shown in Table 1, IPA revealed a significant up-regulation of cellular pathways associated with transformation, survival, proliferation, RNA expression and cytoplasmic organization that was accompanied by a down-regulation of cell death pathways Equally, we have modified the text in the Discussion on page 21: “Confirming the leukaemogenic nature of secondary events in L-GMP, IPA pathway analysis identified pathways associated with oncogenic transformation and the prevention of apoptosis”. To emphasize that additional independent

experiments are needed and not to give an overoptimistic interpretation of our data we have also added on page 21 to 22 of the Discussion "However, additional transcriptional profiles especially those derived from AML patients during disease remission and recurrence are needed to identify novel functionally relevant targets".

Fig. 2E: Match description and figure nomenclature.

We now have matched the description with the nomenclature in Fig. 2E.

Fig. 4C: The percentages in the diagrams on the left do not correspond to those in the graph on the right (they appear inverted).

In the revised paper we have now changed Figure 4C. We now show the absolute percentages of CMP/GMP/MEP cells within the population of nucleated whole BM cells in Figure 4C. In addition, we have amended the manuscript and Supplementary Figure 8B now presents also representative FACS plots and relative percentages of each progenitor.

Fig. 5: Ablation of AE function in leukaemic mice, legend title not in bold;
The legend title of figure 5 is now in bold.

Fig. 5B: it would be better to perform the statistical analysis on the three groups separately, in order to show that the leukemic phenotype is rescued in animals changed to the DOX-free diet.
As suggested, Figure 5B now shows –DOX not recovered (orange dots) and –DOX recovered (blue dots) separately.

Fig. 5D: only two samples; the Authors should state why there are only two dots and which datum is missing (blue dot group);

Figure 5D now includes the data for all analysed mice and controls.

Fig.5F: please indicate the % of immature and mature Gr1 cells.

For completeness and as requested by the editor and also by the referees, we have included in the updated manuscript representative gating strategies, corresponding FACS plots, absolute percentages of the analysed populations of cells within the BM (or spleen) population of cells, relative percentages of the gated cells and statistical information. As a result of this restructuring in Figure 5F the absolute percentages of control, +DOX, progressed –DOX and reverted –DOX immature and mature granulocytes is shown. Furthermore, we now present a representative FACS histogram (Supplementary Figure 16A), a plot showing relative cell percentages of the gated populations and tables indicating statistical information and means of both absolute and relative percentages (Supplementary Figure 16B).

Fig. 6F: please indicate the % of GFP+ and GFP- cells.

The histogram of former Figure 6F is now shown in Supplementary Figure 16A and contains percentages of GFP+ cells in the table that is shown below the histogram.

Answer to Referee 2

Cabezas-Wallescheidat al. developed a mouse model conditionally and reversibly expressing AML1/ETO (AE) fusion product in hematopoietic cells following doxycycline treatment. In this mouse model they studied the role of AE in AML pathogenesis and the consequences of its functional inactivation. They provided evidence that the expression of AE for more than six months altered the lineage potential of HSC by increasing the mature myeloid cells, but not mature erythroid or lymphoid cells. Moreover, they show that both leukemic hematopoietic stem cells and leukemic granulo-monocytic progenitors are able to propagate the disease if transplanted in irradiated recipients. By ablation of AE following acute DOXO removal, they report a regression of the malignant phenotype. From these data and preliminary observations obtained by whole transcriptome RNA-Seq analysis of murine GMPs, the authors concluded that AE drives major transcriptome changes. This then induces a transcriptional fine tuning which is at the basis of leukemic transformation.

Whereas the reported mouse model and findings are of interest in improving our understanding of AE leukemia pathogenesis, technical and biological issues mean that the authors' conclusions are not fully supported by the data shown. I have mentioned below some of my specific comments and concerns.

It was good to see that Referee 2 thinks that the reported mouse model and findings are of interest in improving our understanding of AE leukemia pathogenesis. His/her suggestions were very reasonable and helpful.
Major points:

1. The entire manuscript lacks fundamental information regarding the time-scale when different analyses were performed, the percentage of leukemic cells in the bone marrow and peripheral blood at different stages of the disease, latencies of primary and secondary leukemias and survival curves of primary and secondary transplanted recipients. These data are important for the comprehension of the functional and biological relevance of conditional expression of AE in respect to previously reported AE mouse models.
Moreover, for a correct interpretation of the results, a better definition of the HSC populations used throughout the study should be provided. Why was HSC and MPP phenotypic analysis carried out using different combination of antibodies (see for example Figure 2A and 4A)?

We agree with Referee 2 that more detailed information about the time scale of the experiments, the percentages of leukaemic cells in the BM and the periphery and the latencies of primary and secondary leukaemias should be better described. Supplementary Figure 4 now contains a detailed data set for the peripheral blood analysis for mice that had been DOX induced for 9 months. This Figure includes also information about the number of animals that were analysed, mean values, standard deviations, standard errors of mean and information about p-values. In the modified text we now not only mention the time when a phenotype is detectable and the latency moves into a phenotypically measurable disease stage, but we have also updated the Materials and Methods section and provide the exact experimental time scales, the genetic background of the animals used and the timing of the DOX treatment. To be more understandable, we have also redesigned the presentation of the data. This means that the absolute percentage of cells within the population of nucleated BM cells is now shown in the Figures and that Supplementary Figures contain corresponding and representative FACS plots, graphs showing relative cell percentages within the gated population and tables indicating the number of mice that were analysed, mean values, standard deviations, standard errors of mean and information about p-values. As requested, we now also have Figures distinguishing between GFP^{+} and GFP^{-} subpopulations (Figure 4, Supplementary Figures 1, 9, 10, 14 and 15). We think that these changes have greatly improved the manuscript and make it now more comprehensive.
With regard to the immune-phenotyping of HSC, we now consistently describe HSC as LT-HSC and MPP as ST-HSC which is very reasonable as MPP might also be subclassified into more maturation stages (see for example Figure 1 in Wilson et al. 2008, Cell 135, 1118-29). It is true that in the originally submitted manuscript we did apply Flt3 in addition to CD48 as a marker for HSC. The reason for this is that performing the experiments presented in the paper did take about five years of experimental work and that we did not use the Flt3 antibody when we first started to analyse the phenotype. Since typing LT-HSC as $L^{-}K^{+}S^{+}CD150^{+}CD48^{-}CD34^{-}$ and typing ST-HSC as $L^{-}K^{+}S^{+}CD150^{+}CD48^{-}CD34^{+}$ is generally accepted and commonly used by many labs (Kiel et al. 2005, Cell. 121, 1109-21, Wilson et al. 2008, Cell 135, 1118-29, Foudi et al. 2009, Nature Biotechnology 27; 84-90), we could have completely taken out the additional Flt3 marker in the revised manuscript. However, we think that it will be good to provide this data and to show Flt3 together with CD48 as HSC markers in the experiments where we have done this. We would argue that by doing this (and not deleting the Flt3 marker from the manuscript) more information with regard to the nature of stem cells we are looking at is provided.

2. In figure 1 and 2 the authors report that eight months of "moderate" AE expression in HSC and MPP did not result in the expansion of HSC and MPP. However, in the BM they detected an increased number of immature erythroid cell, mature granulocytes and megakaryocytes and reduced T and B cells. It is not clear whether the "moderate" content of AE is referred to the AE mRNA expression levels or frequency of GFP+ BM cells (Figure 1B and C). This should be clarified in the text and AE expression data should be provided also for HCS and MPP populations.

Moreover, the time when these analyses were performed is relevant information that should be included in the text and figure legend.

The Referee suggests that AE expression data for HSC and MPP (now called LT- and ST-HSC) should be provided. We were not able to do this experiment because the ROSA26-iM2-GFP mouse strain is currently not housed in our animal facility in Mainz and re-import, obligatory quarantine, breeding, genotyping and finally analysis would have taken at least four month thus exceeding the time that EMBO Molecular Medicine allows for resubmission. However, we do not think that doing this additional experiment is absolutely necessary for the following reasons: We show in the current paper that the endogenous ROSA26-M2-rtTA knock-in effector locus directs conditional GFP expression to LT-HSC and ST-HSC (Figure 1B and the corresponding Supplementary Figure 1) which is not lost upon long-term DOX treatment (Supplementary Figure 9) and also persists in leukaemic animals (Figure 4D, Supplementary Figure 14). This clearly demonstrates that with the R26/AE transplantation model the M2-rtTA transactivator is expressed in LT- and ST-HSC and induced the expression of conditional transgenes in these cells (the GFP co-reporter). Second, independent proof that the ROSA26/M2 system will direct conditional transgene expression to LT- and ST-HSC has been provided by Foudi and colleagues. In their paper the authors used M2-rtTA expression from the endogenous ROSA26 promoter to label ST- and LT-HSC with a DOX-dependent Histone2B-GFP transgene (Foudi et al. 2009, Nature Biotechnology 27; 84-90). Finally, to our best knowledge we do not know about any report in the literature that has properly investigated the expression levels of AE in t(8;21)-translocated HSC, progenitors and even adult haematopoietic lineages that have been isolated from patients. Thus we do not know the strength of AE expression in patients both during AML initiation and also in manifest disease.
For all the above reasons, we do not think it is necessary to perform this experiment. Independent of the expression levels in HSC, our paper clearly shows for the first time that the induction of a MPD-like myeloid leukaemia in mice does not depend on an increase of the ST- and LT-HSC pools. To highlight this, we say as the essential conclusion on page 11 "These results clearly show that the overall pool of LT- and ST-HSC did not expand upon long-term AE activation and proof that disease induction was not accompanied by and did not depend on the specific expansion of HSC." Also we now explicitly state in the Discussion at the bottom of page 18 that "Furthermore, we provide direct *in vivo* evidence –both during pre-leukaemia and manifest disease- that aberrant AE activation specifically expanded the GMP population but that despite an activated DOX-switch in LT- and ST-HSC the HSC pool did not increase." And go on in the Discussion on page 19 "Here, we provide direct experimental proof that mosaic expression of AE for long periods induces a MPD-like myeloid leukaemia phenotype with complete penetrance in mice."

With regard to the remaining points made by Referee 2, we have introduced the following changes: In the modified manuscript the statement about moderate AE expression has been taken out. Time-frames and DOX-schemes used in the experiment shown in Figure 1 B and C are now indicated in the corresponding legends. In addition and to make the point clear that we are working with a conditional system that allows mosaic transgene activation that is directed to a subset of cells in each analysed population, we now show in Supplementary Figure 1 representative FACS plots and tables indicating the percentage of GFP^+ cells that have an activated DOX-switch.

3. In Figure 3 some important controls are missing. These include: i) the percentage of blasts in peripheral blood and bone marrow; ii) H&E stained bone marrow sections; iii) immunohistochemical analysis to address the origin of cells infiltrating different organs. Moreover, the results obtained in age-matched-DOX transplanted mice should be shown.

Referee 2 remarks that we did not provide percentages of blasts. To provide evidence of the increased incidence of circulating blasts, we do not have **ADVIA120 or HAEMAVET** HV950 machine-generated peripheral differentials but we provide in Figure 3B a representative microscopic image demonstrating the presence of circulating blasts. Furthermore, in the current manuscript we provide representative FACS plots indicating the increase of immature myeloid cells in the BM accompanied by data from several analysed animals showing absolute and relative percentages of immature and mature granulocytes in the BM. With regard to the origin of cells infiltrating peripheral organs, we would like to refer Referee 2 to our response given on page 2 (General Concerns (subsection 1 second and third paragraph)).
With regard to H&E-stained BM sections, we depict in Figure 3D on the left a representative bone marrow section through the spinal cord of a leukaemic mouse showing also clear signs of osteo-

myelosclerosis. On the right of Figure 3D a magnification of the same image illustrates the point that leukaemic cells already leave the BM and invade the neighbouring muscle tissue. The BM of the spinal cord is a good location for investigating BM phenotypes in mice and has been previously used by our haemato-pathologist (Andreas Kreft) for analysing BM phenotypes in mice (Fritz et al. 2002, Infection and Immunity, 70, 286-291, Weber et al. Molecular Microbiology, 35, 1017-1025). Referee 2 also would like us to show in Figure 3E age-matched images of controls that were not exposed to DOX. Here we can say that the second pathologist that was involved in the analysis of the phenotype (Prof. Tony Lehr) did not see any signs of invasive leukaemic disease in organs of three age-matched controls that we did send to him together with the samples from the diseased animals. These controls were reconstituted in the same fashion as induced mice but kept in the absence of DOX for the same time. Following his analysis, we discussed the findings in both induced and control mice. Prof. Lehr has recently moved from the University of Lausanne to Germany where he has started a private enterprise. For this reason he does not conserve digital images of these experiments. To make clear that adequate long-term reconstituted controls were analysed together with the induced mice, we now state in Results in the second paragraph on page 10 “In contrast to three age-matched long-term reconstituted controls that had not been induced with DOX and indicative for invasive disease, we found infiltrates in spleen, thymus, liver, and lung of diseased animals”.

5. Figure 5B shows that DOXO removal is followed by a regression of AE leukemia in 3/7 mice. The lack of regression in these mice is an interesting finding that the authors should investigate, or at least discuss further in the text. Do the AE expression levels vary between recovered and non-recovered leukemias? Are any major chromosomal alterations or leukemias-associated mutations present in these blasts?

Following the suggestion by Referee 2 , we now discuss the lack of regression upon DOX-removal in the second paragraph on page 20 of the Discussion and state: “Although we did not address the molecular mechanisms underlying the lack of disease regression upon DOX withdrawal, several possibilities including major chromosomal aberrations, specific leukaemia-associated genetic and/or epigenetic alterations or the incomplete extinction of AE expression in resistant clones have to be envisaged” and go on in saying “because secondary mutations necessary for the onset of overt leukaemia are heterogeneous in t(8;21) AML patients (Hatlen et al, 2012), it will be crucial to develop reliable diagnostic markers to identify those patients that will benefit from AE inactivation”. We hope that by including this into the Discussion will now clearly convey that there are several possible reasons for explaining the progression of leukemic clones upon DOX-reversal and that clarifying these possibilities will be crucial for identifying patients which can benefit from the specific inactivation of AE function.

6. In my opinion, the results of transcriptome changes and bioinformatics analyses provided in Figure 7 and 8 are very preliminary and, as presented, not informative for the present study. In the absence of a correct validation of the profiles obtained, these data should be shown as supplementary material or removed from the manuscript.

We agree with referee two that the RNA-Seq data could be extended by additional experiments in the lab. However, we think that it is important to publish our RNA-Seq analysis because it provides for the first time a dynamic view of the molecular rewiring that takes place downstream of the initial AE activation in mice. Showing this data will also enable other researchers in the field to use our data for their purposes. In addition, we feel that the technology used for generating the different RNA-Seq profiles is completely justified for the questions posed and the conclusions drawn, that the execution of the technology is state of the art and that our data are absolutely sound and reasonable to include in the publication of the paper. Moreover, it is common scientific practice to compare deposited gene profiling and expression data from other groups to in house results for a new publication. Doing this means that one does not have to start from scratch again and identify, collect and analyse biopsies from large patient and control cohorts. We also think that we do not overstate our findings neither in Results nor in the Discussion. Finally, we do not make claims that are not supported by the RNA-Seq analysis and the bioinformatic methods used. For these reasons, we would like to conserve the complete RNA-Seq analysis in the paper and to show the essence of our findings in Figure 7 and 8.

7. Since leukemic mechanisms and transcriptional networks are not reported in this study, the manuscript title should be changed accordingly.

On request of Referee 2 we have now modified the title to "Instruction of haematopoietic lineage choices, evolution of transcriptional landscapes and cancer stem cell hierarchies derived from an AML1-ETO mouse model".

Minor points:
1. It would be helpful to add flow cytometric analysis plots for all the experiments where only the bar graphs are present (e.g. in Figure 1G) even if as supplementary information. In general, the FACS plots and graphs reported in the figures should be presented more clearly and more consistently labelled.

This is a very good suggestion made by Referee 2 and we completely agree that this data should be included. In the current manuscript we now present in the Figures the absolute percentages of cells within the population of all nucleated BM cells and in the Supplementary Figures representative FACS plots, relative cell percentages within the gated population and tables indicating the number of animals tested in each case, the mean percentage, the standard deviation, the standard error of mean and the resulting p-values (see also above). These general changes also include data shown in Figure 1G (which is Figure 1F in the updated manuscript) where the information is now contained in Supplementary Figures 5 and 6.

2. Statistics should be included for the data shown in Figure 5F.

The statistics to Figure 5F is now included in Supplementary Figure 16.

3. In the legend of Figure 6 the H panel is incorrect. It should be G panel

This error has now been corrected and Figure 6 has been updated in that the histogram, the relative percentage of GFP^+ granulocytes plus complementing tables showing the animals tested, mean percentages, standard errors of mean, and p values in L-HSC and L-GMP reconstituted mice are contained in Supplementary Figure 18A. In addition Supplementary Figure 18B now shows representative contour plots, relative percentages and corresponding tables for the analysis of immature and mature granulocytes in L-HSC and L-GMP reconstituted mice.

Answer to Referee 3
In this manuscript by Cabezas-Wallscheid et al.(EMM-2013-02661), the authors try to understand the effect of AML1-ETO (AE) fusion protein in the pathogenesis of core binding factor acute myeloid leukemias. Taking advantage of a new tetracycline inducible mouse model and state of the art approaches, such as whole transcriptome sequencing, the authors found that AE is able to induce, per se, leukemia in experimental animals skewing the hematopoietic differentiation program towards a myeloid lineage. Noteworthy, the leukemic status undergoes regression upon doxycycline withdrawal and oncogene exhaustion. The authors demonstrate, through isolation and transplantation of different subpopulation of leukemic cells, that tumour initiating cells are not homogeneous and that both hematopoietic stem cells and granulocyte macrophage progenitors are able to reproduce the disease in secondary recipients. RNA-sequencing of cells isolated from the bone marrow of normal, pre-leukemic and leukemicmice revealed that two different "waves of transcriptional rewiring" take place during disease progression: the first, and major one, is the direct effect of the leukemic fusion protein on the hematopoietic gene programs. The second, and a late event, is linked to the transition from pre-leukemic phase to manifest leukemia. Integrating these data with pre-existing public human leukemic database, the authors found a set of 17 genes specifically induced by AE in mouse cells and up-regulated in human and mouse leukemias (but not expressed in normal human tissues) which they propose as could serve as potential new targets for CBF-AMLs upon further investigation and validation.
This study provides additional insights for the role AE plays in the development of acute myelogenous leukemia and certainly describes for the first time a mouse model in which the fusion protein exerts a detectable oncogenic function. However, there are gaps and scientific flaws throughout the manuscript that need to be addressed before further consideration for publication.
The authors use a mouse model for conditional gene activation, generated and previously characterized by the same authors, based on tetracycline-regulated transcription activator iM2.

Wortge et al. have previously demonstrated that even if the iM2 activator is under the control of ROSA26 endogenous promoter, the transgenic mouse expresses a mosaic pattern of gene activation in the peripheral tissues. Even, within the same tissue/system, as the hematopoietic cells, there is differential expression of the reporter gene among the different subpopulations. Although this pattern of expression could be interesting in an oncogenic setting, since the oncogene is not expressed ubiquitously in all the cells, this could be detrimental here where the authors want to compare the effect of AE on different subpopulations. In addition to the percentage of GFP-positive cells, the authors should plot the mean of the fluorescence of the positive cells in Figure 1b. Since the authors assume that the expression of the reporter is proportional to AE expression, the mean of fluorescence of positive cells is more informative than the percentage.

We would like to address the comments of Referee 3 as follows:
Referee 3 puts forward that with the ROSA26-M2 mouse model there will be differences in conditional gene expression among different subpopulations and infers that the ROSA26 system does not allow for ubiquitous expression of conditional transgenes in all cells. This is absolutely right and the mosaic nature and different conditional gene expression in different haematopoietic subpopulations with the ROSA26/M2 mouse model has also been recently confirmed by others (Takiguchi et al., 2013, PLoS One; 8(1):e54009). Referee 3 goes on that we want to directly compare the effect of AE expression on different subpopulations. With regard to this we would like to clarify that we did investigate the effect of AE expression in different blood cell types by comparing genetically identical reconstituted control mice with reconstituted animals that have been exposed to DOX. We do not intend to compare the expression of the AE among different subpopulations which -as said above- might vary in different lineages. This means that it was not our intention to ubiquitously express AE in all haematopoietic cells. As already outlined in the responses to Referees 1 and 2, we think that it is a crucial advantage of the ROSA26/M2 system that AE will only be expressed in a subfraction of different blood cell types and that this mosaic expression pattern is a much better starting point for recapitulating the initial succession of events that take place in patients than a nearly complete activation of AE expression in an ubiquitous fashion.
In line with what the Referee alludes to, the authors fully admit and are completely aware of the fact that our mouse model is not perfect. However, we would argue that the R26/AE transplantation model has definitely several advantages when compared to most previously published systems because it (i) allows mosaic AE expression in blood cells, it (ii) has a fluorescent GFP co-reporter that allows following cells with an activated DOX-switch and (iii) it is reversible.

How did the authors perform the experiment in figure 1c? Was the evaluation of AE transcript done in cells isolated from total bone marrow or only in the GFP positive fraction? The authors must provide a detailed description of this experiment in the text, as this represents the most important caveat. Indeed, it is unclear whether they have evaluated the expression of AE transcript irrespective of GFP positivity. The different levels of AE expression could account for the effects the authors describe in the different cell subpopulations. In other words, is the lack of differences in HSC and MPP due to the low expression of AE in these subpopulations?

We now provide a detailed description of the experiment depicted in Figure 1C in the legend of this Figure stating “mRNA levels ±SD from three compound R26/AE mice in the absence (-DOX) or after three days of DOX exposure (+DOX) are shown”. Furthermore, we have amended the Material and Methods section accordingly (Supplementary Information page 23) saying that “RNA was extracted from BM of non-induced and induced (three days DOX) compound R26/AE mice. $L^-K^+S^+$ cells (lineage PE-conjugated B220, CD41, CD3, Gr1, CD11c, CD11b and Ter119; APC c-Kit and PE-Cy7 Sca-1 conjugated) were sorted using a FACSVantage Cell Sorter (BD) from whole BM and without gating on GFP^+ cells”.
Also connected to the Referee’s question about the qPCR experiment, we have now clarified this issue and modified in the first section of Results (on page 5 and 6). In the revised manuscript we make clear that we did not analyse AE expression levels to quantify the transcription of AE in each blood cell type but that our qPCR experiments demonstrate that AE is transcribed in different blood cell populations and without leakiness. Regarding the levels of AE expression in LT- and ST-HSC we would like to refer the Referee to our response to Referee 2 under point 2 (page 4). We think it is important that (regardless of the average AE activation levels and/or the AE levels in single cells of LT and ST-HSC in reconstituted R26/AE mice) in our mice an activated DOX-switch induces a MPD-like myeloid leukaemia phenotype and that this phenotype did not depend on and was not

accompanied by an increase in LT- and ST-HSC. To highlight this, we state in Results on page 11 "These results clearly show that the overall pool of LT- and ST-HSC did not expand upon long-term AE activation and proof that disease induction was not accompanied by and did not depend on the specific expansion of HSC." Also we now explicitly state in the Discussion at the bottom of page 18 that "Furthermore, we provide direct *in vivo* evidence –both during pre-leukaemia and manifest disease- that aberrant AE activation specifically expanded the GMP population but that despite an activated DOX-switch in LT- and ST-HSC the HSC pool did not increase.". Finally, we now state in the Discussion on page 19 "Here, we provide direct experimental proof that mosaic expression of AE for long periods induces a MPD-like myeloid leukaemia phenotype with complete penetrance in mice."

In Figure 2a, in the same vein, would the results be different if the authors pre-gate HSC and MPP of induced mice in the GFP positive fraction? If the mice are mosaics after induction,, why do the authors plot all the results as a percentage of total bone marrow instead of pre-gating the subpopulations on GFP positive fraction? Doesn't gating total bone marrow underestimate the results?

This suggestion by Referee 3 is very smart. We now have gated both in mice that were induced for < 10 months and in leukaemic animals on GFP+ and GFP- populations. Doing this unveiled that long-term DOX-induced LT- and ST-HSC had GFP+ cells were not significantly different to GFP- and also similar to non-induced controls (Supplementary Figure 9, Figure 4 and Supplementary Figure 14). What is important is that the by Referee 3 suggested GFP gating demonstrates that GFP+ LT- and ST- HSC have an activated DOX-switch and that these cells do not significantly increase upon long-term DOX-activation. We now also have applied GFP gating to progenitors and observe that the specific increase in GMP and the reduction of lineage-restricted progenitors were driven by the GFP-expressing subpopulation (Figure 4E and F and Supplementary Figures 10, 14 and 15). This indicated that the potential of AE for lineage skewing towards myelopoiesis is primarily a cell-autonomous effect.

Another important point is that the authors do not adhere to the Bethesda proposals for classification of non-lymphoid hematopoietic neoplasms in mice (Kogan et al. Blood 2002) when characterizing their model. While the authors clearly show infiltration of different organs as well as splenomegaly, they do not show either peripheral blood counts or a Kaplan-Mayer survival curve. The small differences in granulocyte phenotype in the peripheral blood (Figure 3A) look like a pre-leukemic phase rather than an overt leukemia driven by AE. Usually, the bone marrow architecture of acute leukemic mice is completely subverted and alterations are clearly appreciable in flow-cytometry using forward and side scatters. Similarly, the lack of differences in HSC, MPP, CLP (Figure 4A-B) and mild differences in GMP, MEP, CMP (Figure 4C) are more compatible with a pre-leukemic phase rather than an overt leukemic status. The same is suggested by the long survival of secondary recipients upon transplantation of fully transformed leukemic cells (Figure 5b). Again, the same concern arises from Figure 6F, why should leukemic stem cells give rise to fully differentiated granulocytes (CD11b/Gr1 double positive)? The authors should be more careful in their conclusions. Collectively, the data presented by the authors suggests that AE expression in this experimental setting induces an indolent myelo-proliferative disorder rather than a fully leukemic disorder such as CBF-AMLs. If the authors do not have further evidence to support their conclusions, they should review their manuscript and conclusions accordingly.

Indeed, in the initial version of the manuscript we did not classify our phenotype properly and according to the Bethesda proposals for the classification of non-lymphoid leukaemias. Applying these criteria we find that the phenotype seen in our mice is perfectly in accordance with (i) a non-lymphoid neoplasm diffusely involving spleen and BM, (ii) mice exhibiting (in our case) anaemia (iii) non-lymphoid cells increasing in both bone marrow and spleen and (iv) and neoplastic cells invading tissues other than blood, spleen and BM. Criterion 5 of these guidelines (at least 20% blasts in BM, blood or spleen or rapidly fatal in the primary mouse or upon transplantation) did not apply to our model. However, in the same Figure 2 of the Bethesda recommendations in the section subclassification, the experts described MPD-like myeloid leukaemia as having fewer than 20% of immature forms/blasts in haematopoietic tissues (Figure 2 page 240 in Kogan et al. 2002, Blood 100, 238-245). The in our mouse observed phenotype thus does not fulfil criterion 5 of defining leukaemia but fits into the category MPD-like myeloid leukaemia. Importantly and probably reflecting that the proposal was published in 2002, the Bethesda guidelines do not consider

leukaemic stem cells (LCS). In the current paper we have analysed these cells and find that not only L-HSC but also L-GMP acquire LSC potential. Since normal GMP can only self-renew and produce more differentiated progeny for about 4 weeks when transplanted into secondary recipients (Akhashi et al. 2000, Nature 404, 193-197) our transplanted L-GMP perfectly fulfil the criteria that define LSC (unlimited self-renewal and the production of more differentiated progeny). For this reason, we think that the best option to classify our phenotype is as indolent MPD-like myeloid leukaemia. Impotently and in order to make clear that we are not describing a mouse model that completely resembles human CBF AML, we have now changed the title of the manuscript accordingly.

The authors should state in the text how many L-HSCs and L-GMPs they transplanted in their experiment and how many mice they used to isolate leukemic cells (Figure 6). Leukemic stem cells are usually assayed using a limiting dilution or in serial transplantation assay, however, engraftment of transplanted cells does not automatically imply long-term self-renewal. Transplanting too many cells could be misleading in assessing their tumorigenic potential.

In the Materials and Methods section on page 27 of Supplementary Information we now state how many L-HSCs and L-GMPs were transplanted in each recipient and the number of mice used to isolate leukemic cells. In addition to this information, we provide a representative FACS plot of reconstituted L-HSC and L-GMP mice showing that GFP+ cells are restricted to the myeloid lineage in L-GMP recipients (Supplementary Figure 17). We now also provide detailed information about GFP+ granulocytes in the BM (Supplementary figure 18A) and the maturation state of immature and mature granulocytes (Supplementary Figure 18B). Referee 3 also points out that in LSC-assays "transplanting too many cells could be misleading in assessing their tumorigenic potential." The LSC assays we have performed for evaluating the potential of L-HSC and L-GMP followed a previously established method from the Passegue/Cleary/Weissman laboratories and we did exactly use the same number of FACS sorted L-HSC ($1x10^3$ cells) and doubled the amount of L-GMP (2×10^4 cells) when compared to the originally described method (see page 3030 in Cozzio et al. 2003, Genes Dev. 17, 3029-3035). Since roughly 50% of leukamic GMP that we used for transplantation should have an activated DOX-switch (Supplementary Figure 15) and -what is even more important-GMP will stop to self-renew and to produce progeny at about four weeks post transplantation (Akashi et al. 2000, Nature 404, 193.197), we think that it is perfectly justified to conclude that L-GMP have acquired LSC properties. This conclusion is clearly justified by the experimental findings that five month after injection our transplanted L-GMP produced high WBC counts (Figure 6A), had circulating blasts (Figure 6C) and GFP+ blasts and ring forms (Figure 6E and Supplementary Figure 18).

The authors identify a set of 17 genes specifically up-regulated during progression and increased also in human samples. Did the authors validate these genes by QPCR in leukemic and normal samples? Does the Figure 8C represent an array or a QPCR? The authors should describe the experiment better in the figure legend.

Results for identifying the 17 gens were based on bioinformatics. In the modified legend to Figure 8C we now state that we are representing data from microarrays.

The authors describe a late deregulation of "cancer related pathways" during the progression to leukemia. Since these changes are not a direct effect of AE, the authors should discuss the secondary mutations that could be responsible for this secondary event.

We agree with Referee 3 that in the whole transcriptome analysis of L-GMP we found by using IPA statistically significant changes in cancer related and anti-apoptotic pathways. However, we feel that in the updated manuscript we rather would not like to discuss possible mutations that could be responsible for secondary transcriptional events. We could of course have discussed the "usual suspects" that frequently occur in human t(8;21) patients like Kit mutations, JAK2V617F, Flt3 D853 of Flt3-ITD (indeed our RNA-Seq data supports either higher c-kit or Flt3 downstream signalling) or the sky-high up-regulation of telomerase and associated proteins conveying unlimited self-renewal. However, since we lack hard data identifying mutations (for example from exon capture or whole genome DNA-sequencing experiments), we would rather like to leave the discussion as it is and not discuss mutations that could be responsible for the second wave of transcriptional rewiring. We hope Referee 3 is happy with that.

Minor points:
Figure 1F, 1G. Use relative and not absolute numbers for y axis and then be consistent through the figures (normalizing each column to its control, plotting non-induced as 100%, will improve visualization of small differences).

We have now changed all figures accordingly and present absolute percentages within the population of nucleated BM cells (in the Figures) and relative percentages within the gated population of cells together with detailed complementary information in the accompanying tables (in the Supplementary Figures). This also applies to Figure 1F and 1G.

Figure 1G. Specify in figure legend the number of mice used in the experiment.

In the revised version of the manuscript we now have specified the number of mice used for each experiment (Tables in Supplementary Figures and in also in the Figure legends when not specified in Supplementary Figures).

Figure 2C. Specify in figure legend how long the authors induced the mice.

We now specify in the text on page 9 that the results for the LT- and ST-HSC analysis were performed with animals that had been induced between 8 and 10 months. In addition the modified Materials and Methods section gives a detailed description about all experimental details (on page 24 of the Supplementary Information section in Material and Methods section).

Does Figure 3A refer to PB or BM?

Figure 3A refers to BM. This is now clearly indicated in the specification of the y-axis.

Figure 4A-B. Are gating strategies made using control or induced mice?

Gating strategies were set according to control mice. In addition, we now provide all gating strategies for control and induced mice in the Supplementary Figures.

Figure 4C. The authors should report the number of mice.

We have done this now in the table of Supplementary Figure 12.

Figure 5A-D. The authors should report the number of mice.

The revised manuscript contains this information in the Figure legend for Figure 5 and in the accompanying tables of Supplementary Figure 16.

Figure 6D-G. The authors should report the number of mice.

We now report the number of mice in the figure legend for Figure 6 and in the accompanying tables of Supplementary Figure 18.

Does Figure 8D refer to BM or PB?

The data shown in Figure 8D data was obtained from BM and PB. The origin of the material and the study design can be obtained from the information described in the original papers and on the electronically accessible information data sheets submitted by the investigators at GSE17855 (Balgobind et al. 2011, Haematologica 96, 221-30), GSE6891 and GSE22056 (de Jonge et al, 2010 *Blood* 116, 1747-54; Verhaak et al. 2009 *Haematologica*, 94, 131-4) and GSE15061 (Mills et al. 2009 *Blood* 2009, 114, 1063-72). For readers who want to access this information we refer on page 29 of Supplementary Information in the Material and Methods section to the original sources.

2nd Editorial Decision 16 July 2013

Thank you for the submission of your revised manuscript to EMBO Molecular Medicine. We have now received the enclosed reports from the referees that were asked to re-assess it. As you will see the reviewers are now globally supportive. However, we would like you to still answer to the referees 1 and 3's comments and questions, and address the minor comments of referee 1. We also would like to encourage you to add the mean of fluorescence of GFP+ cells to indicate AE expression as strongly advised by referee 3. We feel that this point is important and would rule out that different expression of AE could be responsible for the different effects observed in the article.

In addition, we have a few editorial points that need modifying:

1/ Figure legends: please indicate when appropriate (statistical test, n, p)
2/ There is a problem with figure 5C
3/ A minimal basic materials and methods section should be present in the main manuscript as it is essential to understand the experiments performed
4/ Please incorporate the high resolution supplementary figures within the single pdf file corresponding to the Supplemental Information
5/ Supplementary tables 3 and 4 open in excel but only after giving an error formatting, please solve
6/ Provide an ethical committee approval reference number to your ethic statement
7/ Provide an accession number for the RNAseq data (to array express for example -see our guidelines for help)

I look forward to seeing a revised form of your manuscript as soon as possible.

***** Reviewer's comments *****

Referee #1 (Comments on Novelty/Model System):

From a technical point of view the authors have performed careful and well thought experiments to dissect and analyse the phenotypes induced by the expression of AE in the hematopoietic system. They tried to address experimentally the concept of 'oncogene addiction' in a model system of AML1-ETO driven acute myeloid leukaemia, one of the prominent mutations in this type of tumours. The concept of oncogene addiction has been proven for several oncogenes. However, this model is still controversial and seems to be context dependent. In this framework the efforts of Cabezas Wallscheid and co-authors constitutes a nice and novel contribution to the field. Understanding if AML1-ETO expression is required to maintain the leukemic phenotype and how gene expression is rewired by its expression may help to design future therapeutic strategies.
One important aspect/limit of the model system presented in this study is that mice harbouring AE-expressing aberrant cells do not die of leukaemia despite they mimic several aspects of the human disease (blasts appearance and morphology, invasiveness, etc.) To our knowledge this is somehow an atypical behaviour which deserves more detailed analysis in the future.

Referee #1 (Remarks):

The revised version of this manuscript is much improved and the Authors have tried to address most of our concerns. They made substantial modifications and included new data, providing a clearer explanation for the rationale of their analyses as well as limiting some of their conclusions to what their data can effectively sustain. The addition of all the gating strategies for the FACS analysis, a more accurate documentation of the details of the in vivo experiments (timing, animal strain, number of animals per group) and the consistent inclusion of a negative control (non- dox -induced) in all the figures greatly facilitates the evaluation and interpretation of their results. Most importantly, a number of analyses are now shown separately for the GFP- and GFP+ populations, demonstrating that the observed effects are clearly produced by the GFP-expressing population in the dox-treated mice. Furthermore, in the cases where no alterations were made or new data added, the authors' response and justification was detailed and, in most cases, convincing. Finally, the

results and discussion parts have been critically revised, taking into account the concerns raised by all referees.
The only (remaining) weak point is the lack of AE protein expression analysis, which in my opinion is relevant and should have been demonstrated at least in the description of the model, even though the Authors refer to previous studies to justify why they did not perform or show these data.

Minor comments
1. Figure 1 legend, letter F and G should be substituted with E and F
2. Figure 5 B, D and F: It would be interesting to show if there are statistically significant differences between the blue dot group and the red and/or orange one
3. Figure 5C: The +Dox picture does not show properly
4. Figure 5F: Why are only two out of three animals of the blue dot group shown? (It is also mentioned as such in the text, but without any further explanation for the exclusion of the third animal from the analysis)
5. Figure 8D legend, the third sentence needs to be checked and revised

Referee #2 (Remarks):

The authors have clearly improved the manuscript by including additional experimental details and experiments, which support their conclusions.

Referee #3 (Remarks):

The revised manuscript by Cabezas-Wallscheid et al. certainly provides new insights for the role of AE during leukemic progression. The authors addressed all the minor points, and to some extent the major points. Specifically, they addressed the issue regarding the features of indolent myelo-proliferative disorder (MPD) compared to fully leukemic disorder, and replaced "acute myeloid leukemia" with the more conservative, "indolent MPD-like myeloid leukaemia" throughout the manuscript.
Although the authors provided more details on the assays used to define the leukemic stem cells, some questions still remain, namely, why would leukemic stem cells (L-HSC) give rise to both myeloid and lymphoid cells (as described in Fig.6 and Supp. Fig.17)? Why do they generate lymphoid cells? This is an unexpected result from a leukemic stem cell which is only required to recreate leukemic myeloid blasts by definition. Instead of highlighting this result, the authors should carefully comment on it.
The authors also did not address the most important concern of all, that is, the expression levels of AE in the different subpopulations. This is the most important caveat in the manuscript because the different expression levels of AE could account for the different effects the authors describe. Statements such as, "These results clearly show that the overall pool of LT- and ST-HSC did not expand upon long-term AE activation and provide proof that disease induction was not accompanied by and did not depend on the specific expansion of HSC" are not supported by data, and the authors should remove them from the manuscript if the expression levels of AE in the corresponding subpopulations are not provided. A lower expression of AE could account for the lack of effects described in the stem cell compartment with respect to others. One simple way to address this point is by providing the mean of fluorescence of GFP positive cells in the different subpopulations, which the authors should already have without performing further experiments. If the authors modify the manuscript accordingly, I recommend the publication in EMBO Molecular Medicine.

2nd Revision - authors' response 28 August 2013

Final response letter to the referees

We were very pleased about the positive answers of both referees and are happy to address the remaining points concerning our manuscript. In particular, we liked very much that Referee 1 thinks that our work constitutes a nice and novel contribution to the field and that Referee 2 states that the revised manuscript certainly provides new insights for the role of AE during leukemic progression.

We also would like to thank both referees for their thorough and creative reading of the revised manuscript and also for picking up several additional minor points that now have been modified.

Answer to Referee 1
Referee 1 (Comments on Novelty/Model System):

From a technical point of view the authors have performed careful and well thought experiments to dissect and analyse the phenotypes induced by the expression of AE in the hematopoietic system. They tried to address experimentally the concept of 'oncogene addiction' in a model system of AML1-ETO driven acute myeloid leukaemia, one of the prominent mutations in this type of tumours. The concept of oncogene addiction has been proven for several oncogenes. However, this model is still controversial and seems to be context dependent. In this framework the efforts of Cabezas Wallscheid and co-authors constitutes a nice and novel contribution to the field. Understanding if AML1-ETO expression is required to maintain the leukemic phenotype and how gene expression is rewired by its expression may help to design future therapeutic strategies.
One important aspect/limit of the model system presented in this study is that mice harbouring AE-expressing aberrant cells do not die of leukaemia despite they mimic several aspects of the human disease (blasts appearance and morphology, invasiveness, etc.) To our knowledge this is somehow an atypical behaviour which deserves more detailed analysis in the future.

We completely agree with Referee 1 that an important aspect/limit of our model system is that despite the fact that the phenotype mimics several aspects of the human disease, we did not observe lethality and that this issue should addressed in the future. We think that one possible explanation for the lack of lethality is that we used conditional transgene activation from the ROSA26 locus that is known to direct moderate expression to only a subset of cells.

Referee #1 (Remarks):

The revised version of this manuscript is much improved and the Authors have tried to address most of our concerns. They made substantial modifications and included new data, providing a clearer explanation for the rationale of their analyses as well as limiting some of their conclusions to what their data can effectively sustain. The addition of all the gating strategies for the FACS analysis, a more accurate documentation of the details of the in vivo experiments (timing, animal strain, number of animals per group) and the consistent inclusion of a negative control (non- dox -induced) in all the figures greatly facilitates the evaluation and interpretation of their results. Most importantly, a number of analyses are now shown separately for the GFP- and GFP+ populations, demonstrating that the observed effects are clearly produced by the GFP-expressing population in the dox-treated mice. Furthermore, in the cases where no alterations were made or new data added, the authors response and justification was detailed and, in most cases, convincing. Finally, the results and discussion parts have been critically revised, taking into account the concerns raised by all referees.
The only (remaining) weak point is the lack of AE protein expression analysis, which in my opinion is relevant and should have been demonstrated at least in the description of the model, even though the Authors refer to previous studies to justify why they did not perform or show these data.

We were very pleased to see that the changes and additional data that we had introduced to the updated manuscript convinced the Referee and that he/she thinks that these changes provide a clearer explanation for the rationale of the experiments. We are also happy to see that the Referee states that our conclusions are effectively sustained by the data. Moreover and based on the initial suggestions made by our Referees, we agree that it was an excellent suggestion to differentiate between GFP^+ and GFP^- populations thus showing that the *in vivo* effects produced by conditional activation of the transgene are produced in the GFP^+ sub-population. Finally, we are happy that Referee 1 thinks that we did address the comments and queries convincingly. With regard to the only remaining weak point (that we do not show AE protein expression but only AE-specific mRNA transcription), we would like to follow the recommendation of Referee 2. In the current manuscript we now have added the median fluorescence levels in LT-, ST-HSC, CMP, GMP and MEP (Supplementary Figure 1B and first sentence on page 7 of the updated manuscript) for reporting the activation level of the DOX-switch. Since GFP fluorescence in our model directly reflects the expression levels of the iM2 rtTA transactivator and thus is a direct measure for the strength of the DOX-switch in each sub-population, we think that it is not absolutely necessary to include a

Western blot analysis showing protein levels. This is also supported by our finding of similar median GFP fluorescence levels that are present in LT-, ST-HSC and progenitors (new figure 1B on page 3 of Supplementary data). We also strongly feel that providing Western blotting data for DOX-induced whole BM cells is not sufficient for demonstrating protein expression for the different subpopulations (for example LT-HSC). As a matter of fact for demonstrating protein expression it is necessary to purify sufficient LT-, ST-HSC and progenitors by MACS/FACS followed by Western blotting. Considering the low frequency of for example LT-HSC in the BM such experiments would be very expensive and time consuming and will not be possible to conduct within a reasonable and limited time frame for publication. I thus strongly feel that the inclusion of protein data would be perfect but is not absolutely necessary because (i) the original description of the tetO-AML1-ETO mouse model provides a clear proof that AML1-ETO protein expression is induced by an active DOX-switch, because (ii) we provide experimental evidence that the tet-O-EGFP switch is activated in LT-, ST-HSC and blood cell progenitors upon DOX exposure (functional and comparable GFP protein expression in HSC and progenitors), because (iii) we show that AE-specific mRNA is produced in LT-, ST-HSC and progenitors. Finally and to my best knowledge, I do think that although in human patients AE mRNA have been analysed in different blood cell types, sufficient information about AE protein expression in LT-, ST-HSC and blood cell progenitors is missing. On these grounds and since analysis of protein expression in the different mature and immature lineages will be not possible within the time line for publishing in EMBO Molecular Medicine, we hope that Referee 1 will not insist on showing protein data.

Minor comments of Referee 1

1. Figure 1 legend, letter F and G should be substituted with E and F.

In the legend to Figure 1 F and G are now substituted by E and F.

2. Figure 5 B, D and F: It would be interesting to show if there are statistically significant differences between the blue dot group and the red and/or orange one.

Figure 5B, 5D and 5F now show the statistical analysis between the blue dot and the red and/or orange groups. In addition, we have also amended Supplementary Figure 16, showing under 16B on page 18 of Supplementary Data an extended new table with the corresponding p values between the blue dot group and the red and/or orange dot group.

3. Figure 5C: The +Dox picture does not show properly.

This error has now been corrected and Figure 5C is now showing properly.

4. Figure 5F: Why are only two out of three animals of the blue dot group shown? (It is also mentioned as such in the text, but without any further explanation for the exclusion of the third animal from the analysis).

The Referee is right that in the original manuscript only two animals (2 blue dots) were shown in Figure 5 F. This error is now corrected both in Figure 5F in Supplementary Figure 16.

5. Figure 8D legend, the third sentence needs to be checked and revised.

We have now checked and revised the third sentence of figure legend 8D. The corrected legend reads now: On the right 61 individual gene transcription profiles from t(8;21) AML biopsies (t(8;21) patients) and 69 individual gene transcription profiles from healthy donors (controls) are shown.

Answer to Referee 2

Referee #2 (Remarks):

The authors have clearly improved the manuscript by including additional experimental details and experiments, which support their conclusions.

Referee #2 (Remarks):

The revised manuscript by Cabezas-Wallscheid et al. certainly provides new insights for the role of AE during leukemic progression. The authors addressed all the minor points, and to some extent the major points. Specifically, they addressed the issue regarding the features of indolent myelo-proliferative disorder (MPD) compared to fully leukemic disorder, and replaced "acute myeloid leukemia" with the more conservative, "indolent MPD-like myeloid leukaemia" throughout the manuscript. Although the authors provided more details on the assays used to define the leukemic stem cells, some questions still remain, namely, why would leukemic stem cells (L-HSC) give rise to both myeloid and lymphoid cells (as described in Fig.6 and Supp. Fig.17)? Why do they generate lymphoid cells? This is an unexpected result from a leukemic stem cell which is only required to recreate leukemic myeloid blasts by definition. Instead of highlighting this result, the authors should carefully comment on it.

We agree with Referee 3 that the nomenclature for the observed phenotype as indolent MPD-like myeloid leukaemia is much better for classifying the observed murine pathology. One remaining concern of the Referee is that we show in Figure 6C that L-HSC animals have aberrant red blood cells and that in the FACS data of Supplementary Figure 17 we show that the population of FACS purified L-HSC gives rise to both myeloid and lymphoid cells. We completely agree with the Referee that if we would have used for our experiments only those HSC that have been fully transformed, one should not expect a multi-lineage output but only the restricted production of leukaemic blast-like myeloid cells with or without a differentiation defect (for example in a setup using ex-vivo virally transduced BM HSC co-expressing a strong leukaemogene together with GFP and using only the GFP^+ population in the reconstitution experiment). We have not done this because our system is different and since the question we have asked in our experiments was if both cells having HSC and GMP surface markers will be able to propagate the disease when transferred into recipient mice. The fact that HSC taken from leukemic mice (which we call L-HSC defined as $L^+K^+S^+CD150^+$) do have the potential to contribute to myeloid, erythroid and lymphoid lineages is under our experimental conditions to be expected because we did gate on immune-phenotypic $L^+K^+S^+CD150^+$ markers thus isolating all HSC containing these markers and not only malignant HSC. This means that we have purified a population of malignant and normal HSC that can give rise to multi-lineage differentiation (the normal HSC fraction) and also can produce malignant blasts (the malignant HSC fraction). To leave no doubt about the fact that the L-HSC and L-GMP populations purified by the above markers were containing a mixture of leukaemic and normal cells, we now state at the end of page 12 of the manuscript "To establish whether only cells expressing HSC markers or also cells with a more lineage-restricted immune phenotype can act as LSC in our model, we tested the potential of leukaemic HSC (L-HSC defined as $L^+K^+S^+CD150^+$ containing both malignant and normal HSC) and leukaemic GMP (L-GMP defined as $L^-K^+S^-IL7R\alpha^-$ $CD34^+Fc\gamma RII/III^+$ containing both malignant and normal GMP) to propagate the disease.

The authors also did not address the most important concern of all, that is, the expression levels of AE in the different subpopulations. This is the most important caveat in the manuscript because the different expression levels of AE could account for the different effects the authors describe. Statements such as, "These results clearly show that the overall pool of LT- and ST-HSC did not expand upon long-term AE activation and provide proof that disease induction was not accompanied by and did not depend on the specific expansion of HSC" are not supported by data, and the authors should remove them from the manuscript if the expression levels of AE in the corresponding subpopulations are not provided. A lower expression of AE could account for the lack of effects described in the stem cell compartment with respect to others. One simple way to address this point is by providing the mean of fluorescence of GFP positive cells in the different subpopulations, which the authors should already have without performing further experiments. If the authors modify the manuscript accordingly, I recommend the publication in EMBO Molecular Medicine.

Referee 2 is right that lower expression of AE could account for the lack of effects described in the stem cell compartment. Following his/her suggestions the updated manuscript now provides the quantification of the fluorescence of GFP positive cells in different subpopulations. In the final version of the manuscript this data is shown in Supplementary Figure 1B on page 3 of Supplementary data depicting the median fluorescence values of GFP^+ LT-HSC, ST-HSC, CMP, GMP and MEP populations from four R26/AE reconstituted C57BL/6 mice that have been DOX-induced for ten days, representing the relative intensity value below which 50% of the events are found. We choose to show median values because the calculation of median fluorescence is a more robust estimator of the tendency of a population than the mean (http://www.flowjo.com/vX/en/ws.statistics.html). Our analysis shows that LT-, ST-HSC and progenitors have very similar median fluorescence levels directly indicating that transgene activation is comparable in LT-, ST-HSC and progenitors.

Finally, in a recent report that was published while this paper was under revision, Miraki-Moud and collaborators found that 16 patients with AML had similar numbers of $CD34^+CD38^-$ BM HSC as did 42 healthy controls (Miraki-Moud et al. 2013, *PNAS* 110, 13576-13581). To our knowledge Miraki-Moud and collaborators are the first to experimentally analyse HSC numbers in AML patients. Although in the paper the authors do not explicitly specify the molecular lesions of the 16 AML cases analysed (the samples are classified as subtype A) and although one must be very cautious when comparing humans and mice, the data directly supports the notion that AML patients do not have increased pools of HSC.

We hope that Referee 2 is satisfied with the introduced modifications and corrections and will support the publication of our improved and final manuscript.
