# Supplementary material for: Instruction of haematopoietic lineage choices, evolution of transcriptional landscapes and cancer stem cell hierarchies derived from an AML1-ETO mouse model
Source: EMBO Mol Med. 2013 Oct 4;5(12):1804–20. doi: 10.1002/emmm.201302661 (PMC3914523; doi:10.1002/emmm.201302661)
Supplement: Supplementary file 2 [file emmm0005-1804-sd2.pdf]

SUPPLEMENTARY DATA

TABLE OF CONTENTS

Supplementary figures **page 2-20**

Information about supplementary tables **page 21**

Materials and Methods **page 22-30**

References for Materials and Methods **page 31-33**

SUPPLEMENTARY FIGURES

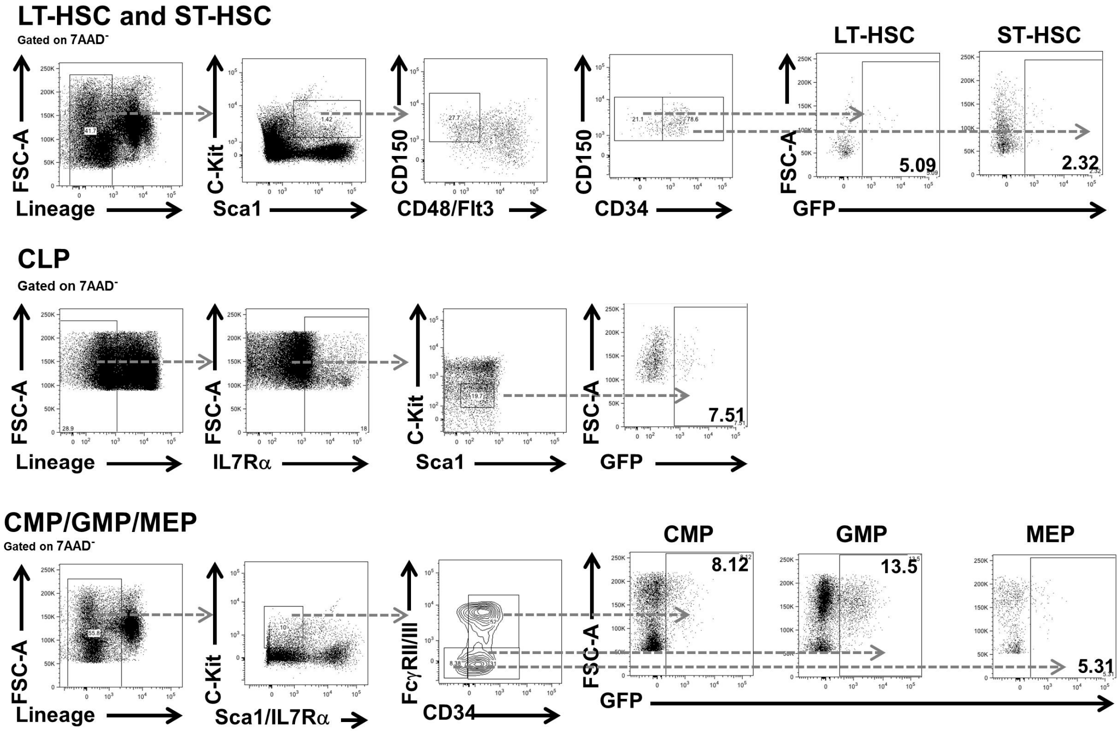

Percentage of GFP+ cells in each linage (relative % of gated cells)

Population	LT-HSC		ST-HSC		CLP		CMP		GMP		MEP	
	GFP+	GFP-	GFP+	GFP-	GFP+	GFP-	GFP+	GFP-	GFP+	GFP-	GFP+	GFP-
Number of animals tested	4	4	4	4	4	4	4	4	4	4	4	4
Mean percentage	**2.15**	97.85	**2.465**	97.535	**9.44**	90.56	**8.94**	91.06	**7.093**	92.907	**4.78**	95.22
Standard deviation	1.244	98.756	1.763	98.237	5.175	94.825	0.7614	99.2386	6.362	93.638	1.874	98.126
Standard error of mean	0.622	99.378	0.8817	99.1183	2.588	97.412	0.3807	99.6193	3.181	96.819	0.9369	99.0631

Supplementary Figure 1A: The R26/AE mouse model directs GFP transgene expression to a subset of HSC and blood cell progenitors

Representative FACS profiles of BM cells from R26/AE reconstituted C57BL/6 mice that have been DOX-induced for ten days are depicted. FACS plots showing LT- and ST-HSC were gated on $7AAD^-/L^-K^+S^+/CD48^-/Flt3^-/CD150^+$ cells and subclassified for the expression of $CD34^-$ (LT-HSC) and $CD34^+$ (ST-HSC). The CLP fraction was gated for $7AAD^-/L^-K^{int}S^{int}$/IL7receptor α^+ cells. GMP, CMP and MEP were gated on $7AAD^-/L^-K^+S^-$/ IL7 receptor α^- cells and subclassified based on the expression of Fcγ receptor and CD34. Numbers indicate the percentage of GFP^+ cells within each population. The table at the bottom shows the number of animals that were analysed, mean percentages of GFP^+ and GFP^- cells, standard deviations and standard errors of mean for LT-, ST-HSC and lineage restricted blood cell progenitors.

B

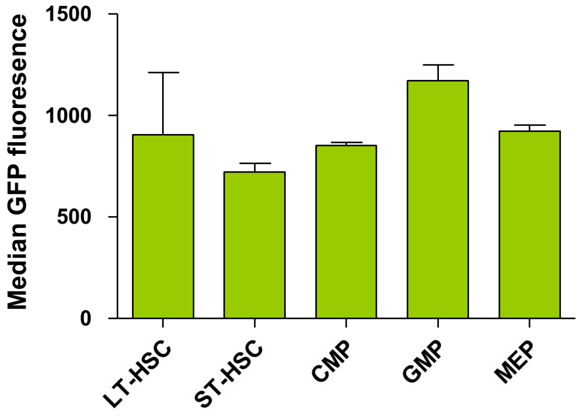

Median GFP fluorescence of LT-HSC, ST-HSC, CMP, GMP and MEP

Gating	GFP+ cells				
Population	LT-HSC	ST-HSC	CMP	GMP	MEP
Number of animals tested	4	4	4	4	4
Median GFP fluorescence	904.8	721.5	851.8	1171	925.8
Standard deviation	617	85.77	31.75	155.8	56.81
Standard error	308.5	42.88	15.88	77.92	28.4

Supplementary Figure 1B: Median fluorescence of GFP^{+} haematopoietic stem cells and lineage-restricted blood cell progenitors

Median fluorescence values of GFP^{+} LT-, ST-HSC, CMP, GMP and MEP populations from four R26/AE reconstituted C57BL/6 mice that have been DOX-induced for ten days are depicted in the graph on the top. LT- and ST-HSC were gated on 7AAD^{-}/L^{-}K^{+}S^{+}/CD48^{-}/Flt3^{-}/CD150^{+}/GFP^{+} cells and subclassified for the expression of CD34^{-} (LT-HSC) and CD34^{+} (ST-HSC). GMP, CMP and MEP were gated on 7AAD^{-}/L^{-}K^{+}S^{-}/ IL7 receptor α^{-}/GFP^{+} expression and subclassified based on the expression of Fcγ receptor and CD34. The median fluorescence is represented as the relative intensity value below which 50% of the events were found within each population. The table at the bottom shows the number of animals that were analysed, median GFP fluorescence values, standard deviations and standard errors.

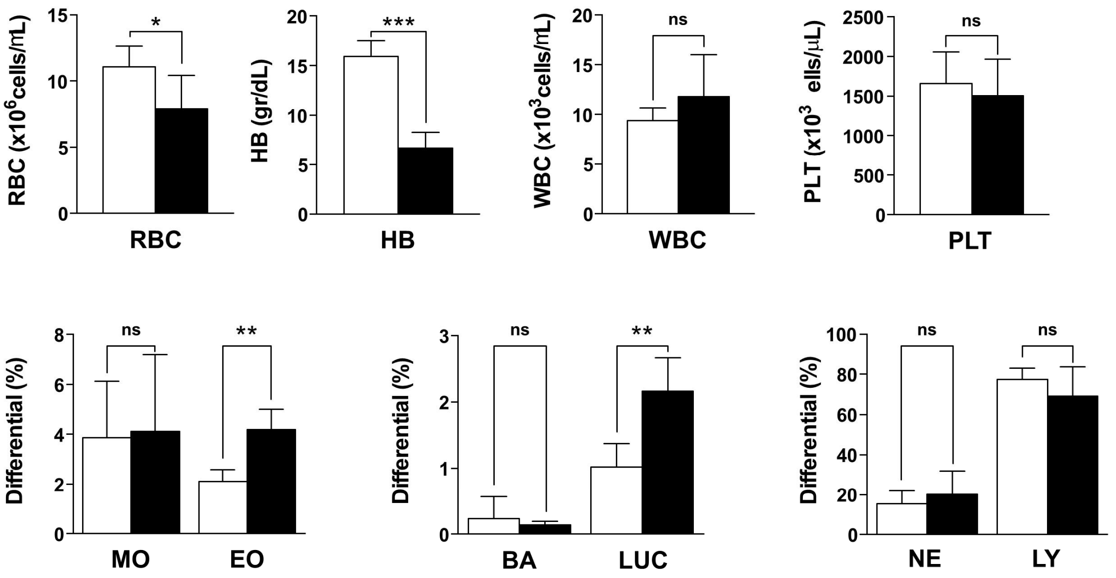

Peripheral blood parameters

Population	RBC		HB		WBC		PLT		MO		EO		BA		LUC		NE		LY	
	Control	AE	Control	AE	Control	AE	Control	AE	Control	AE	Control	AE	Control	AE	Control	AE	Control	AE	Control	AE
Number of animals tested	5	5	5	5	5	5	5	5	5	5	5	5	6	5	6	5	5	5	5	5
Mean	11,08	7,880	15,90	6,664	9,368	11,81	1554	1504	3,860	4,120	2,100	4,180	0,2333	0,1400	1,017	2,160	15,48	20,24	77,58	69,12
Standard deviation	1,563	2,532	1,624	1,594	1,270	4,232	420,2	460,7	2,270	3,073	0,4690	0,8228	0,3327	0,05477	0,3545	0,5595	6,491	11,56	5,384	14,69
Standard error of mean	0,6990	1,132	0,7261	0,7128	0,5679	1,893	187,9	206,0	1,015	1,374	0,2098	0,3680	0,1358	0,02449	0,1447	0,2502	2,903	5,169	2,408	6,570
P value summary	*		***		ns		ns		ns		**		ns		**		ns		ns	
P value (unpaired t test)	0.043		<0.0001		0.2519		0.8633		0.8828		0.0012		0.5538		0.0026		0.4452		0.2611	

Supplementary Figure 2: Peripheral blood parameters in control and AE-expressing mice

The bar diagrams show mean peripheral blood parameters ±SD from R26/AE reconstituted RAG2$^{-/-}$ control mice without DOX exposure (white bars) and reconstituted animals that have been DOX-induced during nine months (black bars). RBC, red blood cell count; HB, haemoglobin; WBC, white blood cell count; PLT, platelet count; MO, monocyte count; EO, eosinophil count; BA, basophil count; LUC, large myeloperoxidase-negative cell count; NE, neutrophil count; LY, lymphocyte count. The table at the bottom shows the number of animals that were analysed, mean percentages, standard deviations and standard errors of mean, p value summaries and p values for each peripheral blood cell parameter.

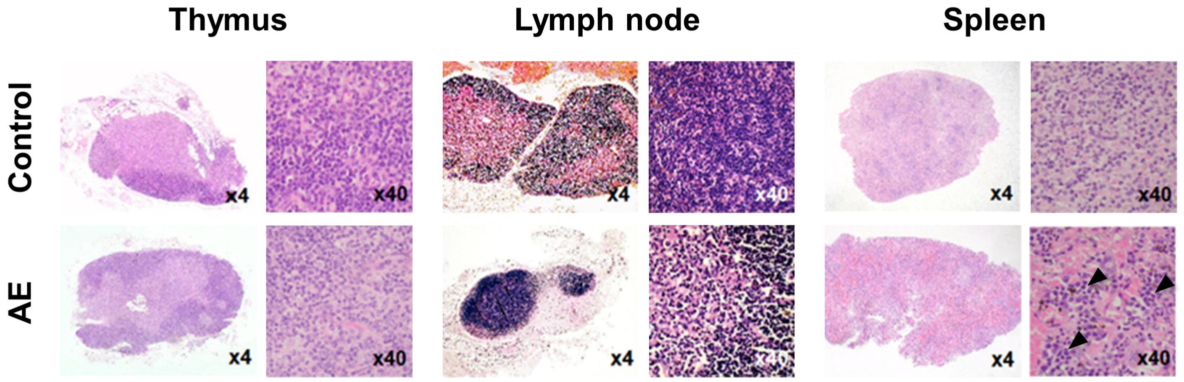

Supplementary Figure 3: Histological sections through haematopoietic organs from control and AE-expressing mice

Representative H&E-stained sections from R26/AE reconstituted RAG2$^{-/-}$ control mice without DOX exposure (upper panel) and reconstituted animals that have been DOX-induced during nine months (lower panel) are shown for the thymus, the lymph nodes and the spleen. For each organ the general appearance (x4) together with a higher magnification (x40) are shown. The apparent increase in erythropoiesis in the red pulp of the AE-expressing animal is indicated by black arrowheads.

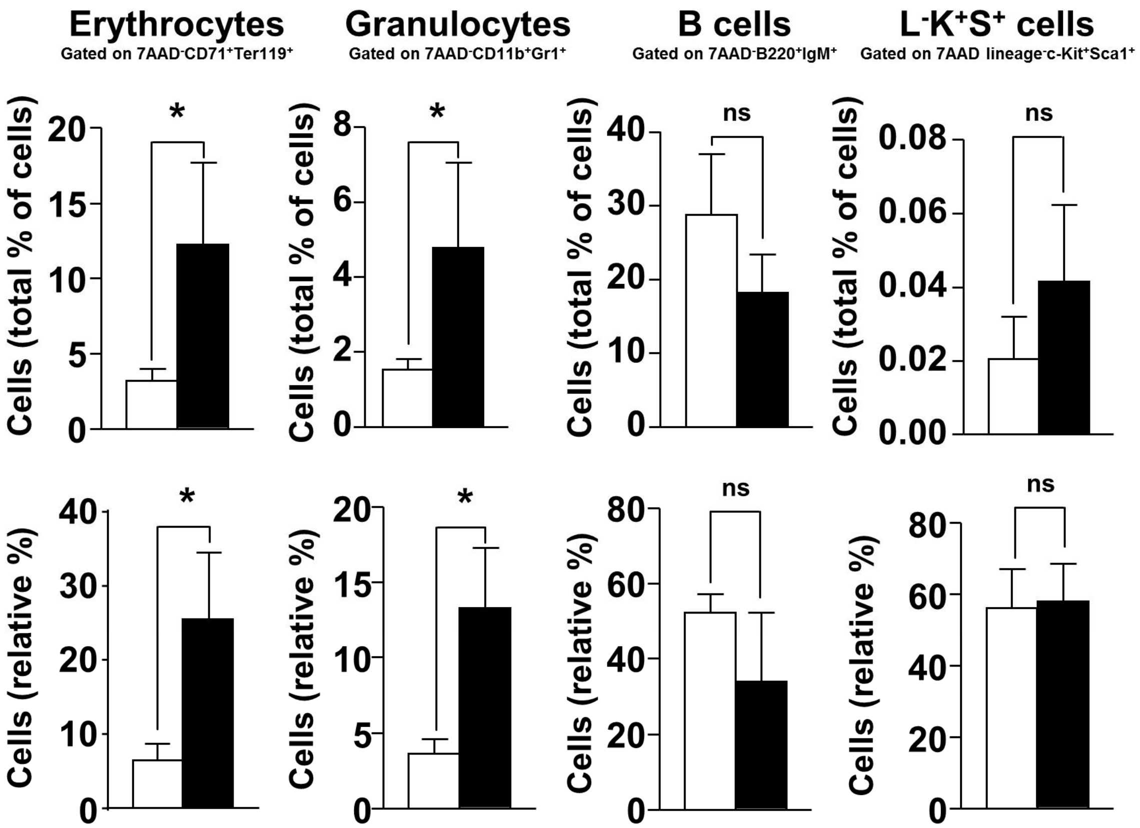

Flow cytometry of the spleen (absolute % of total splenic cells)

Population	Erythrocytes		Granulocytes		B cells		L-K+S+	
	Control	AE	Control	AE	Control	AE	Control	AE
Number of animals tested	3	3	3	4	5	4	5	5
Mean	3.22	12.29	1.523	4.775	28.8	18.25	0.0206	0.0416
Standard deviation	0.7817	5.381	0.293	2.261	8.244	5.173	0.01133	0.02057
Standard error of mean	0.4513	3.106	0.1691	1.13	3.687	2.587	0.005066	0.009201
P value summary	*		*		ns		ns	
P value (unpaired t test)	0.0446		0.034		0.0621		0.0806	

Flow cytometry of the spleen (relative % of gated cells)

Population	Erythrocytes		Granulocytes		B cells		L-K+S+	
	Control	AE	Control	AE	Control	AE	Control	AE
Number of animals tested	3	3	3	4	5	5	5	5
Mean	6.533	25.53	3.657	13.3	52.42	33.89	56.2	58.1
Standard deviation	2.206	9.473	0.9354	3.981	4.766	18.53	10.9	10.38
Standard error of mean	1.274	5.469	0.5401	1.99	2.132	8.286	4.873	4.641
P value summary	*		*		ns		ns	
P value (unpaired t test)	0.0277		0.0101		0.0623		0.7848	

Supplementary Figure 4: Flow cytometric analysis of extramedullary haematopoiesis in control and AE-expressing spleens

The bar diagrams show total and relative percentages of erythrocytes, granulocytes, B and $L^-K^+S^+$ cells from R26/AE reconstituted $RAG2^{-/-}$ control mice without DOX exposure (white bars) and reconstituted animals that have been DOX-induced during nine months (black bars). In the upper row of plots the absolute percentages of cells within the population of nucleated spleen cells is shown. The lower plots depict the relative percentages of cells within each cell type that was analysed. The tables at the bottom contain information about the number of animals that were tested, mean absolute and relative percentages, standard deviations, standard errors of mean, p value summaries and p values for each cell type.

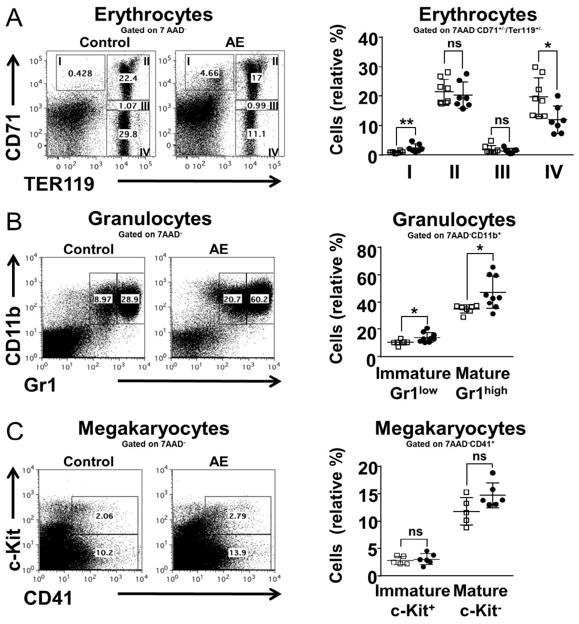

Erythrocytes (absolute % of total BM cells)

Population	I		II		III		VI	
	Control	AE	Control	AE	Control	AE	Control	AE
Number of animals tested	8	7	8	7	8	7	8	7
Mean	0.5213	1.493	12.38	12.57	1.051	0.88	11.55	7.473
Standard deviation	0.1589	0.797	4.278	2.754	0.9219	0.5023	5.41	3.148
Standard error of mean	0.05617	0.3012	1.513	1.041	0.3259	0.1898	1.913	1.19
P value summary	**		ns		ns		ns	
P value (unpaired t test)	0.0048		0.9201		0.6695		0.1039	

Erythrocytes (relative % of gated cells)

Population	I		II		III		VI	
	Control	AE	Control	AE	Control	AE	Control	AE
Number of animals tested	8	7	8	7	8	7	8	7
Mean	0.935	2.419	21.4	20.26	1.789	1.417	19.65	11.91
Standard deviation	0.2957	1.335	4.327	4.539	1.399	0.8147	6.687	4.66
Standard error of mean	0.1046	0.5047	1.53	1.716	0.4948	0.3079	2.364	1.761
P value summary	**		ns		ns		*	
P value (unpaired t test)	0.0089		0.6262		0.5488		0.0236	

Granulocytes (absolute % of total BM cells)

Population	Immature		Mature	
	Control	AE	Control	AE
Number of animals tested	7	9	7	9
Mean	6.07	8.39	20.73	28.93
Standard deviation	1.163	1.92	2.75	8.379
Standard error of mean	0.4396	0.64	1.039	2.793
P value summary	*		*	
P value (unpaired t test)	0.0139		0.0269	

Granulocytes (relative % of gated cells)

Population	Immature		Mature	
	Control	AE	Control	AE
Number of animals tested	7	9	7	9
Mean	10.14	13.84	34.51	47.01
Standard deviation	1.825	3.583	2.831	11.99
Standard error of mean	0.6899	1.194	1.07	3.996
P value summary	*		*	
P value (unpaired t test)	0.0262		0.0179	

Magakaryocytes (absolute % of total BM cells)

Population	Immature		Mature	
	Control	AE	Control	AE
Number of animals tested	4	6	4	6
Mean	1.66	1.918	7.023	8.55
Standard deviation	0.5582	0.491	1.876	0.702
Standard error of mean	0.2791	0.2005	0.9378	0.287
P value summary	ns		ns	
P value (unpaired t test)	0.4613		0.1007	

Magakaryocytes (relative % of gated cells)

Population	Immature		Mature	
	Control	AE	Control	AE
Number of animals tested	5	6	5	6
Mean	2.722	3.25	11.83	16.63
Standard deviation	0.7725	1.509	2.564	7.115
Standard error of mean	0.3455	0.5336	1.147	2.515
P value summary	ns		ns	
P value (unpaired t test)	0.4881		0.1804	

Supplementary Figure 5: Flow cytometric analysis of control and AE-expressing mice reveals red blood cell lineage maturation defects and the expansion of granulocytes

(A) Representative FACS profiles of BM cells from R26/AE reconstituted $RAG2^{-/-}$ mice that have been DOX-induced for nine months are depicted on the left. FACS plots showing red blood cell maturation were gated on $7AAD^{-}$ cells and subclassified into I (proerythroblasts, $CD71^{+}Ter119^{-}$); II (basophilic erythroblasts, $CD71^{+}Ter119^{+}$); III (late basophilic erythroblasts and chromatophilic erythroblasts, $CD71^{int}Ter119^{+}$) and IV (orthochromatophilic erythroblasts and reticulocytes, $CD71^{-}Ter119^{+}$). The graph shows relative percentages of control (white squares) and DOX-induced (black dots) cells within the four analysed red blood cell populations with each square and dot representing one individual mouse. The two tables on the right indicate mean absolute (upper table) and mean relative (lower table) cell percentages, the number of animals that were analysed, standard deviations, standard errors of mean, p value summaries and p values for each maturation stage.
(B) Representative FACS profiles of BM cells from R26/AE reconstituted $RAG2^{-/-}$ mice that have been DOX-induced for nine months are depicted on the left. FACS plots showing granulocytes were gated on $7AAD^{-}$ cells and subclassified into immature ($CD11b^{+}Gr1^{low}$) and mature ($CD11b^{+}Gr1^{high}$) stages. The graph shows relative percentages of control (white squares) and DOX-induced (black dots) cells within the two analysed granulocytic populations with each square and dot representing one individual mouse. The two tables on the right indicate mean absolute (upper table) and mean relative (lower table) cell percentages, the number of animals that were analysed, standard deviations, standard errors of mean, p value summaries and p values for each maturation stage.
(C) Representative FACS profiles of BM cells from R26/AE reconstituted $RAG2^{-/-}$ mice that have been DOX-induced for nine months are depicted on the left. FACS plots showing megakaryocytes were gated on $7AAD^{-}$ cells and subclassified into immature ($CD41^{+}c\text{-}Kit^{+}$) and mature ($CD41^{+}c\text{-}Kit^{-}$) stages. The graph shows relative percentages of control (white squares) and DOX-induced (black dots) cells within the two analysed megakaryocytic populations with each square and dot representing one individual mouse. The two tables on the right indicate mean absolute (upper table) and mean relative (lower table) cell percentages, the number of animals that were analysed, standard deviations, standard errors of mean, p value summaries and p values for each maturation stage.

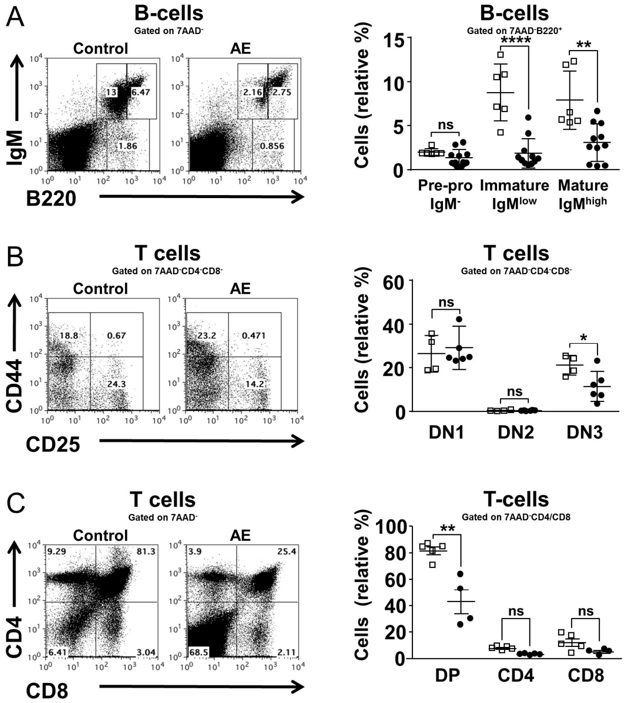

B cells (absolute % of total BM cells)

Population	Pre-pro		Immature		Mature	
	Control	AE	Control	AE	Control	AE
Number of animals tested	6	11	6	11	6	11
Mean	0.9315	0.6435	4.018	0.8449	3.737	1.49
Standard deviation	0.1723	0.433	1.39	0.6388	1.854	1.033
Standard error of mean	0.07032	0.1306	0.5676	0.1926	0.7571	0.3114
P value summary	ns		****		**	
P value (unpaired t test)	0.1431		<0.0001		0.0054	

B cells (relative % of gated cells)

Population	Pre-pro		Immature		Mature	
	Control	AE	Control	AE	Control	AE
Number of animals tested	6	11	6	11	6	11
Mean	2.008	1.352	8.745	1.854	7.865	3.074
Standard deviation	0.375	0.9314	3.231	1.664	3.305	2.112
Standard error of mean	0.1531	0.2808	1.319	0.5018	1.349	0.6367
P value summary	ns		****		**	
P value (unpaired t test)	0.1229		<0.0001		0.0023	

T-cells (absolute % of total thymic cells)

Population	DN1		DN2		DN3	
	Control	AE	Control	AE	Control	AE
Number of animals tested	4	6	4	6	4	6
Mean	1.145	1.69	0.01485	0.01963	0.8675	0.5667
Standard deviation	0.5268	0.9449	0.007829	0.01149	0.1269	0.2308
Standard error of mean	0.2634	0.3858	0.003914	0.004692	0.06343	0.09422
P value summary	ns		ns		*	
P value (unpaired t test)	0.3298		0.4912		0.0467	

T cells (relative % of gated cells)

Population	DN1		DN2		DN3	
	Control	AE	Control	AE	Control	AE
Number of animals tested	4	6	4	6	4	6
Mean	26.4	29.17	0.3675	0.34	21.05	11.42
Standard deviation	8.59	10.02	0.2179	0.1413	4.497	6.87
Standard error of mean	4.295	4.09	0.109	0.05768	2.249	2.804
P value summary	ns		ns		*	
P value (unpaired t test)	0.6641		0.8128		0.0399	

T cells (absolute % of total thymic cells)

Population	DP		CD4		CD8	
	Control	AE	Control	AE	Control	AE
Number of animals tested	5	6	5	6	5	6
Mean	54.82	20.24	5.106	4.307	1.998	1.977
Standard deviation	9.948	17.41	0.9909	3.441	0.4052	1.421
Standard error of mean	4.449	7.109	0.4432	1.405	0.1812	0.5803
P value summary	**		ns		ns	
P value (unpaired t test)	0.0035		0.6301		0.975	

T cells (relative % of gated cells)

Population	DP		CD4		CD8	
	Control	AE	Control	AE	Control	AE
Number of animals tested	5	4	5	5	5	4
Mean	81.5	42.95	7.674	11.99	3.002	5.035
Standard deviation	6.399	18.27	1.308	6.233	0.5512	2.095
Standard error of mean	2.862	9.135	0.585	2.788	0.2465	1.047
P value summary	**		ns		ns	
P value (unpaired t test)	0.003		0.168		0.0723	

Supplementary Figure 6: Flow cytometric analysis of control and AE-expressing mice reveals B and T cell maturation defects

(A) Representative FACS profiles of BM cells from R26/AE reconstituted RAG2$^{-/-}$ mice that have been DOX-induced for nine months are depicted on the left. FACS plots showing B cell maturation were gated on 7AAD^{-} cells and subclassified into pre-pro (B220^{+}/IgM^{-}), immature (B220^{+}/IgM^{low}) and mature (B220^{+}/IgM^{high}) B cell maturation stages. The graph shows relative percentages of control (white squares) and DOX-induced (black dots) cells within the three analysed B cell populations with each square and dot representing one individual mouse. The two tables on the right indicate mean absolute (upper table) and mean relative (lower table) cell percentages, the number of animals that were analysed, standard deviations, standard errors of mean, p value summaries and p values for each maturation stage.

(B) Representative FACS profiles of thymic cells from R26/AE reconstituted RAG2$^{-/-}$ mice that have been DOX-induced for nine months are depicted on the left. FACS plots showing initial T cell maturation were gated on 7AAD^{-}/CD4^{-}/CD8^{-} cells and subclassified into DN1 (CD44^{+}/CD25^{-}), DN2 (CD44^{+}/CD25^{+}) and DN3 (CD44^{-}/CD25^{+}) thymocytes. The graph shows relative percentages of control (white squares) and DOX-induced (black dots) cells within the three analysed T cell populations with each square and dot representing one individual mouse. The two tables on the right indicate mean absolute (upper table) and mean relative (lower table) cell percentages, the number of animals that were analysed, standard deviations, standard errors of mean, p value summaries and p values for each maturation stage.

(C) Representative FACS profiles of thymic cells from R26/AE reconstituted RAG2$^{-/-}$ mice that have been DOX-induced for nine months are depicted on the left. FACS plots showing T cell maturation were gated on 7AAD^{-} cells and subclassified into DP (CD4^{+}/CD8^{+}), CD4 (CD4^{+}/CD8^{-}) and CD8 (CD4^{-}/CD8^{+}). The graph shows relative percentages of control (white squares) and DOX-induced (black dots) cells within the three analysed T cell populations with each square and dot representing one individual mouse. The two tables on the right indicate mean absolute (upper table) and mean relative (lower table) cell percentages, the number of animals that were analysed, standard deviations, standard errors of mean, p value summaries and p values for each maturation stage.

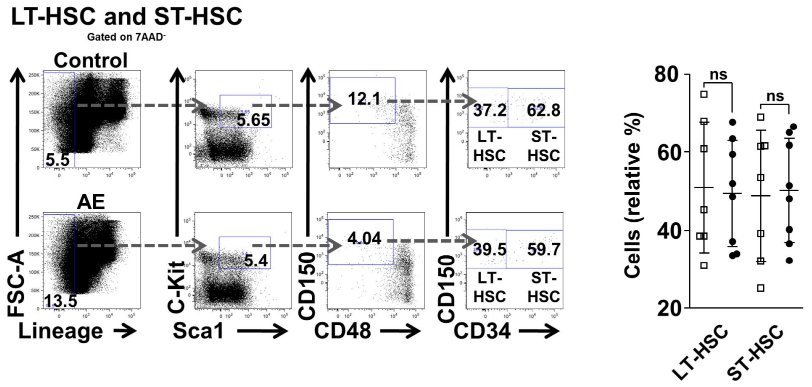

HSC (absolute % of total BM cells)

Population	LT-HSC		ST-HSC	
	Control	AE	Control	AE
Number of animals tested	7	8	7	8
Mean	0.00977	0.01344	0.01576	0.02196
Standard deviation	0.007598	0.009215	0.008721	0.0129
Standard error of mean	0.002872	0.003258	0.003296	0.00456
P value summary	ns		ns	
P value (unpaired t test)	0.4193		0.3031	

HSC (relative % of gated cells)

Population	LT-HSC		ST-HSC	
	Control	AE	Control	AE
Number of animals tested	7	8	7	8
Mean	50.99	49.45	48.84	50.23
Standard deviation	16.79	13.51	16.72	13.26
Standard error of mean	6.344	4.776	6.321	4.688
P value summary	ns		ns	
P value (unpaired t test)	0.8474		0.86111	

Supplementary Figure 7: AE expression does not alter the haematopoietic stem cell compartment

Representative FACS profiles of BM cells from R26/AE reconstituted RAG2$^{-/-}$ mice that have been DOX-induced for nine months are depicted. FACS plots showing LT- and ST-HSC were gated on 7AAD^{-}/L^{-}K^{+}S^{+}/CD48^{-}/CD150^{+} cells and subclassified for the expression of CD34^{-} (LT-HSC) and CD34^{+} (ST-HSC). The graph shows relative percentages of control (white squares) and DOX-induced (black dots) cells within the LT- and ST-HSC populations with each square and dot representing one individual mouse. The two tables on the right indicate, mean absolute (upper table) and mean relative (lower table) cell percentages, the number of animals that were analysed, standard deviations, standard errors of mean, p value summaries and p values for LT- and ST-HSC.

A

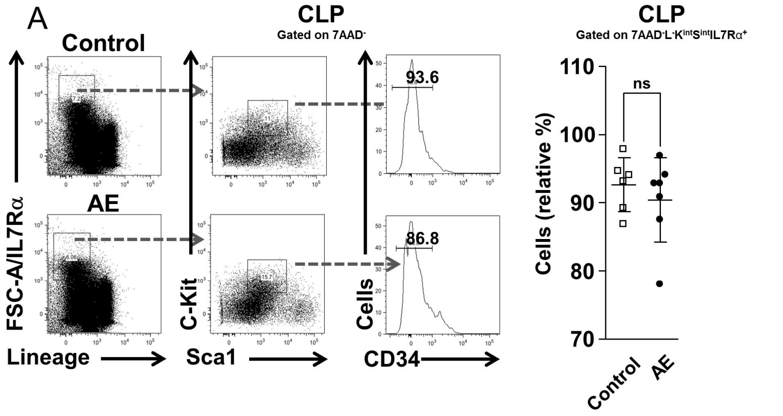

CLP (absolute % of total BM cell

Population	CLP	
	Control	AE
Number of animals tested	7	8
Mean	0.2713	0.2963
Standard deviation	0.1988	0.1225
Standard error of mean	0.07513	0.0433
P value summary	ns	
P value (unpaired t test)	0.7709	

CLP (relative % of gated cells)

Population	CLP	
	Control	AE
Number of animals tested	6	7
Mean	92.6	90.4
Standard deviation	3.932	6.171
Standard error of mean	1.605	2.332
P value summary	ns	
P value (unpaired t test)	0.469	

B

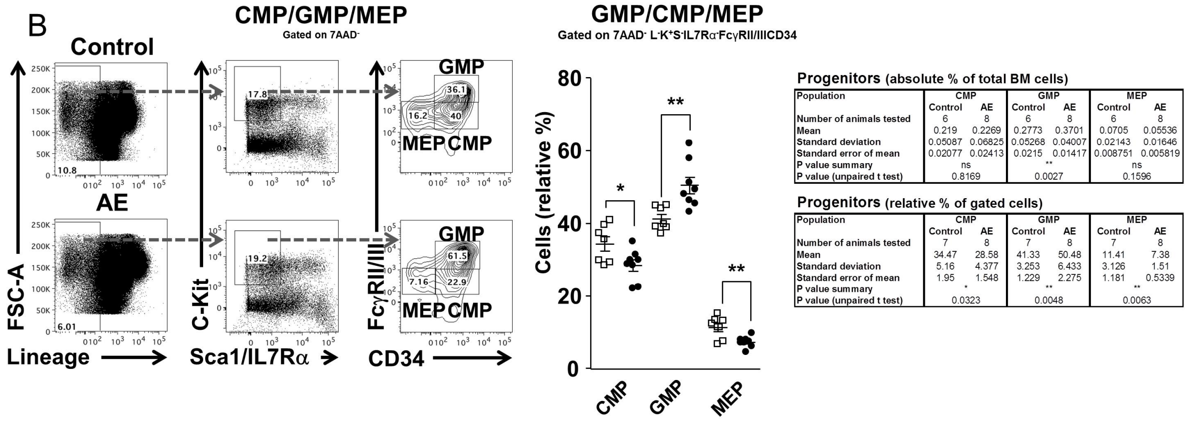

Progenitors (absolute % of total BM cells)

Population	CMP		GMP		MEP	
	Control	AE	Control	AE	Control	AE
Number of animals tested	6	8	6	8	6	8
Mean	0.219	0.2269	0.2773	0.3701	0.0705	0.05536
Standard deviation	0.05087	0.06825	0.05268	0.04007	0.02143	0.01646
Standard error of mean	0.02077	0.02413	0.0215	0.01417	0.008751	0.005819
P value summary	ns		**		ns	
P value (unpaired t test)	0.8169		0.0027		0.1596	

Progenitors (relative % of gated cells)

Population	CMP		GMP		MEP	
	Control	AE	Control	AE	Control	AE
Number of animals tested	7	8	7	8	7	8
Mean	34.47	28.58	41.33	50.48	11.41	7.38
Standard deviation	5.16	4.377	3.253	6.433	3.126	1.51
Standard error of mean	1.95	1.548	1.229	2.275	1.181	0.5339
P value summary	*		**		**	
P value (unpaired t test)	0.0323		0.0048		0.0063	

Supplementary Figure 8: AE expression does not change CLP but alters the CMP, GMP and MEP pools of lineage-restricted progenitors

(A) Representative FACS profiles of BM cells from R26/AE reconstituted RAG2$^{-/-}$ mice that have been DOX-induced for nine months are depicted. FACS plots showing CLP were gated on 7AAD^{-}/L^{-}K^{int}S^{int}/IL7 receptor α^{+}. The graph shows relative percentages of control (white squares) and DOX-induced (black dots) cells within the CLP population with each square and dot representing one individual mouse. The two tables on the right indicate mean absolute (upper table) and mean relative (lower table) cell percentages, the number of animals that were analysed, standard deviations, standard errors of mean, p value summaries and p values for CLP.

(**B**) Representative FACS profiles of BM cells from R26/AE reconstituted RAG2$^{-/-}$ mice that have been DOX-induced for nine months are depicted. GMP, CMP and MEP were gated on 7AAD^{-}/L^{-}K^{+}S^{-}/ IL7 receptor α^{-} expression and subclassified based on the expression of Fcγ receptor and CD34L^{-}K^{int}S^{int}. The graph shows relative percentages of control (white squares) and DOX-induced (black dots) cells within the three analysed progenitor populations with each square and dot representing one individual mouse. The two tables on the right indicate mean absolute (upper table) and mean relative (lower table) cell percentages, the number of animals that were analysed standard deviations, standard errors of mean, p value summaries and p values for each progenitor population.

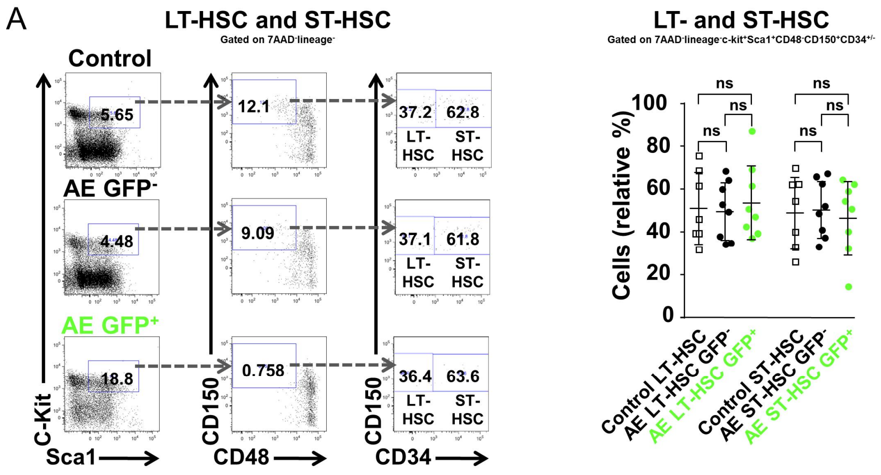

HSC (relative % of gated cells)

Population	LT-HSC GFP-		LT-HSC GFP+	
	Control	AE	Control	AE
Number of animals tested	7	8	7	8
Mean	50.99	49.45	50.99	53.55
Standard deviation	16.79	13.51	16.79	17.21
Standard error of mean	6.344	4.776	6.344	6.086
P value summary	ns		ns	
P value (unpaired t test)	0.8474		0.755	
Population	ST-HSC GFP-		ST-HSC GFP+	
	Control	AE	Control	AE
Number of animals tested	7	8	7	8
Mean	48.84	50.23	48.84	46.45
Standard deviation	16.72	13.26	16.72	17.21
Standard error of mean	6.321	4.688	6.321	6.086
P value summary	ns		ns	
P value (unpaired t test)	0.8611		0.7898	

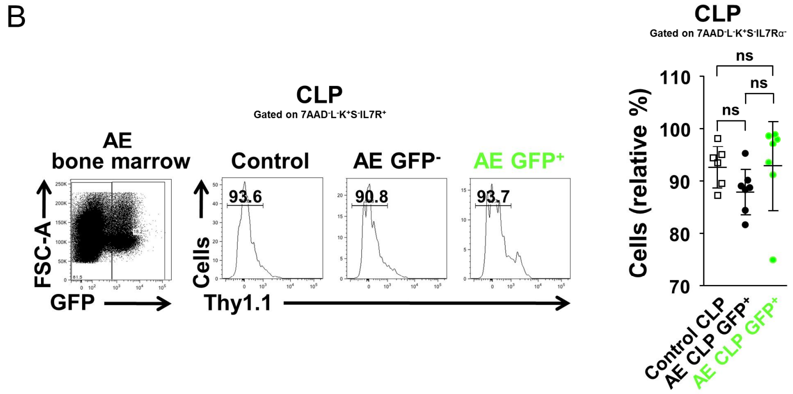

CLP (relative % of gated cells)

Population	CLP GFP-		CLP GFP+	
	Control	AE	Control	AE
Number of animals tested	6	7	6	7
Mean	92.6	87.89	92.6	92.86
Standard deviation	3.932	4.311	3.932	8.512
Standard error of mean	1.605	1.629	1.605	3.217
P value summary	ns		ns	
P value (unpaired t test)	0.0655		0.9472	

Supplementary Figure 9: Analysis of GFP^{-} and GFP^{+} LT-, ST-HSC and CLP populations reveals no significant pool size differences between control and AE-expressing mice

(A) Representative FACS profiles of BM cells from R26/AE reconstituted RAG2$^{-/-}$ mice that have been DOX-induced for nine months are depicted. FACS plots showing LT- and ST-HSC were gated on 7AAD^{-}/L^{-}K^{+}S^{+}/CD48^{-}/CD150^{+} cells and subclassified for the expression of CD34^{-} (LT-HSC), CD34^{+} (ST-HSC) and GFP. The graph shows relative percentages of control (white squares) and DOX-exposed GFP^{-} (black dots) and GFP^{+} (green dots) cells within the LT- and ST-HSC populations with each square and dot representing one individual mouse. The tables at the right show the number of animals that were analysed, mean percentages of control, GFP^{-} and GFP^{+} cells, standard deviations and standard errors of mean, p value summaries and p values for LT-HSC (upper table) and ST-HSC (lower table).

(**B**) Representative FACS profiles of BM cells from R26/AE reconstituted RAG2$^{-/-}$ mice that have been DOX-induced for nine months are depicted. FACS plots showing CLP were gated on 7AAD^{-}/L^{-}K^{int}S^{int}/IL7 receptor α^{+} and subclassified for the expression of GFP. The graph shows relative percentages of control (white squares) and DOX-exposed GFP^{-} (black dots) and GFP^{+} (green dots) cells within the CLP population with each square and dot representing one individual mouse. The table indicates the number of animals that were analysed, mean percentages of control, GFP^{-} and GFP^{+} cells, standard deviations and standard errors of mean, p value summaries and p values for CLP.

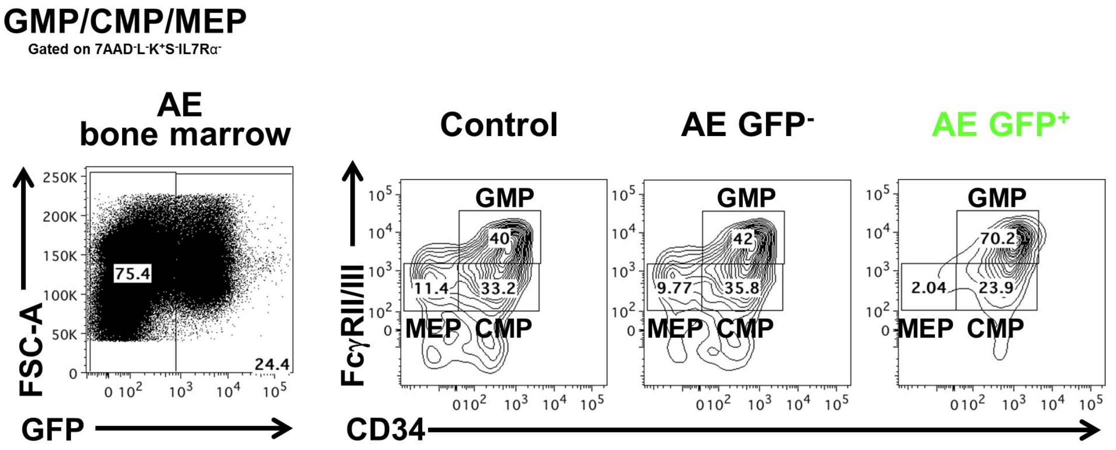

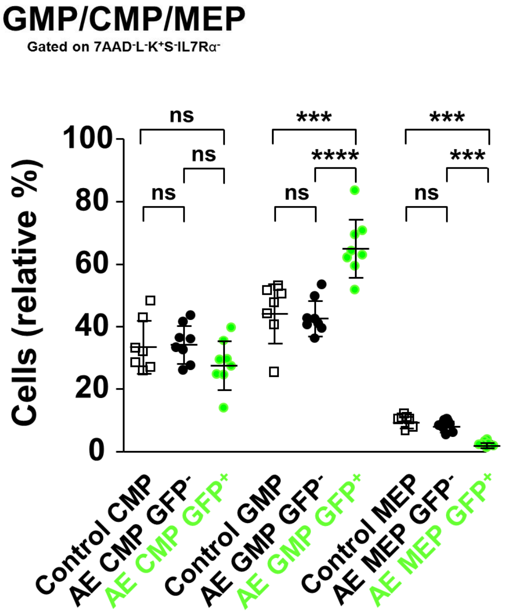

Progenitors (relative % of gated cells)

Population	CMP GFP-		CMP GFP+	
	Control	AE	Control	AE
Number of animals tested	7	8	7	8
Mean	33.33	34.03	33.33	27.5
Standard deviation	8.513	6.177	8.513	7.753
Standard error of mean	3.217	2.184	3.217	2.741
P value summary	ns		ns	
P value (unpaired t test)	0.8575		0.1884	
**Population**	**GMP GFP-**		**GMP GFP+**	
	Control	AE	Control	AE
Number of animals tested	7	8	7	8
Mean	44.16	42.7	44.16	65.03
Standard deviation	9.56	5.618	9.56	9.321
Standard error of mean	3.613	1.986	3.613	3.295
P value summary	ns		***	
P value (unpaired t test)	0.7203		0.0009	
**Population**	**MEP GFP-**		**MEP GFP+**	
	Control	AE	Control	AE
Number of animals tested	7	8	7	8
Mean	9.213	7.845	9.213	1.862
Standard deviation	1.919	1.789	1.919	0.7938
Standard error of mean	0.7252	0.6326	0.7252	0.2806
P value summary	ns		***	
P value (unpaired t test)	0.1767		<0.0001	

Supplementary Figure 10: Relative percentages of GFP^{+} but not GFP^{-} GMP and MEP pools are altered in AE-expressing mice

Representative FACS profiles of BM cells from R26/AE reconstituted RAG2$^{-/-}$ mice that have been DOX-induced for nine months are depicted. The first plot shows the percentages of GFP^{-} and GFP^{+} cells in the BM of a DOX-induced mouse. In the other plots, control, DOX-exposed GFP^{-} and GFP^{+} GMP, CMP and MEP are shown that were gated on 7AAD^{-}/L^{-}K^{+}S^{-}/ IL7 receptor α^{-} expression and subclassified based on the expression of Fcγ receptor, CD34 and GFP. The graph at the left bottom shows relative percentages of control (white squares), DOX-exposed GFP^{-} (black dots) and GFP^{+} (green dots) cells within each progenitor population with each square and dot representing one individual mouse. The tables indicate the number of animals that were analysed, mean relative percentages of control, GFP^{-} and GFP^{+} cells, standard deviations and standard errors of mean, p value summaries and p values for CMP, GMP and MEP.

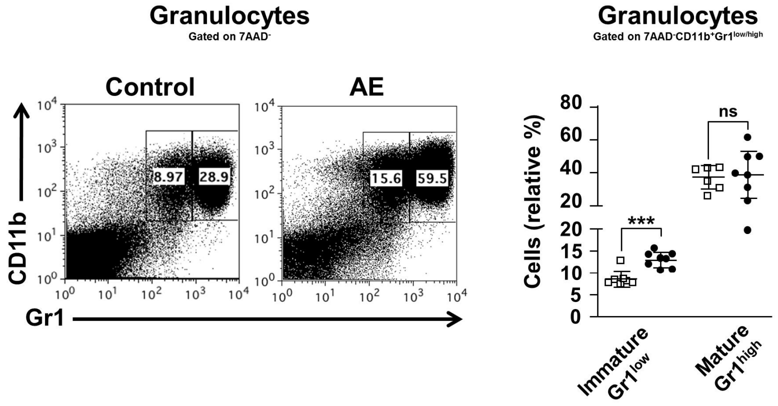

Granulocytes (absolute % of total BM cells)

Population	Immature		Mature	
	Control	AE	Control	AE
Number of animals tested	6	8	7	8
Mean	7.467	13.64	23.67	31.19
Standard deviation	0.9413	5.615	6.796	14.47
Standard error of mean	0.3843	1.985	2.569	5.117
P value summary	*		ns	
P value (unpaired t test)	0.0216		0.2319	

Granulocytes (relative % of gated cells)

Population	Immature		Mature	
	Control	AE	Control	AE
Number of animals tested	7	8	7	8
Mean	8.591	12.93	37.23	38.73
Standard deviation	1.858	1.752	7.056	14.18
Standard error of mean	0.7022	0.6195	2.667	5.014
P value summary	***		ns	
P value (unpaired t test)	0.0005		0.8047	

S

Supplementary Figure 11: Immature granulocytes are increased in the BM of long-term AE-expressing mice

Representative FACS profiles of BM cells from R26/AE reconstituted C57BL/6 mice that have been DOX-induced for eighteen months are depicted on the left. FACS plots showing granulocytes were gated on $7AAD^-$ cells and subclassified into immature ($CD11b^+Gr1^{low}$) and mature ($CD11b^+Gr1^{high}$) stages. The graph shows relative percentages of control (white squares) and DOX-induced (black dots) cells within the two analysed granulocytic populations with each square and dot representing one individual mouse. The two tables on the right indicate mean absolute (upper table) and mean relative (lower table) cell percentages, the number of animals that were analysed, standard deviations, standard errors of mean, p value summaries and p values for each maturation stage.

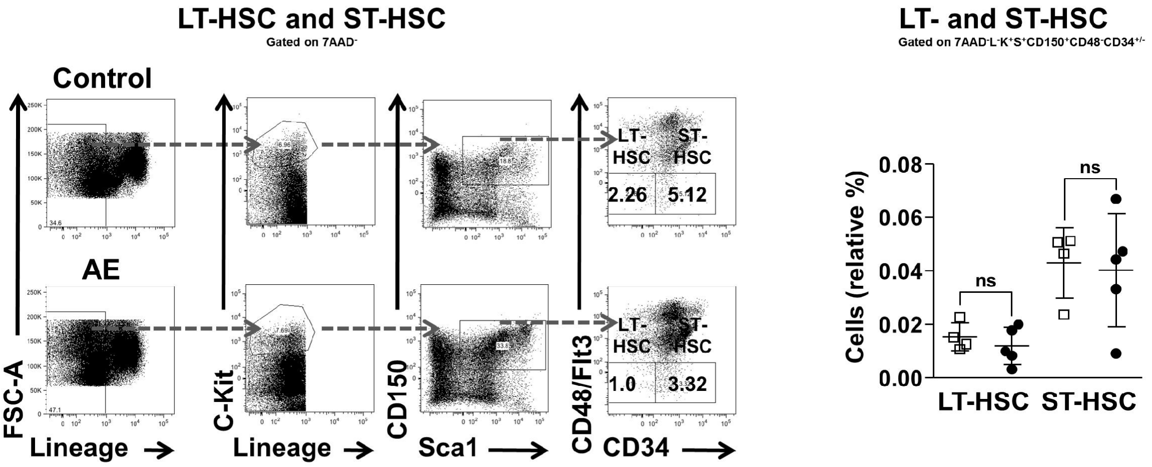

HSC (absolute % of total BM cells)

Population	LT-HSC		ST-HSC	
	Control	AE	Control	AE
Number of animals tested	3	5	3	5
Mean	0.01187	0.01034	0.03633	0.04342
Standard deviation	0.002214	0.006279	0.01146	0.02472
Standard error of mean	0.001278	0.002808	0.006615	0.01105
P value summary	ns		ns	
P value (unpaired t test)	0.7064		0.6638	

HSC (relative % of gated cells)

Population	LT-HSC		ST-HSC	
	Control	AE	Control	AE
Number of animals tested	4	5	4	5
Mean	1.525	1.187	4.295	4.011
Standard deviation	0.5221	0.6928	1.308	2.114
Standard error of mean	0.261	0.3098	0.6538	0.9452
P value summary	ns		ns	
P value (unpaired t test)	0.4469		0.8218	

Supplementary Figure 12: Long-term AE expression does not alter the pool of LT- and ST-HSC in leukaemic mice

Representative FACS profiles of BM cells from R26/AE reconstituted C57BL/6 mice that have been DOX-induced for eighteen months are depicted. FACS plots showing LT- and ST-HSC were gated on $7AAD^{-}/L^{-}K^{+}S^{+}/CD48^{-}/Flt3^{-}/CD150^{+}$ cells and subclassified for the expression of $CD34^{-}$ (LT-HSC) and $CD34^{+}$ (ST-HSC). The graph on the right shows relative percentages of control (white squares) and DOX-induced (black dots) cells within the LT- and ST-HSC populations with each square and dot representing one individual mouse. The two tables on the right indicate mean absolute (upper table) and mean relative (lower table) cell percentages, the number of animals that were analysed, standard deviations, standard errors of mean, p value summaries and p values for LT- and ST-HSC.

A

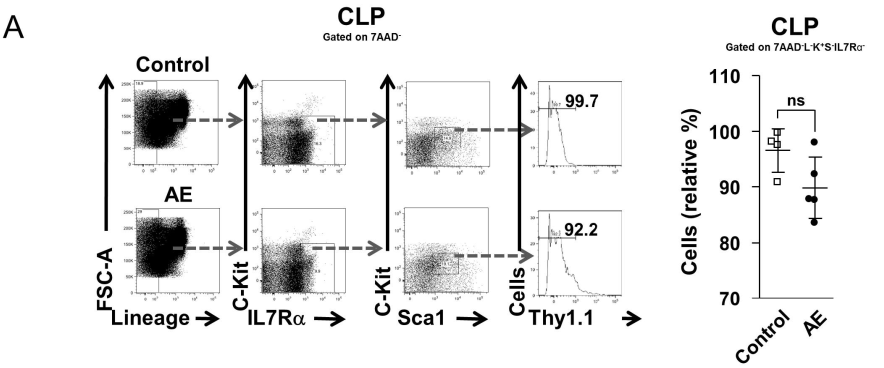

CLP (absolute % of total BM cells)

Population	CLP	
	Control	AE
Number of animals tested	4	5
Mean	0.4198	0.1746
Standard deviation	0.3329	0.08363
Standard error of mean	0.1664	0.0374
P value summary	ns	
P value (unpaired t test)	0.1513	

CLP (relative % of gated cells)

Population	CLP	
	Control	AE
Number of animals tested	4	5
Mean	96.55	89.82
Standard deviation	3.942	5.512
Standard error of mean	1.971	2.465
P value summary	ns	
P value (unpaired t test)	0.0799	

B

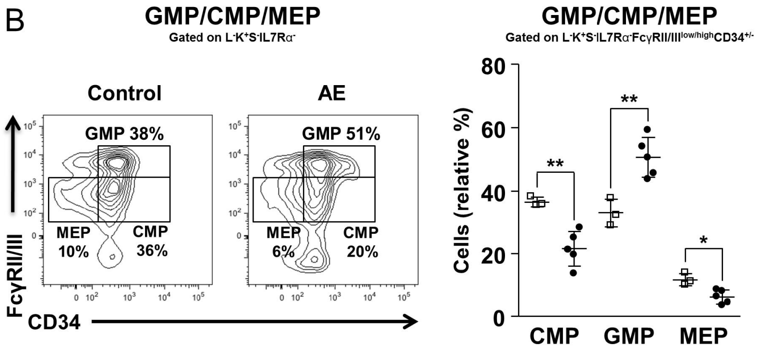

Progenitors (absolute % of total BM cells)

Population	CMP		GMP		MEP	
	Control	AE	Control	AE	Control	AE
Number of animals tested	3	4	3	4	3	4
Mean	0.3613	0.2985	0.3267	0.718	0.1137	0.09295
Standard deviation	0.05401	0.1038	0.06786	0.1463	0.01185	0.03104
Standard error of mean	0.03118	0.05191	0.03918	0.07314	0.006839	0.01552
P value summary	ns		**		ns	
P value (unpaired t test)	0.3897		0.0083		0.3306	

Progenitors (relative % of gated cells)

Population	CMP		GMP		MEP	
	Control	AE	Control	AE	Control	AE
Number of animals tested	3	5	3	5	3	5
Mean	36.6	21.56	33	50.64	11.63	6.122
Standard deviation	1.48	5.573	4.491	6.26	1.955	2.22
Standard error of mean	0.8544	2.492	2.593	2.8	1.129	0.9927
P value summary	**		**		*	
P value (unpaired t test)	0.0043		0.0056		0.0123	

Supplementary Figure 13: AE expression alters the relative percentages of CMP, GMP and MEP

(A) Representative FACS profiles of BM cells from R26/AE reconstituted C57BL/6 mice that have been DOX-induced for eighteen months are depicted. FACS plots showing CLP were gated on $7AAD^{-}/L^{-}K^{int}S^{int}$/IL7 receptor α^{+} and subclassified for the expression of GFP. The graph shows relative percentages of control (white squares) and DOX-induced (black dots) cells within the CLP population with each square and dot representing one individual mouse. The two tables on the right indicate mean absolute (upper table) and mean relative (lower table) cell percentages, the number of animals that were analysed, standard deviations, standard errors of mean, p value summaries and p values for CLP.

(**B**) Representative FACS profiles of BM cells from R26/AE reconstituted C57BL/6 mice that have been DOX-induced for eighteen months are depicted. GMP, CMP and MEP were gated on $7AAD^{-}/L^{-}K^{+}S^{-}$/ IL7 receptor α^{-} expression and subclassified based on the expression of Fcγ receptor, CD34 and GFP. The graph shows relative percentages of control (white squares) and DOX-induced (black dots) cells within the three analysed progenitor populations with each square and dot representing one individual mouse. The two tables on the right indicate mean absolute (upper table) and mean relative (lower table) cell percentages, the number of animals that were analysed, standard deviations, standard errors of mean, p value summaries and p values for each maturation stage.

A

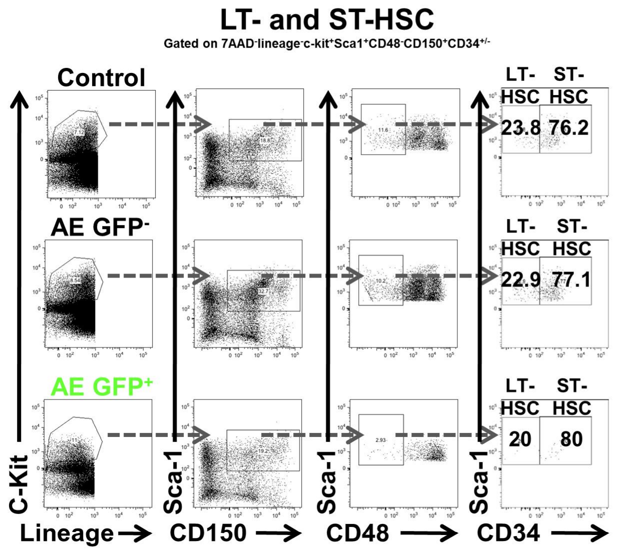

HSC (relative % of gated cells)

Population	LT-HSC GFP-		LT-HSC GFP+	
	Control	AE	Control	AE
Number of animals tested	4	5	4	5
Mean	19.93	18.04	19.93	14
Standard deviation	5.386	3.702	5.386	12.94
Standard error of mean	2.693	1.655	2.693	5.788
P value summary	ns		ns	
P value (unpaired t test)	0.5523		0.4238	
Population	ST-HSC GFP-		ST-HSC GFP+	
	Control	AE	Control	AE
Number of animals tested	4	5	4	5
Mean	79.03	81.56	79.03	46
Standard deviation	5.261	3.623	5.261	42.04
Standard error of mean	2.631	1.62	2.631	18.8
P value summary	ns		ns	
P value (unpaired t test)	0.4189		0.1674	

B

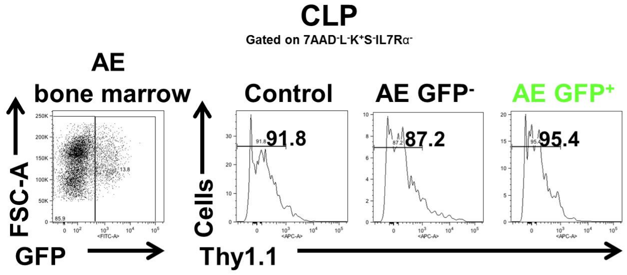

CLP (relative % of gated cells)

Population	CLP GFP-		CLP GFP+	
	Control	AE	Control	AE
Number of animals tested	4	5	4	5
Mean	96.95	90.74	96.95	85.3
Standard deviation	3.486	5.902	3.486	8.43
Standard error of mean	1.743	2.639	1.743	3.77
P value summary	ns		*	
P value (unpaired t test)	0.1072		0.0372	

Supplementary Figure 14: Analysis of GFP^{-} and GFP^{+} LT-, ST-HSC and CLP populations reveals no significant differences between control and leukaemic mice

(A) Representative FACS profiles of BM cells from R26/AE reconstituted C57BL/6 mice that have been DOX-induced for eighteen months are depicted. FACS plots showing LT- and ST-HSC were gated on 7AAD^{-}/L^{-}K^{+}S^{+}/CD48^{-}/CD150^{+} cells and subclassified for the expression of CD34^{-} (LT-HSC), CD34^{+} (ST-HSC) and GFP. The tables at the right show the number of animals that were analysed, mean percentages of control, GFP^{-} and GFP^{+} cells, standard deviations, standard errors of mean, p value summaries and p values for LT-HSC (upper table) and ST-HSC (lower table).

(**B**) Representative FACS profiles of BM cells from R26/AE reconstituted C57BL/6 mice that have been DOX-induced for nine months are depicted. FACS plots showing CLP were gated on 7AAD^{-}/L^{-}K^{int}S^{int}/IL7 receptor α^{+} and subclassified for the expression of GFP. The table indicates the number of animals that were analysed, mean percentages of control, GFP^{-} and GFP^{+} cells, standard deviations and standard errors of mean, p value summaries and p values for CLP.

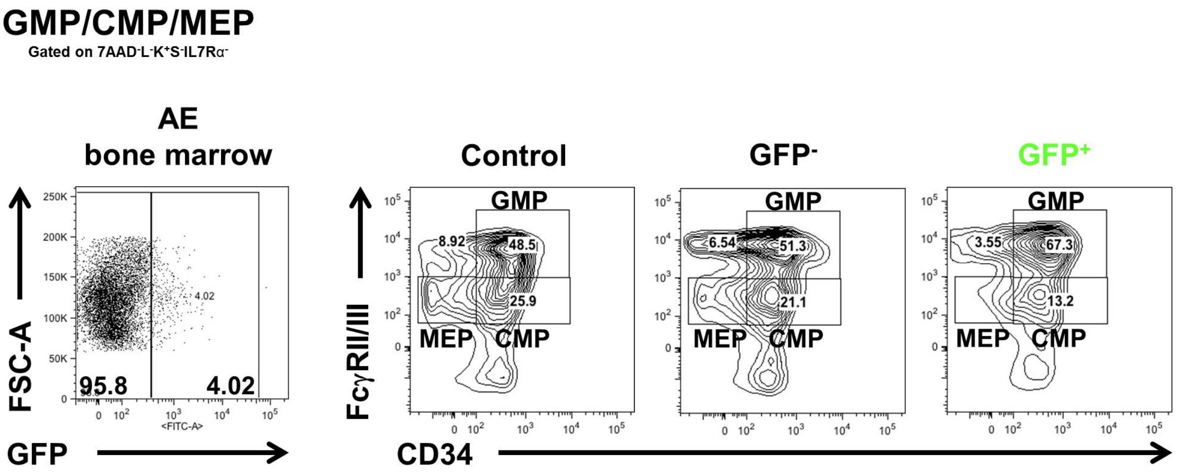

Progenitors (relative % of gated cells)

Population	CMP GFP-		CMP GFP+	
	Control	AE	Control	AE
Number of animals tested	3	5	3	5
Mean	25.43	16.89	25.43	13.8
Standard deviation	4.801	5.409	4.801	1.442
Standard error of mean	2.772	2.419	2.772	0.645
P value summary	ns		**	
P value (unpaired t test)	0.0661		0.0018	
**Population**	**GMP GFP-**		**GMP GFP+**	
	Control	AE	Control	AE
Number of animals tested	3	5	3	5
Mean	46.33	52.82	46.33	66.42
Standard deviation	8.501	5.329	8.501	6.212
Standard error of mean	4.908	2.383	4.908	2.778
P value summary	ns		**	
P value (unpaired t test)	0.2244		0.008	
**Population**	**MEP GFP-**		**MEP GFP+**	
	Control	AE	Control	AE
Number of animals tested	3	5	3	5
Mean	8.237	5.052	8.237	4.076
Standard deviation	2.196	1.962	2.196	1.732
Standard error of mean	1.268	0.8776	1.268	0.7747
P value summary	ns		*	
P value (unpaired t test)	0.0767		0.024	

Supplementary Figure 15: Relative percentages of GFP$^+$ but not GFP$^-$ CMP, GMP and MEP pools are altered in leukaemic mice

Representative FACS profiles of BM cells from R26/AE reconstituted C57BL/6 mice that have been DOX-induced for eighteen months are depicted. The first plot shows GFP$^-$ and GFP$^+$ cells in the BM of a leukaemic mouse with numbers indicating the percentages of GFP$^-$ and GFP$^+$ cells within the population of nucleated BM cells. In the contour plots, control, DOX-exposed GFP$^-$ and GFP$^+$ GMP, CMP and MEP are shown that were gated on 7AAD$^-$/L$^-$K$^+$S$^-$/ IL7 receptor α^- expression and subclassified based on the expression of Fcγ receptor, CD34 and GFP. The table below indicates the number of animals that were analysed, mean relative percentages of control, GFP$^-$ and GFP$^+$ cells, standard deviations, standard errors of mean, p value summaries and p values for CMP (upper table), GMP (table in the middle) and MEP (lower table).

A

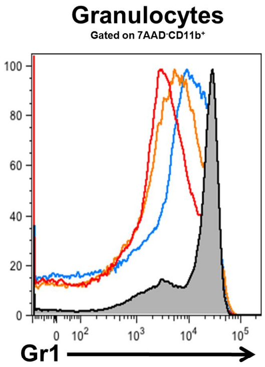

Granulocytes (relative % of GFP+/Gr1+ cells)

Population	Immature granulocytes (GFP+/Gr1 low)			
	Control	+DOX	-DOX	-DOX
Relative %	11.2	25.2	24.2	13.7

Population	Mature granulocytes (GFP+/Gr1 high)			
	Control	+DOX	-DOX	-DOX
Relative %	42.6	16.7	29	33.5

B

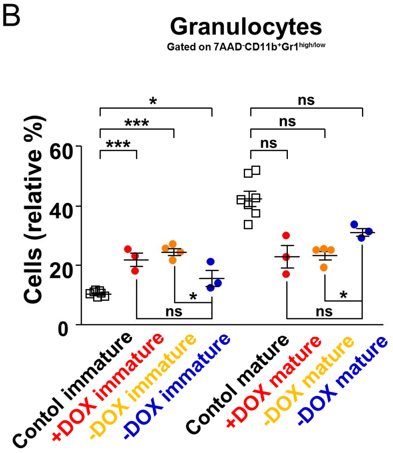

Granulocytes (absolute % of total cells)

Population	Immature Granulocytes									
	Control	+DOX	Control	-DOX	Control	-DOX	+DOX	-DOX	-DOX	-DOX
Number of animals tested	7	3	7	4	7	3	3	3	4	3
Mean	5.63	17.83	5,630	19,73	5,630	12,50	17.83	12,50	19,73	12,50
Standard deviation	2.001	3.722	2,001	1,793	2,001	4,118	3.722	4,118	1,793	4,118
Standard error of mean	0.7563	2.149	0,7563	0,8966	0,7563	2,378	2.149	2,378	0,8966	2,378
P value summary	***		***		**		ns		*	
P value (unpaired t test)	0.0001		<0.0001		0.006		0.1716		0.0239	

Population	Mature Granulocytes									
	Control	+DOX	Control	-DOX	Control	-DOX	-DOX	-DOX	-DOX	-DOX
Number of animals tested	7	3	7	4	7	3	3	3	4	3
Mean	24,36	18,50	24,36	18,90	24,36	24,70	18,50	24,70	18,90	24,70
Standard deviation	11,97	4,976	11,97	2,135	11,97	1,473	4,976	1,473	2,135	1,473
Standard error of mean	4,523	2,873	4,523	1,068	4,523	0,8505	2,873	0,8505	1,068	0,8505
P value summary	ns		ns		ns		ns		*	
P value (unpaired t test)	0.4488		0.3996		0.963		0.1073		0.0103	

Granulocytes (relative % of gated cells)

Population	Immature Granulocytes									
	Control	+DOX	Control	-DOX	Control	-DOX	+DOX	-DOX	-DOX	-DOX
Number of animals tested	7	3	7	4	7	3	3	3	4	3
Mean	10,38	22,07	10,38	24,63	10,38	15,70	22,07	15,70	24,63	15,70
Standard deviation	0,9067	3,787	0,9067	2,281	0,9067	4,744	3,787	4,744	2,281	4,744
Standard error of mean	0,3427	2,187	0,3427	1,140	0,3427	2,739	2,187	2,739	1,140	2,739
P value summary	***		***		*		ns		*	
P value (unpaired t test)	<0.0001		<0.0001		0.015		0.1435		0.0202	

Population	Mature Granulocytes									
	Control	+DOX	Control	-DOX	Control	-DOX	+DOX	-DOX	-DOX	-DOX
Number of animals tested	7	3	7	4	7	3	3	3	4	3
Mean	42,93	23,13	42,93	23,50	42,93	31,37	23,13	31,37	23,50	31,37
Standard deviation	6,755	6,616	6,755	2,934	6,755	2,301	6,616	2,301	2,934	2,301
Standard error of mean	2,553	3,820	2,553	1,467	2,553	1,328	3,820	1,328	1,467	1,328
P value summary	**		***		*		ns		*	
P value (unpaired t test)	0.0027		0.0004		0.0228		0.1115		0.0124	

Supplementary Figure 16: Flow cytometric analysis of BM granulocytes reveals the reduction of immature myeloid cells in two previously leukaemic mice following the DOX switch

(A) Histogram plots of Gr1-expressing BM granulocytes for a control animal (black outline and filled in grey), a mouse continuously receiving DOX (+DOX, red line), a mouse shifted to a DOX-free diet presenting signs of progressing leukaemia (−DOX, orange line) and an animal that has been shifted to a DOX-free diet presenting signs of recovery and partial restoration of granulocytic maturation (−DOX, blue line). Note the right shift of the blue profile indicating the reduction of immature $Gr1^{low}$ and the increase of $Gr1^{high}$ cells. The table on the right indicates the relative percentages of GFP^+ mature and immature granulocytes for each individual mouse that is shown in the histogram.

(**B**) The graph on the left shows relative percentages of immature ($Gr1^{low}$) and more mature ($Gr1^{high}$) cells within the BM population of granulocytes for control mice (white squares), animals continuously receiving DOX (+DOX, red dots), animals that had been shifted to a DOX-free diet presenting signs of progressing leukaemia (−DOX, orange dots) and mice that had been shifted to a DOX-free diet presenting signs of recovery (−DOX, blue dots). The tables on the right indicate the mean absolute and relative percentages, the number of animals that were analysed, standard deviations, standard errors of mean, p value summaries and p values for all immature (upper tables) and mature granulocytes (lower tables).

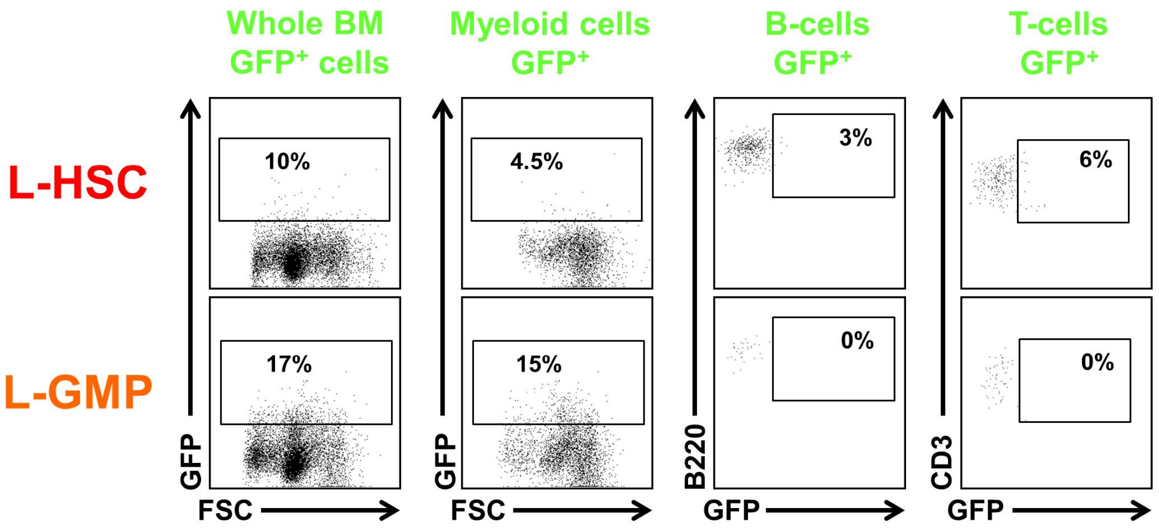

Supplementary Figure 17: Post reconstitution flow cytometric analysis demonstrates that L-HSC reconstitute both lympho- and myelopoiesis whereas L-GMP only reconstitute myelopoiesis

The upper panel shows representative FACS plots for an L-HSC- and the lower panel for an L-GMP-reconstituted mouse. Numbers indicate the percentages of GFP^+ cells in the BM (left plots) or in the myeloid, B and T cell fraction of peripheral blood samples. Note the absence of GFP-expressing T and B cells in the L-GMP-reconstituted mouse.

A

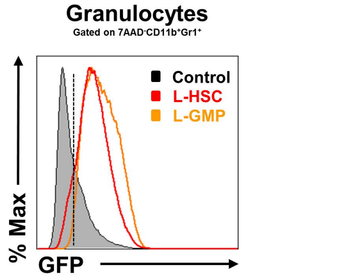

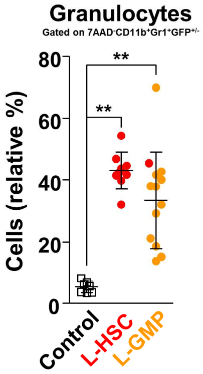

Granulocytes (relative % of GFP+/CD11b+/Gr1+ cells)

Population	Granulocytes (GFP+/CD11b+/Gr1+)		
	Control	L-HSC	L-GMP
Relative %	3.73	25.2	24.2

L-HSC-derived granulocytes (relative % of gated cells)

Population	Control	L-HSC
Gr1/Cd11b	+	+
GFP	-	+
Number of animals tested	7	9
Mean percentage	5.399	43
Standard deviation	1.881	5.923
Standard error of mean	0.7111	1.974
P value summary	***	
P value (unpaired t test)	<0.0001	

L-GMP-derived granulocytes (relative % of gated cells)

Population	Control	L-GMP
Gr1/Cd11b	+	+
GFP	-	+
Number of animals tested	7	12
Mean percentage	5.399	33.38
Standard deviation	1.881	15.6
Standard error of mean	0.7111	4.504
P value summary	***	
P value (unpaired t test)	0.0002	

B

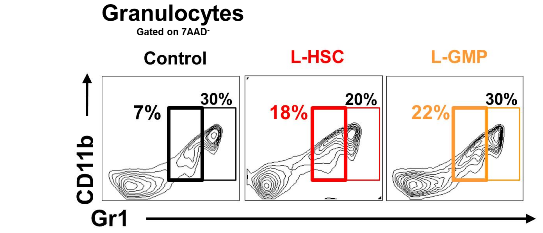

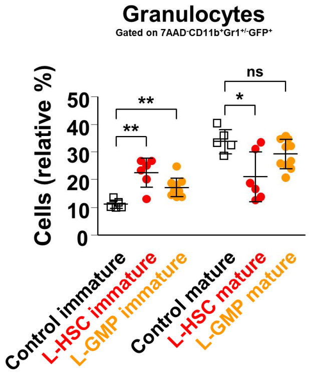

Granulocytes (absolute % of total cells)

Population	Immature granulocytes		Immature granulocytes		Mature granulocytes		Mature granulocytes	
	Control	L-HSC	Control	L-GMP	Control	L-HSC	Control	L-GMP
Number of animals tested	7	6	7	9	7	6	7	9
Mean	7.564	17.57	7.564	8.963	25.01	14.13	25.01	19.44
Standard deviation	3.247	7.699	3.247	2.247	11.4	2.031	11.4	8.934
Standard error of mean	1.227	3.143	1.227	0.7492	4.31	0.8293	4.31	2.978
P value summary	**		ns		*		ns	
P value (unpaired t test)	0.0093		0.3249		0.0426		0.29	

Granulocytes (relative % of gated cells)

Population	Immature granulocytes		Immature granulocytes		Mature granulocytes		Mature granulocytes	
	Control	L-HSC	Control	L-GMP	Control	L-HSC	Control	L-GMP
Number of animals tested	5	6	5	10	5	6	5	10
Mean	11.16	22.53	11.16	17.17	33.82	21.1	33.82	29.25
Standard deviation	1.491	5.225	1.491	3.32	4.447	8.972	4.447	5.3
Standard error of mean	0.6668	2.133	0.6668	1.05	1.989	3.663	1.989	1.676
P value summary	**		**		*		ns	
P value (unpaired t test)	0.0012		0.0022		0.0184		0.1226	

Supplementary Figure 18: Flow cytometric analysis reveals differences in granulocytic maturation between L-HSC- and L-GMP-reconstituted mice

(A) Histogram plots of CD11b/Gr1-expressing granulocytes for a control animal (black outline and filled in grey), an L-HSC- (red line) and an L-GMP- (orange line) reconstituted mouse revealing the presence of GFP-expressing granulocytes in the BM of reconstituted mice. The table below indicates the relative percentages of GFP+ granulocytes for each individual mouse that is shown in the histogram. The graph on the right shows increased relative percentages of $CD11b^+/Gr1^+$ BM granulocytes in both L-HSC- (red dots) and L-GMP- (orange dots) reconstituted mice when compared to control mice (white squares). The two tables on the right indicate the mean relative cell percentages, number of animals that were analysed, standard deviations, standard errors of mean, p value summaries and p values for L-HSC-reconstituted (upper table) and L-GMP- (lower table) reconstituted animals.
(B) Contour plots depicting immature ($CD11b^+Gr1^{low}$) and mature ($CD11b^+Gr1^{high}$) granulocytes of control, L-HSC- and L-GMP-transplanted mice. Thick-framed boxes highlight immature granulocytes. The graph on the right shows relative percentages of cells for control (white squares), L-HSC- (red dots) and L-GMP- (orange dots) transplanted mice within the immature and mature granulocytic population with each square and dot representing one individual mouse. The two tables at the bottom indicate the mean absolute (upper table) and mean relative (lower table) cell percentages, the number of animals that were analysed, standard deviations, standard errors of mean, p value summaries and p values for control L-HSC- and L-GMP-transplanted mice. Note that immature $CD11b^+/Gr1^{low}$ granulocytes are augmented in both L-HSC and L-GMP-transplanted mice whereas the percentage of more mature $CD11b^+/Gr1^{high}$ cells is only significantly decreased in L-HSC-reconstituted animals but not in L-GMP-transplanted mice.

INFORMATION ABOUT SUPPLEMENTARY TABLES

Table S1 and **Table S2:** Whole transcriptome and mRNA profiles of Ctrl-, ST- and L-GMP populations are provided in Table S1 and Table S2.

Table S3: Table S3 contains lists of transcripts that were specifically increased, further up-regulated, newly transcribed, specifically down-regulated, further decreased or completely extinguished in L-GMP cells.

Table S4: Table S4 contains the list of human orthologous genes corresponding to the top 150 transcripts that were highly up-regulated or exclusively expressed in murine ST-GMP and in murine L-GMP indicating the rank in the gene list, the metric score and the individual enrichment score found in a cohort of 61 t(8;21) CBF AML patients.

Table S5: Table S5 contains the list of 17 human genes that were found to be on average two fold or more increased in a group of 61 t(8;21) patients as compared to a cohort of 69 healthy donors. In addition, these genes were silent or only very poorly transcribed in sorted primary human CD34+/CD38- cord blood HSC, CD34+/CD38low/CD36- cord blood HSC, primary human CD34+/CD38+ haematopoietic progenitors and unsorted Lin+/- human primary cord blood cells.

MATERIALS AND METHODS

Flow cytometry analysis and sorting

For staining of differentiated blood cell lineages we used antibodies against CD4, CD8, CD25, CD44, B220, IgM, Gr1, CD11b, Ter119, CD71, CD41 and CD3. Erythrocytes were removed by lysis with ACK buffer (Lonza) for 5 minutes at room temperature. Stem cells and progenitors were stained with a lineage cocktail containing antibodies against CD3, B220, CD11b, TER119 and Ly-6G followed by staining with antibodies against FcγRII/III, IL7Rα, CD34, CD150, c-Kit, Sca-1, CD48 and/or Flt-3. A list of all antibodies that were used in this study is shown below.

Antibodies for detecting mature hematopoietic lineage markers

Antigen	Fluorochrome	Company	Alternative Name	Clone
B220	PE	BD	CD45R	RA3-6B2
B220	APC	BD	CD45R	RA3-6B2
CD3	PE	Caltag		145-2C11
CD4	PE	BD	L3T4	GK1.5
CD8	PE	BD	Ly-2	53-6.7
CD8	APC	BD	Ly-2	53-6.7
CD11b	APC	BD	Mac-1; Integrin α_M	M1/70
CD11b	PE	BD	Mac-1; Integrin α_M	M1/70
CD19	PE	BD		1D3
CD25	PE-Cy7	BD		PC61
CD41	PE	BD		MWReg30
CD44	APC	BD	Pgp-1, H-CAM, Ly-24	IM7

CD71	Biotin	Caltag	Transferrin R	R17 217.1.4
Gr1	PE	BD	Ly-6C/ -6G	RB6-8C5
IgM	APC	BD		II/41
TER119	PE	BD	Ly-76	TER119

Antibodies for detecting stem and progenitor cells

Antigen	Fluorochrome	Company	Altern. Name	Clone
CD34	PE	Caltag		RAM34
CD48	APC	Natutec	BCM1	HM48-1
CD117	APC	BD	c-Kit	2B8
CD117	APC-Alexa750	Natutec	c-Kit	2B8
CD135	PE-Cy5	Natutec	Flk-2, Flt-3, Ly-72	A2F10
CD150	PE-Cy7	Biozol	SLAM-1	TC15-12,F12.2
FcγRII/III	APC	BD	CD16/32	2.4G2
IL7Rα	PE-Cy7	BD	CD127	A7R34
Sca-1	PE	Caltag	Ly.6A/E	D7
Sca-1	PE-Cy7	eBioscience	Ly.6A/E	D7
Sca-1	PerCp-Cy5.5	eBioscience	Ly.6A/E	D7

Lineage cocktails

Antigen	Fluorochrome	Company	Composition	Clone
Lineage cocktail	Pacific Blue	Natutec	CD3	17A2
			CD45R/B220	RA3-6B2
			CD11b	M1/70
			TER-119	TER-119
			Ly-6G	RB6-8C5

Quantitative real-time PCR

The Kasumi-1 cell line (Asou et al, 1991) was cultivated in RPMI 1640 (GIBCO BRL) containing 10% fetal bovine serum. RNA was extracted from BM of non-induced and induced (three days DOX) compound R26/AE mice. $L^{-}K^{+}S^{+}$ cells (lineage PE-conjugated B220, CD41, CD3, Gr1, CD11c, CD11b and Ter119; APC c-Kit and PE-Cy7 Sca-1 conjugated) were sorted using a FACSVantage Cell Sorter (BD) from whole BM without gating on GFP^{+} cells. For the isolation of differentiated blood cell lineages, anti-PE microbeads recognizing PE-conjugated B220, CD3, Gr1, CD11b or Ter119 (Milteny Biotec*)* were used. Purity of the isolated cells was in all cases > 95%. RNA extraction and DNase I treatment was performed with the RNeasy Mini Kit (Qiagen). RNA samples were reverse-transcribed with SuperScript II (Invitrogen) according to the manufacturer's instructions. Real-time PCR for detecting AE mRNA levels was performed on a Mastercycler Realplex4 (Eppendorf) using the ABsolute qPCR SYBR Green Mix (ABgene). Each sample was run in triplicate and three independent experiments were performed in each case. Non-specific DNA amplification was excluded by comparing experiments conducted in the presence and absence of reverse transcriptase.

Differences in cDNA input between samples were compensated by normalizing to the expression of hprt (ΔC_t). The fold-change value between each test sample was calculated according to Schmittgen (Schmittgen, 2001), calibrated relative to the amount of mRNA in the Kasumi-1 cell line and displayed as $2^{-\Delta\Delta Ct}$. Primers and cycling details are shown below.

QPCR primers

AML1-ETO forward primer	5'GCCCCGAGAACCTCGAAA3'
AML1-ETO reverse primer	5'TCCACACGTGAGTCTGGCATT3'
hprt forward primer	5'GCTCGAGATGTCATGAAGGAGAT3'
hprt reverse primer	5'AGCAGGTCAGCAAAGAACTTATAGC3'

QPCR cycling conditions

Thermal cycling			
		15 minutes	95°C
	40 cycles	15 seconds	95°C
		30 seconds	55°C
		30 seconds	72°C
Melting curve			
		30 seconds	95°C
		30 seconds	60°C
		15 seconds	95°C

BM transplantation for the analysis of phenotypes following short- and long-term DOX induction

BM cells were obtained from non-induced compound R26/AE donors. For the analysis of mice that were exposed to DOX for 8-10 months, 5 x 10^5 whole BM donor cells were injected into the tail vein of irradiated RAG2$^{-/-}$animals (4.5 Gy). For long-term DOX-induction experiments (DOX exposure 16-18 months) involving peripheral blood films, spleen weight, FACS analysis, histology of spleen, thymus, lymph nodes, liver, kidney and lung we used C57BL/6 recipients (irradiation 2 x 5.5 Gy) that were tail vein injected with 5x10^5 donor cells

from non-induced compound R26/AE mice and DOX-exposed for either 16 months (N=5) or 18 months (N=4). Spinal cord sections for histo-pathological analysis (Figure 3D) were obtained from RAG2$^{-/-}$ reconstituted recipients (4.5 Gy) that were injected with $5x10^5$ BM cells from non-induced compound R26/AE donors and exposed to DOX for 20-22 months (N=4). In all experiments cohorts of mice that were reconstituted in the same fashion but not exposed to DOX were used as controls. Number of DOX-induced and control mice analysed in each experiment are indicated either in the Figure legends or in the tables of Supplementary Figures.

RNA-Seq

Cell sorting: GMP (4 x 10^4 $L^-K^+S^+IL7R\alpha^-CD34^+Fc\gamma RII/III^+$ cells) from non-induced compound R26/AE controls (Ctrl-GMP) or C57BL/6 recipients (2 x 5.5 Gy) that were injected with 5 x 10^5 non-induced R26/AE BM cells and exposed to DOX for ten days (ST-GMP) were sorted using a FACSAria cell sorter (BD). To obtain long-term DOX-induced leukaemic GMP (L-GMP), 5 x 10^5 BM cells from R26/AE-reconstituted C57BL/6 recipients that had been exposed to DOX for 18 month were injected into secondary C57BL/6 recipients (2 x 5.5 Gy) and the DOX regime was continued for an additional four month. At this point manifest leukaemia was confirmed by inspection of peripheral blood films (presence of circulating blasts), high WBC counts and splenomegaly. For obtaining L-GMP cells for the following RNA-Seq experiments, 4 x 10^4 $L^-K^+S^+IL7R\alpha^-CD34^+Fc\gamma RII/III^+$ cells were preparatively sorted using a FACSAria cell sorter (BD). In all cases sorting efficiencies were confirmed by re-analysis of each sample (purity > 97%).

Next generation sequencing: RNA was extracted with the Pico Pure RNA Extraction Kit (Arcturus) according to the manufacturer’s instructions. The resulting RNA was analysed

using an Agilent Bioanalyzer. RNA samples were amplified with Nugen`s Ovation RNA-Seq System and quantified by Qubit measurement.

Library construction: The TruSeq DNA Sample PrepKit (Illumina) was used and the resulting cDNAs were fragmented using AFA technology (Covaris). Subsequently, cDNA fragments were end repaired and 3` adenylated. Illumina`s TruSeq adaptors with integrated indices were ligated and the resultant fragments size selected using 2% E-Gels (Invitrogen). Enrichment was done by linear PCR and the resulting fragments were purified using Agencourt AMPure XP magnetic beads. Barcoded RNA-Seq libraries were clustered on the cBot using Truseq SR cluster kit v2.5. Fifty plus bp single end reads were obtained on the Illumina HiSeq2000 using Truseq SBS kit-HS 50 bp (Illumina).

Bioinformatics and data pre-processing: Raw output data was pre-processed according to the Illumina standard protocol including filtering for low quality reads and de-multiplexing.

RNA-Seq data acquisition: Sequence reads were aligned to the mm9 reference mouse genome sequence (Waterston et al, 2002) using bowtie (Langmead et al, 2009). Alignment coordinates were compared to the exon coordinates of RefSeq transcripts (Pruitt et al, 2007) and for each transcript the counts of overlapping alignments were recorded. Sequence reads that were not aligned to the genomic sequence were aligned to a database of all possible exon-exon junction sequences of the RefSeq transcript deposit. Finally, counts of reads aligning to splice junctions were aggregated with the respective transcript counts and normalized to RPKM (number of reads which map per kilobase of exon model per million mapped reads (Mortazavi et al, 2008)). The count data was analyzed for differential expression using the R/Bioconductor package DESeq (Anders & Huber, 2010) and normalized by edgeR (Robinson et al, 2010). Pathway analysis was performed with the Ingenuity IPA software and scatter plots were generated using ANTHEUS (Build 3.3.66).

Isolation and functional characterization of LSC

To obtain long-term DOX-induced L-HSC and L-GMP, 5 x 10^5 BM cells from R26/AE-reconstituted C57BL/6 recipients that had been exposed to DOX for 18 month were injected into secondary C57BL/6 recipients (2 x 5.5 Gy) that were kept on DOX. Four month later all animals were sacrificed and presented signs of leukaemic disease indicated by the presence of circulating blasts, high WBC counts and splenomegaly. To isolate L-HSC (defined as $L^-K^+S^+$ $CD150^+$) and L-GMP (defined as $L^-K^+S^+IL7R\alpha^-CD34^+Fc\gamma RII/III^+$) whole BM of the sacrificed animals was subjected to preparative FACS using a FACSAria cell sorter (BD). In all cases sorting efficiencies were confirmed by re-analysis of each sample (purity > 97%). To test the potential of L-HSC and L-GMP for initiating and maintaining leukaemia (LSC-assay), we injected 1 x 10^3 L-HSC (9 recipient mice) or 2 x 10^4 GMP (10 recipient mice) together with 2.5x10^5 supportive C57BL/6 BM cells into C57BL/6 (2 x 5.5 Gy) animals that were continued on DOX. Recipient mice were bled seven weeks post transplantation and reconstitution was confirmed by analysing GFP expression in peripheral granulocytes, B and T cells. Five month after the transfer all animals were analysed. FACS sorted L-HSC and L-GMP from age-matched R26/AE BM-reconstituted mice that were treated in the same manner as described above but had never been exposed to DOX were used as control cells in the LSC-assay.

Acute ablation of AE expression in leukemic mice

In these experiments 5 x 10^5 BM cells from R26/AE-reconstituted C57BL/6 recipients that had been exposed to DOX for 18 month were injected into secondary C57BL/6 recipients (2 x 5.5 Gy) and continued on DOX. Four month later all animals were sacrificed and presented signs of leukaemic disease indicated by the presence of circulating blasts, high WBC counts and splenomegaly. To be able to investigate the effect of AE ablation in mice that individually

progressed towards leukaemia and thus might have acquired different secondary mutations, we used three different sacrificed mice as BM donors and ten individual mice as recipients. In each case 2.5 x 10^5 leukemic BM cells were co-injected together with 2.5 x 10^5 supportive C57BL/6 BM cells into a single C57BL/6 mouse (2 x 5.5 Gy) and all animals were continued on DOX for an additional three month. At this point seven mice were switched to a DOX-free diet and three mice were continued on DOX. Four month later all animals were analysed. Age-matched R26/AE BM-reconstituted mice that were treated in the same manner as described above but had never been exposed to DOX were used as controls.

Colony-forming unit assay

Bone marrow cells (1x10^4) of transplanted control and eight months AE-expressing recipients were seeded into methylcellulose-containing media (MethoCult 3434; StemCell Technologies). The cultures were plated in triplicate and incubated with 5% CO_2 at 37°C. After eight days, colonies were scored according to the manufacturer's instructions. In each case three control and three AE-expressing animals were analysed.

Routine haematologic assays and histopathology

Peripheral blood counts were performed on an ADVIA120 Haematology Analyzer (Bayer) or an HAEMAVET HV950 (Drew). Peripheral blood films were air dried and subsequently stained with Giemsa. Organs were formalin-fixed (4% PBS buffered formaldehyde) embedded in paraffin, sectioned and stained with haematoxylin and eosin according to standard protocols. Images were generated using a BX45 Microscope equipped with a C4040 digital camera (Olympus) and imported into Photoshop 7.0 (Adobe Systems).

Conversion and comparison of murine ST AE- and leukaemia-associated transcripts with human t(8;21) CBF AML and non-leukaemic profiles

For converting mouse RefSeq identifiers to orthologous Human Genome U133 Plus 2.0 Affymetrix IDs BioMart was used (http://www.biomart.org). To compare mouse and human gene expression profiles, confirmed t(8;21) AML and non-leukaemic gene expression signatures were extracted from GSE17855 (Balgobind et al, 2011), GSE6891 and GSE22056 (de Jonge et al, 2010; Verhaak et al, 2009) and GSE15061 (Mills et al, 2009) and normalized using the RMA procedure (Irizarry et al, 2003). To select L-GMP-specific genes that were absent or poorly expressed in human HSC, progenitors and normal blood cells, gene expression signatures from GSE30377 (Eppert et al, 2011) were extracted and normalized as above. For gene set enrichment analysis we applied the GSEA software package (Subramanian et al, 2005) and for determining the expression of genes in normal embryonic and adult tissues the Gene Enrichment Profiler program (Benita et al, 2010) was used.

REFERENCES FOR MATERIALS AND METHODS

Anders S, Huber W (2010) Differential expression analysis for sequence count data. *Genome Biol* **11:** R106

Asou H, Tashiro S, Hamamoto K, Otsuji A, Kita K, Kamada N (1991) Establishment of a human acute myeloid leukemia cell line (Kasumi-1) with 8;21 chromosome translocation. *Blood* **77:** 2031-2036

Balgobind BV, Van den Heuvel-Eibrink MM, De Menezes RX, Reinhardt D, Hollink IH, Arentsen-Peters ST, van Wering ER, Kaspers GJ, Cloos J, de Bont ES, Cayuela JM, Baruchel A, Meyer C, Marschalek R, Trka J, Stary J, Beverloo HB, Pieters R, Zwaan CM, den Boer ML (2011) Evaluation of gene expression signatures predictive of cytogenetic and molecular subtypes of pediatric acute myeloid leukemia. *Haematologica* **96:** 221-230

Benita Y, Cao Z, Giallourakis C, Li C, Gardet A, Xavier RJ (2010) Gene enrichment profiles reveal T-cell development, differentiation, and lineage-specific transcription factors including ZBTB25 as a novel NF-AT repressor. *Blood* **115:** 5376-5384

de Jonge HJ, Valk PJ, Veeger NJ, ter Elst A, den Boer ML, Cloos J, de Haas V, van den Heuvel-Eibrink MM, Kaspers GJ, Zwaan CM, Kamps WA, Löwenberg B, de Bont ES (2010) High VEGFC expression is associated with unique gene expression profiles and predicts adverse prognosis in pediatric and adult acute myeloid leukemia. *Blood* **116:** 1747-1754

Eppert K, Takenaka K, Lechman ER, Waldron L, Nilsson B, van Galen P, Metzeler KH, Poeppl A, Ling V, Beyene J, Canty AJ, Danska JS, Bohlander SK, Buske C, Minden MD, Golub TR, Jurisica I, Ebert BL, Dick JE (2011) Stem cell gene expression programs influence clinical outcome in human leukemia. *Nat Med* **17:** 1086-1093

Irizarry RA, Bolstad BM, Collin F, Cope LM, Hobbs B, Speed TP (2003) Summaries of Affymetrix GeneChip probe level data. *Nucleic Acids Res* **31:** e15

Langmead B, Trapnell C, Pop M, Salzberg SL (2009) Ultrafast and memory-efficient alignment of short DNA sequences to the human genome. *Genome Biol* **10:** R25

Mills KI, Kohlmann A, Williams PM, Wieczorek L, Liu WM, Li R, Wei W, Bowen DT, Loeffler H, Hernandez JM, Hofmann WK, Haferlach T (2009) Microarray-based classifiers and prognosis models identify subgroups with distinct clinical outcomes and high risk of AML transformation of myelodysplastic syndrome. *Blood* **114:** 1063-1072

Mortazavi A, Williams BA, McCue K, Schaeffer L, Wold B (2008) Mapping and quantifying mammalian transcriptomes by RNA-Seq. *Nat Methods* **5:** 621-628

Pruitt KD, Tatusova T, Maglott DR (2007) NCBI reference sequences (RefSeq): a curated non-redundant sequence database of genomes, transcripts and proteins. *Nucleic Acids Res* **35:** D61-65

Robinson MD, McCarthy DJ, Smyth GK (2010) edgeR: a Bioconductor package for differential expression analysis of digital gene expression data. *Bioinformatics* **26:** 139-140

Schmittgen T (2001) Real-time quantitative PCR. *Methods* **25:** 383-385

Subramanian A, Tamayo P, Mootha VK, Mukherjee S, Ebert BL, Gillette MA, Paulovich A, Pomeroy SL, Golub TR, Lander ES, Mesirov JP (2005) Gene set enrichment analysis: a knowledge-based approach for interpreting genome-wide expression profiles. *Proc Natl Acad Sci U S A* **102:** 15545-15550

Verhaak RG, Wouters BJ, Erpelinck CA, Abbas S, Beverloo HB, Lugthart S, Löwenberg B, Delwel R, Valk PJ (2009) Prediction of molecular subtypes in acute myeloid leukemia based on gene expression profiling. *Haematologica* **94:** 131-134

Waterston RH, Lindblad-Toh K, Birney E, Rogers J, Abril JF, Agarwal P, Agarwala R, Ainscough R, Alexandersson M, An P, Antonarakis SE, Attwood J, Baertsch R, Bailey J, Barlow K, Beck S, Berry E, Birren B, Bloom T, Bork P, Botcherby M, Bray N, Brent MR, Brown DG, Brown SD, Bult C, Burton J, Butler J, Campbell RD, Carninci P, Cawley S, Chiaromonte F, Chinwalla AT, Church DM, Clamp M, Clee C, Collins FS, Cook LL, Copley RR, Coulson A, Couronne O, Cuff J, Curwen V, Cutts T, Daly M, David R, Davies J, Delehaunty KD, Deri J, Dermitzakis ET, Dewey C, Dickens NJ, Diekhans M, Dodge S, Dubchak I, Dunn DM, Eddy SR, Elnitski L, Emes RD, Eswara P, Eyras E, Felsenfeld A, Fewell GA, Flicek P, Foley K, Frankel WN, Fulton LA, Fulton RS, Furey TS, Gage D, Gibbs RA, Glusman G, Gnerre S, Goldman N, Goodstadt L, Grafham D, Graves TA, Green ED, Gregory S, Guigó R, Guyer M, Hardison RC, Haussler D, Hayashizaki Y, Hillier LW, Hinrichs A, Hlavina W, Holzer T, Hsu F, Hua A, Hubbard T, Hunt A, Jackson I, Jaffe DB, Johnson LS, Jones M, Jones TA, Joy A, Kamal M, Karlsson EK, Karolchik D, Kasprzyk A, Kawai J, Keibler E, Kells C, Kent WJ, Kirby A, Kolbe DL, Korf I, Kucherlapati RS, Kulbokas EJ, Kulp D, Landers T, Leger JP, Leonard S, Letunic I, Levine R, Li J, Li M, Lloyd C, Lucas S, Ma B, Maglott DR, Mardis ER, Matthews L, Mauceli E, Mayer JH, McCarthy M, McCombie WR, McLaren S, McLay K, McPherson JD, Meldrim J, Meredith B, Mesirov JP, Miller W, Miner TL, Mongin E, Montgomery KT, Morgan M, Mott R, Mullikin JC, Muzny DM, Nash WE, Nelson JO, Nhan MN, Nicol R, Ning Z, Nusbaum C, O'Connor MJ, Okazaki Y, Oliver K, Overton-Larty E, Pachter L, Parra G, Pepin KH, Peterson J, Pevzner P, Plumb R, Pohl CS, Poliakov A, Ponce TC, Ponting CP, Potter S, Quail M, Reymond A, Roe BA, Roskin KM, Rubin EM, Rust AG, Santos R, Sapojnikov V, Schultz B, Schultz J, Schwartz MS, Schwartz S, Scott C, Seaman S, Searle S, Sharpe T, Sheridan A, Shownkeen R, Sims S, Singer JB, Slater G, Smit A, Smith DR, Spencer B, Stabenau A, Stange-Thomann N, Sugnet C, Suyama M, Tesler G, Thompson J, Torrents D, Trevaskis E, Tromp J, Ucla C, Ureta-Vidal A, Vinson JP, Von Niederhausern AC, Wade CM, Wall M, Weber RJ, Weiss RB, Wendl MC, West AP, Wetterstrand K, Wheeler R, Whelan S, Wierzbowski J, Willey D, Williams S, Wilson RK, Winter E, Worley KC, Wyman D, Yang S, Yang SP, Zdobnov EM, Zody MC,

Lander ES, Consortium MGS (2002) Initial sequencing and comparative analysis of the mouse genome. *Nature* **420:** 520-562
